# Nonlinear Vibrations and Potential Instabilities of a Nanochassis Traveling a Route with Arbitrarily Tiny Irregularities

**DOI:** 10.3390/nano16120768

**Published:** 2026-06-18

**Authors:** Banghua Xie, Kai Wu, Ali Nikkhoo

**Affiliations:** 1 School of Civil Engineering & Architecture, Jiangxi University of Water Resources and Electric Power, Nanchang 330099, China; 2 Jiangxi Key Laboratory of Structural Safety of Water Conservancy and Hydropower Engineering, Nanchang 330099, China; 3 Faculty of Engineering, Departement of Civil Engineering, University of Science and Culture (USC), Tehran 1461968151, Iran

**Keywords:** nonlinear nonlocal vibrations, moving nanochassis, surface effect, nonlocal integro-based beam model, surface with small irregularities, possible dynamic instabilities

## Abstract

Free vibrations of axially moving beam-like nanostructures have been investigated in recent years; however, vibrations of moving nanochassis traveling over a surface with arbitrarily small irregularities have not been displayed yet due to some complexities in modeling. To address this challenge, a nonlinear, nonlocal surface energy-based composite beam-like model is established to fairly accurately capture the nanochassis’ vibrations. The nanocar consists of a composite-like nanochassis and the ends’ wheels, where the nanochassis is modeled by an appropriate beam model and the wheels are simulated as rigid solid elements that are attached to the beam’s ends. Both differential- and integral-based formulations are presented, and their nonlinear stiffness, as well as the procedure for capturing the nonlocal elastic field, is carefully explained using the assumed mode approach. For several particular cases, the predicted results by the suggested models are verified with those of several analytical solutions, and reasonably good agreements are achieved. Beyond the aforementioned comparison studies, the possible instabilities of the nanochassis that travels over a straight route were also identified and explained under a small deformation regime. Through conducting a fairly comprehensive parametric study, the roles of amplitude and frequencies of the harmonic route, axial velocity, length, diameter, nonlocality, surface energy, and geometrical nonlinearity on maximum deformations and internal forces are examined comprehensively. This study could be considered as basic scrutiny for the nonlinear analysis of more complex traveling nanostructures over arbitrarily shaped surfaces.

## 1. Introduction

In recent years, the design of mechanically controlled machines at the atomic scale has led to the fabrication of various molecular-based motors [1,2], rotors [3,4,5], and elevators [6,7], nanocars and nanovehicles [8,9,10,11]. The latter one presents a novel class of nanomachines consisting of molecular chassis, axles, and wheels that can even pass over the surface of solids with specific directions. These nanovehicles convert the electrical field, applied thermal field, or light energy into the mechanical and controlled movement to transport special information or materials from one place to another. The above-mentioned crucial capability of these tiny moving cars (i.e., structurally specified motion) has gained the attention of scientists from various disciplines including chemists, surface engineers, applied physics, and mechanics. On the other hand, vibration analysis of a moving nanochassis over microscopically irregular routes requires a framework that can simultaneously capture size dependence, surface energy, and instability-sensitive dynamics. Related developments in flexible structural dynamics, instability theory, and vibration-sensitive mechanical response could provide useful methodological context [12,13,14,15]. On the other hand, periodic resonant cavities can effectively tailor low-frequency vibrations [16], while droplet deformation under airflow reveals flow-induced mechanical instabilities [17], which, in a combination of these phenomena (i.e., resonance due to the excitations of ends’ nanochassis as well as reduction in flexural stiffness due to its moving nature), occur in moving nanochassis problems. In addition, biomechanical investigations of footwear effects on impact forces and soft-tissue vibrations [18,19] can also provide valuable information on possible dynamic instabilities in a moving nanochassis under route irregularities. Until now, free transverse vibrations of nanoscaled beam-like structures with axial motion have been widely examined; however, nonlinear vibrations of beam-like nanochassis with potential movement over a surface with very tiny irregularities have not been methodically researched yet. This issue encouraged the authors to focus on the mechanical aspects of moving nanochassis through developing and analyzing appropriate models to display both longitudinal and transverse vibrations and instabilities in some detail. For this, previous works on nonlinear forced vibrations of beam-like structures, from macro to nano [20,21,22,23], should be carefully examined to ensure the reliability and convergence of the employed approach.

At the atomic level, the induced stress at a point would no longer be local since vibrations of each atom rely on the vibrations of its adjacent atoms. The so-called *nonlocality* be captured by the classical elasticity theory (CET) since it displays that the stress field at each point of the continuum only depends on the state of the stress on that point. To overcome this disadvantage of the CET, nonlocal elasticity theory (NET) was wisely developed by Eringen [24,25,26,27] in the last decades of the previous century. After application of the NET to statics and buckling analyses of beam-like nanostructures by Peddieson et al. [28] and Sudak [29], many researchers commenced exploiting the nonlocal rod, beam, plate, and shell models for examining buckling [30,31,32,33], postbuckling [34,35,36], linear vibration [37,38,39,40,41,42], and nonlinear vibrations [43,44,45,46] of nanostructures. The employed models can be classified into two major groups: (i) nonlocal differential-based models, (ii) nonlocal integro-based models. In the first group, the nonlocal stresses can be simply related to the local stresses in the Cartesian coordinate system by: $\sigma_{ij}^{nl}-{\left(e_{0}a\right)}^{2}\nabla^{2}\sigma_{ij}^{nl}=\sigma_{ij}^{l}$, where $e_{0}a$ denotes the small-scale factor, ∇ is the nabla symbol, $\sigma_{ij}^{l}$ and $\sigma_{ij}^{nl}$ in order are the local and the nonlocal stress components. Such a constitutive relation was initiated by Eringen [27] to reduce the complexities arising from the nonlocal integro-based modeling of continuum fields, particularly those whose spatial domains are infinite. So far, this special form of the nonlocality has been extensively employed by various investigators for examining free vibrations of nanobeams and nanoplates [47,48,49,50,51,52,53,54]. For the models of the second group, the constitutive relations are generally stated by [27]: $\sigma_{ij}^{nl}\left(s,t\right)=\int_{\Omega }\;K(|s-s^{\prime }\left|;l_{s}\right)\;\sigma_{ij}^{l}\left(s^{\prime },t\right)\;dV^{\prime }$, where $l_{s}$ signifies the small-scale parameter, |[.]| represents the norm symbol of the vector [.], *K* is an appropriate kernel function (1D, 2D, or 3D depends on the size of the computational domain of the continuum of our concern, Ω), $dV^{\prime }$ denotes the volume of an infinitesimal element from the spatial domain, and *t* is the time parameter. In recent years, the nonlocal integro-based models have been widely applied to the vibrational problems of nanostructures [55,56,57,58,59]. In nanobeams, the paradoxical behavior of differential nonlocal models refers to counterintuitive predictions where increasing the nonlocal parameter produces apparent hardening instead of the expected softening. This mainly arises because the differential formulation requires additional constitutive boundary conditions for bounded domains. When these are omitted or inconsistently applied, anomalous size effects and contradictory responses occur. Integral and stress-driven nonlocal models generally eliminate these inconsistencies and recover physically consistent softening behavior. Such great achievements have been a powerful motivation for investigators who are applying the nonlocal integro-based models to unresolved problems of nanomechanics.

Concerning nonlocal vibrations of moving nanostructures, Lim et al. [60] investigated transverse free vibration of axially moving nanoscaled beams by application of the nonlocal continuum theory of Eringen to Euler–Bernoulli beam theory. Using the variational principle, novel higher-order differential equations of motion were derived accounting for higher-order as well as non-classical boundary conditions. Kiani [61] examined axial and lateral vibrations of nanobeams made from functionally graded materials with axial movement using the nonlocal Rayleigh beam theory. By application of the Galerkin method to the nonlocal governing equations, the longitudinal and transverse natural frequencies were evaluated, and the flutter and divergence instabilities with their pertinent influential factors were displayed and discussed. Li [62] studied free transverse vibrations of axially moving piezoelectric Euler–Bernoulli-based nanobeams accounting for nonlocal thermoelectro–mechanical coupling. Using eigenvalue approach, the natural frequencies for both simple–simple and clamped–clamped ends were evaluated and their influential parameters were displayed and discussed in the presence of the applied thermal, mechanical, and electrical fields. Wang et al. [63] explored free vibration of transverse motion of axially moving nanobeams by exploiting the nonlocal strain gradient theory. The roles of instability conditions and size effect on frequencies and critical velocities of various vibration modes were explained by employing the Euler beam model. Mokhtari et al. [64] examined free transverse vibration of nanobeams moving in axial direction by implementing the nonlocal Euler-based beam theory. The constitutive equations of motion were analyzed for natural frequencies by employing wavelet-based spectral element method. The effects of the axial velocity, pre-stressing load, and nonlocality on the free vibration trends of the moving nanostructures were displayed in some details. Guo et al. [65] investigated dynamic deflection and its potential instability for nanobeams with both axial movement and rotatory motion using the nonlocal strain gradient-based continuum mechanics. The roles of nonlocality, strain gradient effect, rotary motion, and axial velocity on the lateral stiffness of the rotating-moving nanostructure were revealed and discussed. This brief literature review indicates that all carried out explorations on axially moving nonlocal-based nanostructures were limited to the linear and free vibrations. For the most general form of the translational motion, Kiani [66,67] examined three-dimensional vibrations of moving nanocables with three components for the velocity and acceleration vector fields. The roles of nonlocality, surface energy, and speeds in various directions on the divergence instability, vibrations, and static deflections of the traveling nanocable were studied in some detail.

For nanoscale structures like nanowires, nanochassis, nanorods, nanoplates, and nanoshells, the ratio of the surface’s area to the bulk’s volume is much higher than their corresponding macro-scaled ones. On the other hand, conducted research works by Gurtin and Murdoch [68,69] revealed that the mechanical behaviors of the surface layer are entirely different from those of the bulk. To include this in the formulations of motion of tiny structures, they established a novel surface elasticity-based theory based on a new description of strain–stress relations for surface layers. For instance, they proposed three material constants (i.e., residual stress within the very thin surface layer, $\tau_{s}$, and two mechanical constants, $\lambda_{s}$, and $\mu_{s}$) to display the constitutive relations of isotropic layers. These constants are commonly determined by adjusting the dispersion curves from the surface energy-based model for tiny bars and plates and those predicted by a suitable atomic-based methodology [70,71]. Up until now, the continuum-based model of Gurtin–Murdoch has been implemented for many nanomechanical problems raised in the field of nanotechnology including free vibration [72,73,74,75], forced vibration [76,77,78], nonlinear vibrations [79,80] of both nanobeams and nanoplates. Because the present work emphasizes a nonlinear nonlocal surface-energy-based beam-like model, it is also reasonable to position the study alongside recent advances in memory-dependent constitutive modeling, surface-governed nanoscale behavior, and mechanics-informed numerical formulations [81,82,83,84]. In doing so, applications of these more inclusive models to the present problem can lead to more inclusive models and can be considered for upcoming research work. Regarding vibrations of moving nanostructures accounting for the surface energy effect, Kiani [85] studied flutter and divergence instabilities of axially moving nanobeams by exploiting surface energy-based Rayleigh, Timoshenko, and higher-order beam theories. The natural frequencies were computed for elastically embedded moving nanobeams by employing Galerkin-based admissible modes methodology. Recently, Aichun and Kiani [86] examined free vibrations of axially moving piezoelectric nanowires with the surface effect based on the Rayleigh and Timoshenko beam models. The explicit terms of the divergence velocity as well as the critical applied voltage were introduced, and the impacts of crucially displayed factors on their free vibrations and potential instabilities were revealed in some details. As it is clearly recognized, surface energy-based vibrations of moving beam-like nanochassis with excited ends have not been cultivated yet, and all the previous studies were limited to the linear case.

In the present work, nonlinear axial and transverse vibrations of movable beam-like nanochassis over a surface with arbitrary irregularity are of particular concern. The problem analyzed in such generality could also elucidate the vibrational mechanism underlying nanoscale chassis motion on a surface with arbitrary irregularities. To this end, the nanomechanical problem is described in Section 2 for the first time. A nonlocal differential surface energy-based model and a nonlocal integro-based model (NDS and NIS) are, respectively, established in Section 3 and Section 4. It should be highlighted herein that the proposed approaches based on the NDS or NIS are essentially related to the Cauchy tensor because they utilize the classical local Cauchy stress tensor as the base field, then transform it into a nonlocal stress tensor through differential-based or integral-based operators, respectively, and combine it with surface elasticity effects; these stress measures are what generate the model’s effective stiffness. For the proposed models, the nonlinear governing equations are derived carefully, and then, these are solved for the unknown dynamical axial displacement, deflection, axial force, and flexural moment. In Section 5, several analytical solutions for both free vibrations of axially moving nanochassis and forced vibrations of immovable nanochassis acted upon by ends’ excitation are developed, and the obtained results by the numerical models mentioned above are appropriately checked with these analytical ones. In continuing, an inclusive parametric study is presented and the crucial roles of the nanochassis geometry, characteristics of ends’ excitations, speed, nonlocality, geometrical nonlinearity, and surface energy on the axial and lateral vibrations of such moving nanostructures are displayed and discussed. Section 6 displays the prospects of the present research work and several hot topics for future works. Finally, the notable results obtained are presented in Section 7.

## 2. A Brief Outline of the Nanoengineering Problem

**Figure 1:**
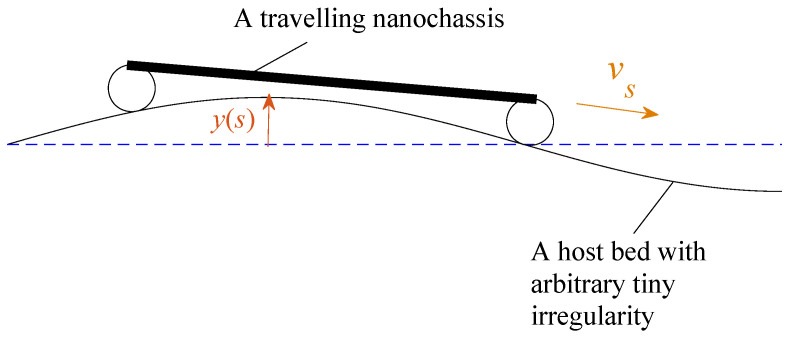
A beam-like nanochassis moves over an arbitrarily shaped route with small irregularities ((−−) imaginary horizontal-flat surface, (—) uneven surface).

Consider a continuum-based beam-like nanochassis of length $l_{b}$ and height $h_{0}$ moves horizontally at a constant velocity V over an arbitrarily shaped surface as demonstrated in Figure 1. Without loss of generality, it is assumed that the surface along the horizontal would be sinusoidal with the following form: $y\left(s\right)=a_{0}\;\sin\left(\frac{2\pi s}{L}\right)$, where $a_{0}$ is the amplitude of the harmonic route and *L* denotes its wavelength (see Figure 1). Since only nonlinear longitudinal and transverse vibrations of the moving nanochassis over a rough route are of our concern in this study, we considered the ends’ wheels as attached massless rigid objects that only transfer the induced transverse vibrations to the main nanochassis. However, for a more systematic modeling of the problem, particularly when the roles of the mechanical properties of the attached wheels (mass, stiffness, and damping as the crucial factors) on the vibrations of the moving nanochassis is of interest, one can model the motion of the nanochassis, wheels, and nanosystems traversing a rough route with more details through developing a more systematic mathematical model.

Under the premise of the assumption mentioned above, when $a_{0}\ll l_{b}$ and $l_{b}\ll L$, it can be easily proved that the problem of moving nanostructures on a harmonic surface can be reduced to a moving nanoscaled beam under excitation $W_{g_{1}}\left(t\right)$ and $W_{g_{2}}\left(t\right)$ at its ends such that: $W_{g_{1}}\left(t\right)=a_{0}\;\sin\left[2\pi \left(\frac{V_{s}}{L}\;t+\frac{l_{1}}{L}\right)\right]$ and $W_{g_{2}}\left(t\right)=a_{0}\;\sin\left[2\pi \left(\frac{V_{s}}{L}\;t+\frac{l_{2}}{L}\right)\right]$, where $l_{2}$ = $l_{1}+l_{b}$, $V_{s}$ represents the horizontal component of the velocity (please see Appendix A). Since $a_{0}\ll L$, it can be rationally concluded that $V_{s}\approx V$. Additionally, the last term inside the bracket of $W_{g_{2}}$ could be omitted since $l_{b}\ll L$. In such a case, the excitations at both ends of the moving nanochassis would be identical. From an application viewpoint, traveling nanostructures subjected to tiny geometric irregularities may experience route-induced amplification, bifurcation-like responses, and safety-critical dynamic transitions. Comparable concerns appear in studies of transport safety, flow-controlled systems, and deformation-sensitive solid media, which together help motivate broader engineering relevance [87,88,89,90]. By these virtues, we are also eagerly interested in responding this critical question: *how combinational characteristics of surfaces with harmonic irregularity and moving nanochassis could endanger its lateral vibration?*

In the following parts, we examine the nonlinear axial and lateral vibrations of a nanochassis travels over a surface with arbitrarily small irregularities. To this end, the nonlinear equations of motion of the moving nanostructure are carefully derived using both nonlocal Rayleigh-based beam theory by considering the surface effect. The nonlocality is incorporated into the formulations of the problem by establishing two novel models: *nonlocal differential* and *nonlocal integro-type*.Then, these coupled equations are solved for axial and lateral dynamic deformations by employing the Galerkin approach based on admissible modes. The details of nonlinear calculations for each model based on the Newton’s method are then displayed, and the obtained results will be then presented and discussed in some detail.

## 3. Nonlocal Differential-Based Modeling of Moving Excited Nanochassis

### 3.1. Nonlinear Equations of Motion

Through deploying the nonlocal differential-based elasticity model of Eringen [24,25,26], the most important nonlocal normal stresses of the bulk and the surface layer (i.e., $\sigma_{ss,b}^{nl}$ and $\sigma_{ss,s}^{nl}$) are linked to their corresponding local values (i.e., $\sigma_{ss,b}^{l}$ and $\sigma_{ss,s}^{l}$) by:

(1) $$\begin{array}{c} \sigma_{ss,b}^{nl}-{\left(e_{0}a\right)}_{b}^{2}\frac{\partial^{2}\sigma_{ss,b}^{nl}}{\partial s^{2}}=\sigma_{ss,b}^{l},\;\\ \sigma_{ss,s}^{nl}-{\left(e_{0}a\right)}_{s}^{2}\frac{\partial^{2}\sigma_{ss,s}^{nl}}{\partial s^{2}}=\sigma_{ss,s}^{l}, \end{array}$$

where ${\left(e_{0}a\right)}_{\left[.\right]};\;\left[.\right]=b\;\mathrm{or}\;s$ is the nonlocal factor of the bulk or that of the surface layer. On the other hand, the nonlinear Lagrangian strain of the surface layer ($E_{ss,s}$) and that of the bulk ($E_{ss,b}$) from the moving beam-like nanochassis can be stated by:

(2) $$\begin{array}{c} E_{ss,s}=\frac{\partial X_{s,s}}{\partial s}+\frac{1}{2}\left[{\left(\frac{\partial X_{s,s}}{\partial s}\right)}^{2}+{\left(\frac{\partial X_{y,s}}{\partial s}\right)}^{2}\right],\;\\ E_{ss,b}=\frac{\partial X_{s,b}}{\partial s}+\frac{1}{2}\left[{\left(\frac{\partial X_{s,b}}{\partial s}\right)}^{2}+{\left(\frac{\partial X_{y,b}}{\partial s}\right)}^{2}\right]. \end{array}$$

Using the main hypothesis of the Rayleigh beam theory for the elastically deformable nanochassis (i.e., $X_{s,s}\left(s,y,t\right)=X_{s}\left(s,t\right)-y\;\frac{\partial Y_{s}}{\partial s}\left(s,t\right)$ and $X_{s,b}\left(s,y,t\right)=X_{b}\left(s,t\right)-y\;\frac{\partial Y_{b}}{\partial s}\left(s,t\right)$), where $X_{\left[.\right]}$ represents the axial deformation at the neutral axis, *y* denotes the distance from the neutral axis, and $Y_{\left[.\right]}$ is the lateral deformation. Since the surface layer has been stiffly attached to the bulk, they have identical axial and lateral deformations (i.e., X = $X_{s}$ = $X_{b}$ and Y = $Y_{s}$ = $Y_{b}$). By introducing these forms of deformation fields to Equation (2), assuming that the transverse deflection field would not vary across the thickness (i.e., $X_{y,b}$ = $X_{y,s}$ = Y, where Y = Y(s,t)), the nonlinear axial strains take the following form:

(3) $$\begin{array}{c} E_{ss,b}=E_{ss,s}\approx \frac{\partial X}{\partial s}+\frac{1}{2}\left[{\left(\frac{\partial X}{\partial s}\right)}^{2}+{\left(\frac{\partial Y}{\partial s}\right)}^{2}\right]-y\;\frac{\partial^{2}Y}{\partial s^{2}}, \end{array}$$

By employing Hooke’s law,

(4) $$\begin{array}{c} \sigma_{ss,b}^{l}=E_{b}\;E_{ss,b}+\nu_{b}\;\sigma_{yy,b}^{l},\; \end{array}$$

where $\nu_{b}$ is the bulk’s Poisson ratio, $E_{b}$ is the bulk’s elastic modulus, $\sigma_{\alpha \alpha ,\left[.\right]}^{l}$ represents the normal stresses within the bulk or the surface layer along the α direction (α = *s* or *y*). The requirement of satisfaction of equilibrium of the surface layer along the *y* direction leads to:

(5) $$\begin{array}{c} \tau_{s}\;\frac{\partial^{2}X_{y}^{+}}{\partial s^{2}}-{\left(\sigma_{yy}^{l}\right)}^{+}=\rho_{s}\;\frac{D^{2}X_{y}^{+}}{Dt^{2}},\;\\ \tau_{s}\;\frac{\partial^{2}X_{y}^{-}}{\partial s^{2}}+{\left(\sigma_{yy}^{l}\right)}^{-}=\rho_{s}\;\frac{D^{2}X_{y}^{-}}{Dt^{2}}, \end{array}$$

in these relations, $\frac{D[.]}{Dt}$ is the first material derivative operator (for example, for a body moves axially with the constant axial velocity of $V_{s}$, it would be $\frac{\partial [.]}{\partial t}+V_{s}\;\frac{\partial [.]}{\partial s}$), $\tau_{s}$ represents the residual stress of the surface, $\rho_{s}$ is the density of the surface layer. The elastic fields specified by the superscripts − and + in order correspond to the furthest axis at the bottom and top of the surface layer.

By accepting linear variation in the local normal stress (i.e., $\sigma_{yy,b}^{l}$) across the thickness of the bulk and using Equation (5), it is derivable:

(6) $$\begin{array}{c} \sigma_{yy,b}^{l}=\frac{2y}{h_{0}}\;\left[\tau_{s}\;\frac{\partial^{2}Y}{\partial s^{2}}-\rho_{s}\;\left(\frac{\partial^{2}Y}{\partial t^{2}}+2V_{s}\frac{\partial^{2}Y}{\partial s\partial t}+V_{s}^{2}\;\frac{\partial^{2}Y}{\partial s^{2}}\right)\right].\; \end{array}$$

By introducing Equations (5) and (6) to Equation (4), the local axial stress within the bulk as a function of deformation fields takes the following form:

(7) $$\begin{array}{cc} \sigma_{ss,b}^{l}= & E_{b}\;\left\{\frac{\partial X}{\partial s}+\frac{1}{2}\left[{\left(\frac{\partial X}{\partial s}\right)}^{2}+{\left(\frac{\partial Y}{\partial s}\right)}^{2}\right]\right\}+\;\\ & y\left[\left(\frac{2\nu_{b}\tau_{s}}{h_{0}}-E_{b}\right)\frac{\partial^{2}Y}{\partial s^{2}}-\frac{2\nu_{b}\rho_{s}}{h_{0}}\left(\frac{\partial^{2}Y}{\partial t^{2}}+2V_{s}\frac{\partial^{2}Y}{\partial s\partial t}+V_{s}^{2}\;\frac{\partial^{2}Y}{\partial s^{2}}\right)\right]. \end{array}$$

Within the framework of the Gurtin–Murdoch surface continuum-based theory, we can express the constitutive relations of the surface layer by [68,69]:

(8) $$\begin{array}{c} \sigma_{ij,s}^{l}=\tau_{s}+2\left(\mu_{s}-\tau_{s}\right)\varepsilon_{ij}+\left(\lambda_{s}+\tau_{s}\right)\varepsilon_{kk}\delta_{ij}, \end{array}$$

in which $\sigma_{ij,s}^{l}$ is the local stresses of the surface, $\delta_{ij}$ represents the Kronecker delta tensor, $\mu_{s}$ and $\lambda_{s}$ are the constants of Lam$\overset{´}{e}$ of the surface layer such that $E_{s}$ = $\lambda_{s}+2\mu_{s}$ is its elastic modulus. By virtue of Equations (1) and (3), the local axial stress field of the surface layer accounting for large deflections can be displayed by:

(9) $$\begin{array}{c} \sigma_{ss,s}^{l}=\tau_{s}+\left(\lambda_{s}+2\mu_{s}\right)\;\left\{\frac{\partial X}{\partial s}+\frac{1}{2}\left[{\left(\frac{\partial X}{\partial s}\right)}^{2}+{\left(\frac{\partial Y}{\partial s}\right)}^{2}\right]\right\}-y\left(\lambda_{s}+2\mu_{s}\right)\;\frac{\partial^{2}Y}{\partial s^{2}}, \end{array}$$

where, in the case of small deflection-small rotation, Equation (9) can be reduced to that provided in previous studies [91,92,93,94,95]. The local axial forces within the constituent parts of the moving nanochassis ($N_{s,s}^{l}$ and $N_{s,b}^{l}$) can be readily evaluated by the integration of the local axial stresses over their corresponding cross-sectional domains:

(10) $$\begin{array}{c} N_{s,s}^{l}=\int_{A_{s}}\sigma_{ss,s}^{l}dA_{s}=\tau_{s}A_{s}+\left(\lambda_{s}+2\mu_{s}\right)A_{s}\;\left\{\frac{\partial X}{\partial s}+\frac{1}{2}\left[{\left(\frac{\partial X}{\partial s}\right)}^{2}+{\left(\frac{\partial Y}{\partial s}\right)}^{2}\right]\right\},\;\\ N_{s,b}^{l}=\int_{A_{b}}\sigma_{ss,b}^{l}dA=E_{b}A_{b}\;\left\{\frac{\partial X}{\partial s}+\frac{1}{2}\left[{\left(\frac{\partial X}{\partial s}\right)}^{2}+{\left(\frac{\partial Y}{\partial s}\right)}^{2}\right]\right\}. \end{array}$$

By premultiplying Equations (7) and (9) by *y*, and taking the integral from the resulting expressions over their relevant cross-sectional domains, the local flexural moments within the major constituents of the moving nanochassis, denoted by $M_{y,s}^{l}$ and $M_{y,b}^{l}$, are calculated by:

(11) $$\begin{array}{c} M_{y,s}^{l}=\int_{A_{s}}y\;\sigma_{ss,s}^{l}dA=-I_{s}\;\left(\lambda_{s}+2\mu_{s}\right)\;\frac{\partial^{2}Y}{\partial s^{2}},\;\\ M_{y,b}^{l}=\int_{A_{b}}y\;\sigma_{ss,b}^{l}dA=I_{b}\;\left[\begin{array}{c} \left(\frac{2\nu_{b}\tau_{s}}{h_{0}}-E_{b}\right)\frac{\partial^{2}Y}{\partial s^{2}}-\frac{2\nu_{b}\rho_{s}}{h_{0}}\times \;\\ \left(\frac{\partial^{2}Y}{\partial t^{2}}+2V_{s}\frac{\partial^{2}Y}{\partial s\partial t}+V_{s}^{2}\;\frac{\partial^{2}Y}{\partial s^{2}}\right) \end{array}\right], \end{array}$$

where $I_{s}=\int_{A_{s}}y^{2}\;dA$ and $I_{b}=\int_{A_{b}}y^{2}\;dA$. By taking the integral from Equation (10) over the nanochassis’s cross-section area, the relationship between the local axial force and the nonlocal one for the constituting parts of the continuum-based nanochassis are derived as follows:

(12) $$\begin{array}{c} N_{s,s}^{nl}-{\left(e_{0}a\right)}_{s}^{2}\;\frac{\partial^{2}N_{s,s}^{nl}}{\partial s^{2}}=N_{s,s}^{l},\;\;\;\;\;N_{s,b}^{nl}-{\left(e_{0}a\right)}_{b}^{2}\;\frac{\partial^{2}N_{s,b}^{nl}}{\partial s^{2}}=N_{s,b}^{l}, \end{array}$$

additionally, by using of Equation (11), the nonlocal flexural moments within the consisting parts of the beam-like nanochassis take the following form:

(13) $$\begin{array}{c} M_{s,s}^{nl}-{\left(e_{0}a\right)}_{s}^{2}\;\frac{\partial^{2}M_{s,s}^{nl}}{\partial s^{2}}=M_{s,s}^{l},\;\;\;\;\;M_{s,b}^{nl}-{\left(e_{0}a\right)}_{b}^{2}\;\frac{\partial^{2}M_{s,b}^{nl}}{\partial s^{2}}=M_{s,b}^{l}. \end{array}$$

To construct the nonlocal nonlinear equations of motion, we exploit Hamilton’s principle. For this purpose, the kinetic energy of the axially moving nanochassis, $\Pi_{k}\left(t\right)$, as well as its strain energy by considering the nonlocality and the surface energy effect, $\Pi_{e}\left(t\right)$, can be represented by:

(14a) $$\begin{array}{c} \begin{array}{c} \Pi_{k}\left(t\right)=\frac{1}{2}\;\int_{\Omega }\left[\rho_{b}\left({\left(\frac{DX_{s}}{Dt}\right)}^{2}+{\left(\frac{DX_{y}}{Dt}\right)}^{2}\right)\right]\;d\Omega +\frac{1}{2}\;\int_{\Omega_{s}}\rho_{s}\left({\left(\frac{DX_{s}}{Dt}\right)}^{2}+{\left(\frac{DX_{y}}{Dt}\right)}^{2}\right)\;d\Omega , \end{array} \end{array}$$

(14b) $$\begin{array}{c} \begin{array}{c} \Pi_{e}\left(t\right)=\frac{1}{2}\;\int_{\Omega_{b}}\sigma_{ss,b}^{nl}E_{ss,b}\;d\Omega +\frac{1}{2}\;\int_{\Omega_{s}}\sigma_{ss,s}^{nl}E_{ss,s}\;d\Omega , \end{array} \end{array}$$

or in a more detailed form as follows:

(15a) $$\begin{array}{c} \begin{array}{c} \Pi_{k}\left(t\right)=\frac{1}{2}\;\int_{0}^{l_{b}}\left\{\begin{array}{c} \left(\rho_{b}A_{b}+\rho_{s}A_{s}\right)\left[{\left(\frac{\partial X}{\partial t}+V_{s}\frac{\partial X}{\partial s}\right)}^{2}+{\left(\frac{\partial Y}{\partial t}+V_{s}\frac{\partial Y}{\partial s}\right)}^{2}\right]+\;\\ \left(\rho_{b}I_{b}+\rho_{s}I_{s}\right){\left(\frac{\partial^{3}Y}{\partial s^{2}\partial t}+V_{s}\frac{\partial^{3}Y}{\partial s^{3}}\right)}^{2} \end{array}\right\}\;ds, \end{array} \end{array}$$

(15b) $$\begin{array}{c} \begin{array}{c} \Pi_{e}\left(t\right)=\frac{1}{2}\int_{0}^{l_{b}}\left\{\left\{\frac{\partial X}{\partial s}+\frac{1}{2}\left[{\left(\frac{\partial X}{\partial s}\right)}^{2}+{\left(\frac{\partial Y}{\partial s}\right)}^{2}\right]\right\}\;\left(N_{s,s}^{nl}+N_{s,b}^{nl}\right)-\frac{\partial^{2}Y}{\partial s^{2}}\;\left(M_{y,s}^{nl}+M_{y,b}^{nl}\right)\right\}\;ds. \end{array} \end{array}$$

By exploiting the Hamilton’s principle, $\int_{t_{1}}^{t_{2}}\left(\delta \Pi_{k}\left(t\right)-\delta \Pi_{e}\left(t\right)\right)dt$ = 0, the equations of axial and lateral motions of the moving nanochassis with large deformations are obtained as follows:

(16a) $$\begin{array}{c} \begin{array}{c} \left(\rho_{b}A_{b}+\rho_{s}A_{s}\right)\;\left(\frac{\partial^{2}X}{\partial t^{2}}+2V_{s}\frac{\partial^{2}X}{\partial s\partial t}+V_{s}^{2}\frac{\partial^{2}X}{\partial s^{2}}\right)-\frac{\partial N_{s,b}^{nl}}{\partial s}-\frac{\partial N_{s,s}^{nl}}{\partial s}=0, \end{array} \end{array}$$

(16b) $$\begin{array}{c} \begin{array}{c} \left(\rho_{b}A_{b}+\rho_{s}A_{s}\right)\;\left(\frac{\partial^{2}Y}{\partial t^{2}}+2V_{s}\frac{\partial^{2}Y}{\partial s\partial t}+V_{s}^{2}\;\frac{\partial^{2}Y}{\partial s^{2}}\right)-\left(\rho_{b}I_{b}+\rho_{s}I_{s}\right)\times \;\\ \left(\frac{\partial^{4}Y}{\partial t^{2}\partial s^{2}}+2V_{s}\frac{\partial^{4}Y}{\partial s^{3}\partial t}+V_{s}^{2}\;\frac{\partial^{4}Y}{\partial s^{4}}\right)-\frac{\partial }{\partial s}\;\left(\left(N_{s,s}^{nl}+N_{s,b}^{nl}\right)\frac{\partial Y}{\partial s}\right)-\frac{\partial^{2}M_{y,b}^{nl}}{\partial s^{2}}-\frac{\partial^{2}M_{y,s}^{nl}}{\partial s^{2}}=0. \end{array} \end{array}$$

Through mixing Equations (12) and (13) with Equations (16a) and (16b) in view of Equations (10) and (11) and assuming $e_{0}a$ = ${\left(e_{0}a\right)}_{s}$ = ${\left(e_{0}a\right)}_{b}$, the total nonlocal axial force (i.e., $N_{s,t}^{nl}$) and the total nonlocal flexural moment (i.e., $M_{y,t}^{nl}$) in the moving nanochassis as a function of the deformation fields are obtained:

(17a) $$\begin{array}{c} \begin{array}{c} N_{s,t}^{nl}=N_{s,t}^{l}+{\left(e_{0}a\right)}^{2}\left(\rho_{b}A_{b}+\rho_{s}A_{s}\right)\;\left(\frac{\partial^{3}X}{\partial t^{2}\partial s}+2V_{s}\frac{\partial^{3}X}{\partial t\partial s^{2}}+V_{s}^{2}\frac{\partial^{3}X}{\partial s^{3}}\right), \end{array} \end{array}$$

(17b) $$\begin{array}{c} \begin{array}{c} M_{y,t}^{nl}=M_{y,t}^{l}+{\left(e_{0}a\right)}^{2}\left[\begin{array}{c} \left(\rho_{b}A_{b}+\rho_{s}A_{s}\right)\;\left(\frac{\partial^{2}Y}{\partial t^{2}}+2V_{s}\frac{\partial^{2}Y}{\partial s\partial t}+V_{s}^{2}\;\frac{\partial^{2}Y}{\partial s^{2}}\right)-\left(\rho_{b}I_{b}+\rho_{s}I_{s}\right)\times \;\\ \left(\frac{\partial^{4}Y}{\partial t^{2}\partial s^{2}}+2V_{s}\frac{\partial^{4}Y}{\partial s^{3}\partial t}+V_{s}^{2}\;\frac{\partial^{4}Y}{\partial s^{4}}\right)-\frac{\partial }{\partial s}\;\left(N_{s,t}^{nl}\;\frac{\partial Y}{\partial s}\right) \end{array}\right], \end{array} \end{array}$$

where the total local axial force and the total local bending moment are as follows:

(18a) $$\begin{array}{c} \begin{array}{c} N_{s,t}^{l}=N_{s,s}^{l}+N_{s,b}^{l}=\tau_{s}A_{s}+\left(E_{s}A_{s}+E_{b}A_{b}\right)\;\left\{\frac{\partial X}{\partial s}+\frac{1}{2}\left[{\left(\frac{\partial X}{\partial s}\right)}^{2}+{\left(\frac{\partial Y}{\partial s}\right)}^{2}\right]\right\}, \end{array} \end{array}$$

(18b) $$\begin{array}{c} \begin{array}{c} M_{y,t}^{l}=M_{y,s}^{l}+M_{y,b}^{l}=\left[\begin{array}{c} \left(\frac{2\nu_{b}I_{b}\tau_{s}}{h_{0}}-\left(E_{b}I_{b}+E_{s}I_{s}\right)\right)\frac{\partial^{2}Y}{\partial s^{2}}\;\\ -\frac{2\nu_{b}I_{b}\rho_{s}}{h_{0}}\;\left(\frac{\partial^{2}Y}{\partial t^{2}}+2V_{s}\frac{\partial^{2}Y}{\partial s\partial t}+V_{s}^{2}\;\frac{\partial^{2}Y}{\partial s^{2}}\right) \end{array}\right], \end{array} \end{array}$$

where $E_{s}$ = $\lambda_{s}+2\mu_{s}$ is defined as the elastic modulus of the surface layer. By substituting Equations (17a) and (17b) in order into Equations (16a) and (16b), the governing equations of the moving nanochassis for large deflections in terms of the total local forces by considering the surface energy and nonlocality are obtained:

(19a) $$\begin{array}{c} \begin{array}{c} \left(\rho_{b}A_{b}+\rho_{s}A_{s}\right)\left[\begin{array}{c} \left(\frac{\partial^{2}X}{\partial t^{2}}+2V_{s}\frac{\partial^{2}X}{\partial s\partial t}+V_{s}^{2}\frac{\partial^{2}X}{\partial s^{2}}\right)\;\\ -{\left(e_{0}a\right)}^{2}\left(\frac{\partial^{4}X}{\partial t^{2}\partial s^{2}}+2V_{s}\frac{\partial^{4}X}{\partial s^{3}\partial t}+V_{s}^{2}\frac{\partial^{4}X}{\partial s^{4}}\right) \end{array}\right]-\frac{\partial N_{s,t}^{l}}{\partial s}=0, \end{array} \end{array}$$

(19b) $$\begin{array}{c} \begin{array}{c} \left(\rho_{b}A_{b}+\rho_{s}A_{s}\right)\left[\begin{array}{c} \left(\frac{\partial^{2}Y}{\partial t^{2}}+2V_{s}\frac{\partial^{2}Y}{\partial s\partial t}+V_{s}^{2}\;\frac{\partial^{2}Y}{\partial s^{2}}\right)\;\\ -{\left(e_{0}a\right)}^{2}\left(\frac{\partial^{4}Y}{\partial t^{2}\partial s^{2}}+2V_{s}\frac{\partial^{4}Y}{\partial s^{3}\partial t}+V_{s}^{2}\;\frac{\partial^{4}Y}{\partial s^{4}}\right) \end{array}\right]\;\\ -\left(\rho_{b}I_{b}+\rho_{s}I_{s}\right)\left[\begin{array}{c} \left(\frac{\partial^{4}Y}{\partial t^{2}\partial s^{2}}+2V_{s}\frac{\partial^{4}Y}{\partial s^{3}\partial t}+V_{s}^{2}\;\frac{\partial^{4}Y}{\partial s^{4}}\right)\;\\ -{\left(e_{0}a\right)}^{2}\left(\frac{\partial^{6}Y}{\partial t^{2}\partial s^{4}}+2V_{s}\frac{\partial^{6}Y}{\partial s^{5}\partial t}+V_{s}^{2}\;\frac{\partial^{6}Y}{\partial s^{6}}\right) \end{array}\right]\;\\ -\left[\frac{\partial }{\partial s}\;\left(N_{s,t}^{nl}\frac{\partial Y}{\partial s}\right)-{\left(e_{0}a\right)}^{2}\frac{\partial^{3}}{\partial s^{3}}\;\left(N_{s,t}^{nl}\frac{\partial Y}{\partial s}\right)\right]-\frac{\partial^{2}M_{y,t}^{l}}{\partial s^{2}}=0. \end{array} \end{array}$$

To state Equations (19a) and (19b) in terms of axial and lateral displacements, Equations (18a) and (18b) are introduced to these relations. Additionally, we assume that the following relationship would be reliable for small rotations:

(20) $$\begin{array}{c} \frac{\partial }{\partial s}\left(N_{s,t}^{nl}\frac{\partial Y}{\partial s}\right)-{\left(e_{0}a\right)}^{2}\frac{\partial^{3}}{\partial s^{3}}\;\left(N_{s,t}^{nl}\frac{\partial Y}{\partial s}\right)\approx \;\\ \frac{\partial }{\partial s}\;\left\{\tau_{s}A_{s}+\left(E_{b}A_{b}+E_{s}A_{s}\right)\left\{\frac{\partial X}{\partial s}+\frac{1}{2}\left[{\left(\frac{\partial X}{\partial s}\right)}^{2}+{\left(\frac{\partial Y}{\partial s}\right)}^{2}\right]\right\}\frac{\partial Y}{\partial s}\right\}. \end{array}$$

Finally, the continuum-based governing equations associated with the nonlinear axial and lateral vibrations of the moving nanochassis accounting for both nonlocality and surface effects in terms of deformations would be:

(21a) $$\begin{array}{c} \begin{array}{c} \left(\rho_{b}A_{b}+\rho_{s}A_{s}\right)\left[\begin{array}{c} \left(\frac{\partial^{2}X}{\partial t^{2}}+2V_{s}\frac{\partial^{2}X}{\partial s\partial t}+V_{s}^{2}\frac{\partial^{2}X}{\partial s^{2}}\right)\;\\ -{\left(e_{0}a\right)}^{2}\left(\frac{\partial^{4}X}{\partial t^{2}\partial s^{2}}+2V_{s}\frac{\partial^{4}X}{\partial s^{3}\partial t}+V_{s}^{2}\frac{\partial^{4}X}{\partial s^{4}}\right) \end{array}\right]\;\\ -\left(E_{s}A_{s}+E_{b}A_{b}\right)\;\frac{\partial }{\partial s}\;\left\{\frac{\partial X}{\partial s}+\frac{1}{2}\left[{\left(\frac{\partial X}{\partial s}\right)}^{2}+{\left(\frac{\partial Y}{\partial s}\right)}^{2}\right]\right\}=0, \end{array} \end{array}$$

(21b) $$\begin{array}{c} \begin{array}{c} \left(\rho_{b}A_{b}+\rho_{s}A_{s}\right)\left[\begin{array}{c} \left(\frac{\partial^{2}Y}{\partial t^{2}}+2V_{s}\frac{\partial^{2}Y}{\partial s\partial t}+V_{s}^{2}\;\frac{\partial^{2}Y}{\partial s^{2}}\right)\;\\ -{\left(e_{0}a\right)}^{2}\left(\frac{\partial^{4}Y}{\partial t^{2}\partial s^{2}}+2V_{s}\frac{\partial^{4}Y}{\partial s^{3}\partial t}+V_{s}^{2}\;\frac{\partial^{4}Y}{\partial s^{4}}\right) \end{array}\right]\;\\ -\left(\rho_{b}I_{b}+\rho_{s}I_{s}-\frac{2\nu_{b}I_{b}\rho_{s}}{h_{0}}\right)\left[\begin{array}{c} \left(\frac{\partial^{4}Y}{\partial t^{2}\partial s^{2}}+2V_{s}\frac{\partial^{4}Y}{\partial s^{3}\partial t}+V_{s}^{2}\;\frac{\partial^{4}Y}{\partial s^{4}}\right)\;\\ -{\left(e_{0}a\right)}^{2}\left(\frac{\partial^{6}Y}{\partial t^{2}\partial s^{4}}+2V_{s}\frac{\partial^{6}Y}{\partial s^{5}\partial t}+V_{s}^{2}\;\frac{\partial^{6}Y}{\partial s^{6}}\right) \end{array}\right]\;\\ -\frac{\partial }{\partial s}\;\left\{\left\{\tau_{s}A_{s}+\left(E_{b}A_{b}+E_{s}A_{s}\right)\;\left\{\frac{\partial X}{\partial s}+\frac{1}{2}\left[{\left(\frac{\partial X}{\partial s}\right)}^{2}+{\left(\frac{\partial Y}{\partial s}\right)}^{2}\right]\right\}\right\}\frac{\partial Y}{\partial s}\right\}\;\\ +\left(\left(E_{b}I_{b}+E_{s}I_{s}\right)-\frac{2\nu_{b}I_{b}\tau_{s}}{h_{0}}\right)\frac{\partial^{4}Y}{\partial s^{4}}=0. \end{array} \end{array}$$

Regardingthe initial conditions, it is supposed that the moving nanochassis would be at rest just before initiation of its forced vibrations due to the existing irregularities on the surface. Thereby,

(22) $$\begin{array}{c} X(s,0)=0,\;\;\;Y(s,0)=0,\;\\ \frac{\partial X}{\partial t}\left(s,0\right)=0,\;\;\;\frac{\partial Y}{\partial t}\left(s,0\right)=0. \end{array}$$

For a moving beam-like nanochassis, its ends are assumed to be simple in the lateral direction and fixed-movable in the axial direction. It implies that the following conditions should be enforced:

(23a) $$\begin{array}{c} \begin{array}{c} X\left(0,t\right)=0,\;\;\;N_{s,t}^{nl}\left(l_{b},t\right)=0,\; \end{array} \end{array}$$

(23b) $$\begin{array}{c} \begin{array}{c} Y\left(0,t\right)=W_{g_{1}}\left(t\right),\;\;\;Y\left(l_{b},t\right)=W_{g_{2}}\left(t\right),\;\;\;M_{y,t}^{nl}\left(0,t\right)=0,\;\;\;M_{y,t}^{nl}\left(l_{b},t\right)=0. \end{array} \end{array}$$

To examine nonlinear vibrations of the excited moving nanochassis more systematically, we consider the following dimensionless factors:

(24) $$\begin{array}{c} \eta =\frac{s}{l_{b}},\;\bar{X}=\frac{X}{l_{b}},\;\bar{Y}=\frac{Y}{l_{b}},\;\tau =\frac{1}{l_{b}^{2}}\sqrt{\frac{E_{b}I_{b}}{\rho_{b}A_{b}}}\;t,\;\mu =\frac{e_{0}a}{l_{b}},\;\lambda =\frac{l_{b}}{\sqrt{I_{b}/A_{b}}},\;\beta_{x}=\frac{V_{s}}{\sqrt{E_{b}/\rho_{b}}},\;\;\\ \chi_{0}=\frac{\rho_{s}A_{s}}{\rho_{b}A_{b}},\;\chi_{1}=\frac{E_{s}A_{s}}{E_{b}A_{b}},\;\chi_{2}=\frac{\rho_{s}I_{s}}{\rho_{b}I_{b}}-\frac{2\nu_{b}\rho_{s}}{h_{0}\rho_{b}},\;\chi_{3}=\frac{E_{s}I_{s}}{E_{b}I_{b}}-\frac{2\nu_{b}\tau_{s}}{h_{0}E_{b}},\;\chi_{4}=\frac{\tau_{s}A_{s}l_{b}^{2}}{E_{b}I_{b}},\;\\ \chi_{5}=\frac{2\nu_{b}\rho_{s}}{\rho_{b}h_{0}},\;\bar{\Upsilon }\left[.\right]=\frac{\partial^{2}\left[.\right]}{\partial \tau^{2}}+2\lambda \beta_{x}\;\frac{\partial^{2}\left[.\right]}{\partial \tau \partial \eta }+{\left(\lambda \beta_{x}\right)}^{2}\;\frac{\partial^{2}\left[.\right]}{\partial \eta^{2}},\;\bar{\Xi }\left[.\right]=\left[.\right]-\mu^{2}\;\frac{\partial^{2}\left[.\right]}{\partial \eta^{2}}, \end{array}$$

where $\bar{\Upsilon }\left[.\right]$ and $\bar{\Xi }\left[.\right]$ are the dimensionless operators. By substituting Equation (24) into Equations (21a) and (21b), the dimensionless nonlinear governing equations of an excited moving nanochassis using the nonlocal surface energy-based continuum mechanics are obtainable in the following compacted form:

(25a) $$\begin{array}{c} \begin{array}{c} \left(1+\chi_{0}\right)\;\bar{\Xi }\left\{\bar{\Upsilon }\left\{\bar{X}\right\}\right\}-\lambda^{2}\left(1+\chi_{1}\right)\frac{\partial }{\partial \eta }\;\left\{\frac{\partial \bar{X}}{\partial \eta }+\frac{1}{2}\left[{\left(\frac{\partial \bar{X}}{\partial \eta }\right)}^{2}+{\left(\frac{\partial \bar{Y}}{\partial \eta }\right)}^{2}\right]\right\}=0, \end{array} \end{array}$$

(25b) $$\begin{array}{c} \begin{array}{c} \bar{\Xi }\left\{\bar{\Upsilon }\left\{\left(1+\chi_{0}\right)\bar{Y}-\lambda^{-2}\left(1+\chi_{2}\right)\frac{\partial^{2}\bar{Y}}{\partial \eta^{2}}\right\}\right\}+\left(1+\chi_{3}\right)\frac{\partial^{4}\bar{Y}}{\partial \eta^{4}}\;\\ -\frac{\partial }{\partial \eta }\;\left\{\left\{\chi_{4}+\lambda^{2}\left(1+\chi_{1}\right)\left\{\frac{\partial \bar{X}}{\partial \eta }+\frac{1}{2}\left[{\left(\frac{\partial \bar{X}}{\partial \eta }\right)}^{2}+{\left(\frac{\partial \bar{Y}}{\partial \eta }\right)}^{2}\right]\right\}\right\}\frac{\partial \bar{Y}}{\partial \eta }\right\}=0, \end{array} \end{array}$$

and by virtue of Equations (22) and (23), the dimensionless form of the initial and boundary conditions reads:

(26a) $$\begin{array}{c} \begin{array}{c} \bar{X}\left(\eta ,0\right)=0,\;\bar{Y}\left(\eta ,0\right)=0;\;\;\;\frac{\partial \bar{X}}{\partial \tau }\left(\eta ,0\right)=0,\;\frac{\partial \bar{Y}}{\partial \tau }\left(\eta ,0\right)=0, \end{array} \end{array}$$

(26b) $$\begin{array}{c} \begin{array}{c} \bar{X}\left(0,\tau \right)=0,\;\;\;\bar{Y}\left(0,\tau \right)={\bar{W}}_{g_{1}}\left(\tau \right),\;\;\bar{Y}\left(1,\tau \right)={\bar{W}}_{g_{2}}\left(\tau \right),\;\\ {\bar{N}}_{s,t}^{nl}\left(1,\tau \right)=0,\;\;\;{\bar{M}}_{y,t}^{nl}\left(0,\tau \right)=0,\;\;{\bar{M}}_{y,t}^{nl}\left(1,\tau \right)=0, \end{array} \end{array}$$

where

(27) $$\begin{array}{c} {\bar{W}}_{g_{1}}\left(\tau \right)=\frac{W_{g_{1}}}{l_{b}},\;{\bar{W}}_{g_{2}}\left(\tau \right)=\frac{W_{g_{2}}}{l_{b}},\;{\bar{N}}_{s,t}^{nl}\left(\eta ,\tau \right)=\frac{N_{s,t}^{nl}\left(s,t\right)l_{b}^{2}}{E_{b}I_{b}},\;{\bar{M}}_{y,t}^{nl}\left(\eta ,\tau \right)=\frac{M_{y,t}^{nl}\left(s,t\right)l_{b}}{E_{b}I_{b}}, \end{array}$$

and the dimensionless total axial force (excluding the initially residual one) and the total flexural moments in terms of deformations take the following form:

(28a) $$\begin{array}{c} \begin{array}{c} {\bar{N}}_{s,t}^{nl}=\lambda^{2}\left(1+\chi_{1}\right)\;\left\{\frac{\partial \bar{X}}{\partial \eta }+\frac{1}{2}\left[{\left(\frac{\partial \bar{X}}{\partial \eta }\right)}^{2}+{\left(\frac{\partial \bar{Y}}{\partial \eta }\right)}^{2}\right]\right\}+\mu^{2}\left(1+\chi_{0}\right)\;\bar{\Upsilon }\left\{\frac{\partial \bar{X}}{\partial \eta }\right\}, \end{array} \end{array}$$

(28b) $$\begin{array}{c} \begin{array}{cc} {\bar{M}}_{y,t}^{nl}= & -\left(1+\chi_{3}\right)\frac{\partial^{2}\bar{Y}}{\partial \eta^{2}}-\lambda^{-2}\chi_{5}\bar{\Upsilon }\left\{\bar{Y}\right\}+\mu^{2}\left\{\bar{\Upsilon }\left\{\left(1+\chi_{0}\right)\bar{Y}-\lambda^{-2}\left(1+\chi_{2}+\chi_{5}\right)\frac{\partial^{2}\bar{Y}}{\partial \eta^{2}}\right\}\right.\;\\ & \left.-\lambda^{2}\left(1+\chi_{1}\right)\frac{\partial }{\partial \eta }\;\left\{\left\{\frac{\partial \bar{X}}{\partial \eta }+\frac{1}{2}\left[{\left(\frac{\partial \bar{X}}{\partial \eta }\right)}^{2}+{\left(\frac{\partial \bar{Y}}{\partial \eta }\right)}^{2}\right]\right\}\frac{\partial \bar{Y}}{\partial \eta }\right\}\right\}. \end{array} \end{array}$$

This broader nanoscale perspective can be reinforced by studies on substrate effects, thin-film growth, ultraviolet emitters, nanoporous optical structures, and free-standing device platforms, all of which underline the importance of boundary, surface, and architecture effects in small-scale systems [96,97,98,99]. High-quality film growth via annealing and MOCVD [100] and non-polar ZnO deposition on templates [101] underline the importance of surface/interface engineering in nanoscale dynamics. In addition, self-assembled tungsten oxide nanofibers [102] and flexible TeSeO hybrid photodetectors [103] demonstrate how nanoscale morphology and surface properties govern mechanical and optoelectronic responses. Further, recent works on deep-ultraviolet photonic structures and plasmonic or extraction-enhancement mechanisms further illustrate how structural configuration and surface/interface engineering can decisively modify system behavior at small scales [104,105,106,107]. Such observations are complemented by cooperative scattering designs, biologically relevant nanoparticle–surface interactions, and surface-sensitive functional materials, which collectively strengthen the case for explicitly accounting for surface-related mechanisms in advanced nanoscale modeling [108,109,110].

### 3.2. Development of an Efficient Numerical Approach for Spatial Discretization

Let us decompose the dimensionless displacements of the neutral axis of the excited moving nanochassis modeled by the Rayleigh beam as follows:

(29) $$\begin{array}{c} \bar{X}\left(\eta ,\tau \right)={\bar{X}}_{p}\left(\eta ,\tau \right)+{\bar{X}}_{a}\left(\eta ,\tau \right),\;\\ \bar{Y}\left(\eta ,\tau \right)={\bar{Y}}_{p}\left(\eta ,\tau \right)+{\bar{Y}}_{a}\left(\eta ,\tau \right), \end{array}$$

where, herein, ${\bar{X}}_{p}\left(\eta ,\tau \right)$ and ${\bar{Y}}_{p}\left(\eta ,\tau \right)$ represent the purely dynamic displacements of the neutral axis of the moving beam-like nanochassis along the axial and lateral directions, ${\bar{X}}_{a}$ can be rationally set equal to zero since it is assumed that the moving nanostructure is free from any longitudinal excitation, and ${\bar{Y}}_{a}$ is an auxiliary dimensionless deflection that includes rigid-like transverse displacement of the moving nanostructure due to its supports’ excitation. In the following, we should try to reconstruct the nonlinear equations of motion of the moving nanostructure with excited supports in terms of such auxiliary deformation fields. To this end, let us express the dimensionless auxiliary deflection in the following form:

(30) $$\begin{array}{c} {\bar{Y}}_{a}\left(\eta ,\tau \right)=\left({\bar{W}}_{g_{2}}\left(\tau \right)-{\bar{W}}_{g_{1}}\left(\tau \right)\right)\;\eta +{\bar{W}}_{g_{1}}\left(\tau \right). \end{array}$$

By substituting Equation (29) into Equations (25) and (26) in view of Equation (30), the boundary value problem associated with the nanomechanical problem reduces to the following governing equations:

(31a) $$\begin{array}{c} \begin{array}{c} \left(1+\chi_{0}\right)\;\bar{\Xi }\left\{\bar{\Upsilon }\left\{{\bar{X}}_{p}\right\}\right\}-\lambda^{2}\left(1+\chi_{1}\right)\frac{\partial }{\partial \eta }\;\left\{\frac{\partial {\bar{X}}_{p}}{\partial \eta }+\frac{1}{2}\left[{\left(\frac{\partial {\bar{X}}_{p}}{\partial \eta }\right)}^{2}+{\left(\frac{\partial {\bar{Y}}_{p}}{\partial \eta }\right)}^{2}\right]\right\}=0,\; \end{array} \end{array}$$

(31b) $$\begin{array}{c} \begin{array}{c} \bar{\Xi }\left\{\bar{\Upsilon }\left\{\left(1+\chi_{0}\right){\bar{Y}}_{p}-\lambda^{-2}\left(1+\chi_{2}\right)\frac{\partial^{2}{\bar{Y}}_{p}}{\partial \eta^{2}}\right\}\right\}-\lambda^{2}\left(1+\chi_{1}\right)\left({\bar{W}}_{g_{2}}\left(\tau \right)-{\bar{W}}_{g_{1}}\left(\tau \right)\right)\;\frac{\partial^{2}{\bar{Y}}_{p}}{\partial \eta^{2}}\;\\ -\frac{\partial }{\partial \eta }\;\left\{\left\{\chi_{4}+\lambda^{2}\left(1+\chi_{1}\right)\left\{\frac{\partial {\bar{X}}_{p}}{\partial \eta }+\frac{1}{2}\left[{\left(\frac{\partial {\bar{X}}_{p}}{\partial \eta }\right)}^{2}+{\left(\frac{\partial {\bar{Y}}_{p}}{\partial \eta }\right)}^{2}\right]\right\}\right\}\frac{\partial {\bar{Y}}_{p}}{\partial \eta }\right\}+\left(1+\chi_{3}\right)\frac{\partial^{4}{\bar{Y}}_{p}}{\partial \eta^{4}}\;\\ =-\left(1+\chi_{0}\right)\left\{\left[\left(\frac{d^{2}{\bar{W}}_{g_{2}}}{d\tau^{2}}-\frac{d^{2}{\bar{W}}_{g_{1}}}{d\tau^{2}}\right)\eta +\frac{d^{2}{\bar{W}}_{g_{1}}}{d\tau^{2}}\right]+2\lambda \beta_{x}\;\left(\frac{d{\bar{W}}_{g_{2}}}{d\tau }-\frac{d{\bar{W}}_{g_{1}}}{d\tau }\right)\right\}, \end{array} \end{array}$$

whose initial and boundary conditions read:

(32a) $$\begin{array}{c} \begin{array}{cc} {\bar{X}}_{p}\left(\eta ,0\right)=0, & {\bar{Y}}_{p}\left(\eta ,0\right)=-\left[\left({\bar{W}}_{g_{2}}\left(0\right)-{\bar{W}}_{g_{1}}\left(0\right)\right)\;\eta +{\bar{W}}_{g_{1}}\left(0\right)\right],\;\\ \frac{\partial {\bar{X}}_{p}}{\partial \tau }\left(\eta ,0\right)=0, & \frac{\partial {\bar{Y}}_{p}}{\partial \tau }\left(\eta ,0\right)=-\left[\left(\frac{d{\bar{W}}_{g_{2}}}{d\tau }\left(0\right)-\frac{d{\bar{W}}_{g_{1}}}{d\tau }\left(0\right)\right)\;\eta +\frac{d{\bar{W}}_{g_{1}}}{d\tau }\left(0\right)\right], \end{array} \end{array}$$

(32b) $$\begin{array}{c} \begin{array}{c} {\bar{X}}_{p}\left(0,\tau \right)=0,\;\;\;{\bar{Y}}_{p}\left(0,\tau \right)=0,\;\;{\bar{Y}}_{p}\left(1,\tau \right)=0,\;\\ {\bar{N}}_{s,t}^{nl}\left(1,\tau \right)=0,\;\;\;{\bar{M}}_{y,t}^{nl}\left(0,\tau \right)=0,\;\;{\bar{M}}_{y,t}^{nl}\left(1,\tau \right)=0, \end{array} \end{array}$$

and the dimensionless total axial force and flexural moment in terms of the pure dynamic deformations by consideration of both nonlocality and surface effect take the following form:

(33a) $$\begin{array}{c} \begin{array}{cc} {\bar{N}}_{s,t}^{nl}= & \lambda^{2}\left(1+\chi_{1}\right)\;\left\{\frac{\partial {\bar{X}}_{p}}{\partial \eta }+\frac{1}{2}\left[\begin{array}{c} {\left(\frac{\partial {\bar{X}}_{p}}{\partial \eta }\right)}^{2}+\left(\frac{\partial {\bar{Y}}_{p}}{\partial \eta }+\right.\;\\ {\left.\left({\bar{W}}_{g2}\left(\tau \right)-{\bar{W}}_{g1}\left(\tau \right)\right)\right)}^{2} \end{array}\right]\right\}+\mu^{2}\left(1+\chi_{0}\right)\;\bar{\Upsilon }\left\{\frac{\partial {\bar{X}}_{p}}{\partial \eta }\right\}, \end{array} \end{array}$$

(33b) $$\begin{array}{c} \begin{array}{c} {\bar{M}}_{y,t}^{nl}=-\left(1+\chi_{3}\right)\frac{\partial^{2}{\bar{Y}}_{p}}{\partial \eta^{2}}-\lambda^{-2}\chi_{5}\;\left\{\bar{\Upsilon }\left\{{\bar{Y}}_{p}\right\}+\left(\frac{d^{2}{\bar{W}}_{g_{2}}}{d\tau^{2}}-\frac{d^{2}{\bar{W}}_{g_{1}}}{d\tau^{2}}\right)\eta +\frac{d^{2}{\bar{W}}_{g_{1}}}{d\tau^{2}}\right\}\;\\ +\mu^{2}\left\{\begin{array}{c} \bar{\Upsilon }\left\{\left(1+\chi_{0}\right){\bar{Y}}_{p}-\lambda^{-2}\left(1+\chi_{2}+\chi_{5}\right)\frac{\partial^{2}{\bar{Y}}_{p}}{\partial \eta^{2}}\right\}+\left(1+\chi_{0}\right)\times \;\\ \left[\left(\frac{d^{2}{\bar{W}}_{g_{2}}}{d\tau^{2}}-\frac{d^{2}{\bar{W}}_{g_{1}}}{d\tau^{2}}\right)\eta +\frac{d^{2}{\bar{W}}_{g_{1}}}{d\tau^{2}}\right]-\lambda^{2}\left(1+\chi_{1}\right)\left[\frac{\partial^{2}{\bar{X}}_{p}}{\partial \eta^{2}}\;\left(1+\frac{\partial {\bar{X}}_{p}}{\partial \eta }\right)\times \right.\;\\ \left.\left(\frac{\partial \bar{Y}}{\partial \eta }+\left({\bar{W}}_{g_{2}}\left(\tau \right)-{\bar{W}}_{g_{1}}\left(\tau \right)\right)\right)+{\left(\frac{\partial {\bar{Y}}_{p}}{\partial \eta }+\left({\bar{W}}_{g_{2}}\left(\tau \right)-{\bar{W}}_{g_{1}}\left(\tau \right)\right)\right)}^{2}\;\frac{\partial^{2}{\bar{Y}}_{p}}{\partial \eta^{2}}\right] \end{array}\right\}. \end{array} \end{array}$$

To proceed in solving the nonlinear nonlocal differential-surface energy-based Equations (31a) and (31b) with the given conditions in Equations (32a) and (32b), we employ the Galerkin approach based on the admissible modes. To this end, the dimensionless displacements of the excited moving nanochassis are discretized as a function of admissible mode shapes:

(34) $$\begin{array}{c} {\bar{X}}_{p}\left(\eta ,\tau \right)=\sum_{j=1}^{NM_{X}}\phi_{j}^{X}\left(\eta \right){\bar{X}}_{p_{j}}\left(\tau \right),\;\;\;{\bar{Y}}_{p}\left(\eta ,\tau \right)=\sum_{j=1}^{NM_{Y}}\phi_{j}^{Y}\left(\eta \right){\bar{Y}}_{p_{j}}\left(\tau \right), \end{array}$$

where ${\bar{X}}_{p_{j}}\left(\tau \right)$ and ${\bar{Y}}_{p_{j}}\left(\tau \right)$ denote the time-dependent factors of the *i*th mode of the axial and lateral displacements, respectively, $\phi_{j}^{X}\left(\eta \right)$ and $\phi_{j}^{Y}\left(\eta \right)$ in order are the *j*th modes pertinent to the axial and lateral deformations of the nanochassis’s neutral axis, and finally, $NM_{X}$ and $NM_{Y}$ are the total numbers of modes considered for the nonlinear axial and lateral vibrations, respectively. Now, Equations (31a) and (31b) in order are premultiplied by $\delta {\bar{X}}_{p}$ and $\delta {\bar{Y}}_{p}$ (δ is the variational sign), then the resulting statements are integrated across the length of the moving nanochassis and the integration by parts technique is implemented whenever is required. As a result, the set of ordinary differential equations (ODEs) associated with the nonlinear vibrations of the excited moving nanochassis are extractable as:

(35) $$\begin{array}{c} {\bar{G}}_{b}\;\frac{d^{2}\bar{x}}{d\tau^{2}}={\bar{p}}_{b}, \end{array}$$

or in a more expanded form:

(36) $$\begin{array}{c} \left[\begin{array}{cc} {\bar{G}}_{b}^{uu} & {\bar{G}}_{b}^{uw}\;\\ {\bar{G}}_{b}^{wu} & {\bar{G}}_{b}^{ww} \end{array}\right]\;\left\{\begin{array}{c} \frac{d^{2}\bar{x}}{d\tau^{2}}\;\\ \frac{d^{2}\bar{y}}{d\tau^{2}} \end{array}\right\}=\left\{\begin{array}{c} {\bar{p}}_{b}^{X}\;\\ {\bar{p}}_{b}^{Y} \end{array}\right\}, \end{array}$$

in which the values of ${\bar{G}}_{b}^{\alpha \beta }$ and ${\bar{p}}_{b}^{\gamma }$ are provided in Appendix B.1. Now, through employing Equation (32a), the initial conditions of the time-dependent vectors of deformation fields are calculated by:

(37a) $$\begin{array}{c} \begin{array}{c} \bar{x}\left(0\right)=0,\;\;\;\bar{y}\left(0\right)=-A\;Y_{0}, \end{array} \end{array}$$

(37b) $$\begin{array}{c} \begin{array}{c} \frac{d\bar{x}}{d\tau }\left(0\right)=0,\;\;\;\frac{d\bar{y}}{d\tau }\left(0\right)=-A\;{\dot{Y}}_{0}, \end{array} \end{array}$$

where

(38) $$\begin{array}{c} A_{ij}=\int_{0}^{1}\phi_{i}^{Y}\phi_{j}^{Y}\;d\eta ,\;\\ {\left\{Y_{0}\right\}}_{i}=\int_{0}^{1}\phi_{i}^{Y}\;\left(\left({\bar{W}}_{g_{2}}\left(0\right)-{\bar{W}}_{g_{1}}\left(0\right)\right)\;\eta +{\bar{W}}_{g_{1}}\left(0\right)\right)d\eta ,\;\\ {\left\{{\dot{Y}}_{0}\right\}}_{i}=\int_{0}^{1}\phi_{i}^{Y}\;\left(\left(\frac{d{\bar{W}}_{g_{2}}}{d\tau }\left(0\right)-\frac{d{\bar{W}}_{g_{1}}}{d\tau }\left(0\right)\right)\;\eta +\frac{d{\bar{W}}_{g_{1}}}{d\tau }\left(0\right)\right)d\eta . \end{array}$$

### 3.3. Application of the Newton’s Approach to the Nonlinear Equations

To calculate the time-dependent vector in Equation (36), let us write down: $\bar{y}=\frac{d\bar{x}}{d\tau }$. Therefore, Equation (35) can also be modified to:

(39) $$\begin{array}{c} \hat{G}\;\frac{d\bar{q}}{d\tau }=\hat{p}, \end{array}$$

where

(40) $$\begin{array}{c} \bar{q}=\left\{\begin{array}{c} \bar{y}\\ \bar{x} \end{array}\right\},\; \end{array}\hat{G}=\left[\begin{array}{cc} {\bar{G}}_{b} & \;\;\;0\\ 0 & \;\;\;I \end{array}\right],\;\begin{array}{c} \hat{p}=\left\{\begin{array}{c} {\bar{p}}_{b}\\ \bar{y} \end{array}\right\} \end{array}$$

in which **I** represents the identity matrix. Using finite difference approach, Equation (39) can be discretized in the time domain. To this end, let us approximate the first derivative of $\bar{q}$ by: $\frac{d\bar{q}}{d\tau }\approx \frac{{\bar{q}}_{i+1}-{\bar{q}}_{i}}{▵\tau_{i}}$, where ${\bar{q}}_{i}=\bar{q}\left(\tau_{i}\right)$, ${\bar{q}}_{i+1}=\bar{q}\left(\tau_{i+1}\right)$, and $▵\tau_{i}=\tau_{i+1}-\tau_{i}$. Through introducing the recent approximate relation to Equation (39), it is obtainable:

(41) $$\begin{array}{c} \tilde{G}{\bar{q}}_{i+1}-\tilde{G}{\bar{q}}_{i}-▵\tau \;\tilde{p}=0,\\ \tilde{G}=\left(1-\zeta \right)\;{\hat{G}}_{i}+\zeta \;{\hat{G}}_{i+1}\;,\\ \tilde{p}=\left(1-\zeta \right)\;{\hat{p}}_{i}+\zeta \;{\hat{p}}_{i+1}\;, \end{array}$$

where ζ denotes the weight parameter of the time discretization. By utilizing the Newton approach, the value of ${\bar{q}}_{i+1}$ can be computed from the nonlinear set of equations in Equation (41) as follows:

(42) $$\begin{array}{c} \overset{ˇ}{K}\;▵{\bar{q}}_{i+1}=\overset{ˇ}{p},\\ \overset{ˇ}{K}=\tilde{G}-▵\tau \;\zeta \;{\left(\frac{\partial \tilde{p}}{\partial \bar{q}}\right)}_{\bar{q}={\bar{q}}_{i+1}^{old}},\\ \overset{ˇ}{p}=-\tilde{G}\;{\bar{q}}_{i+1}^{old}+\tilde{G}\;{\bar{q}}_{i}+▵\tau \;\tilde{p},\\ ▵{\bar{q}}_{i+1}={\bar{q}}_{i+1}^{\;new}-{\bar{q}}_{i+1}^{\;old}, \end{array}$$

where ${\bar{q}}_{i+1}^{new}$ and ${\bar{q}}_{i+1}^{old}$ denote the new and old values of ${\bar{q}}_{i+1}$ during the iteration process, and the details of the calculation of $\frac{\partial \tilde{p}}{\partial \bar{q}}$ have been presented in the Supplementary Materials, Section S.1. By carrying out the iteration trend given in Equation (42), fairly accurate values for ${\bar{q}}_{i+1}^{new}$ can be achieved at each time.

## 4. Nonlinear Nonlocal Integro-Based Modeling of Moving Excited Nanochassis

### 4.1. Nonlinear Equations of Motion

Based on the nonlocal integro-based version of the continuum mechanics of Eringen [24], the longitudinal stresses within the surface layer and the bulk can be linked to their corresponding local stresses by:

(43a) $$\begin{array}{c} \begin{array}{c} \sigma_{ss,s}^{nl}\left(s,y,t\right)=\int \int \int_{\Omega_{s}}\alpha_{s}(|s-s^{\prime }\left|;l_{s}\right)\;\sigma_{ss,s}^{l}\left(s^{\prime },y,t\right)\;d\Omega_{s}^{\prime },\; \end{array} \end{array}$$

(43b) $$\begin{array}{c} \begin{array}{c} \sigma_{ss,b}^{nl}\left(s,y,t\right)=\int \int \int_{\Omega_{b}}\alpha_{b}(|s-s^{\prime }\left|;l_{s}\right)\;\sigma_{ss,b}^{l}\left(s^{\prime },y,t\right)\;d\Omega_{b}^{\prime },\; \end{array} \end{array}$$

where $\Omega_{s}$ denotes the surface layer’s domain, $\Omega_{b}$ is the spatial domain of the bulk, $d\Omega_{s}$ and $d\Omega_{b}$ are their corresponding infinitesimal elements, $\alpha_{s}(|s-s^{\prime }\left|;l_{s}\right)$ and $\alpha_{b}(|s-s^{\prime }\left|;l_{s}\right)$ represent suitable one-dimensional kernel functions for the constituting parts of the continuum-based nanochassis, and $l_{s}$ is the small-scale factor (i.e., $l_{s}$ = $e_{0}a$), which is assumed to be the same in the constituents of the moving nanochassis. The requirement of the completeness condition for the kernel function leads to: $\int \int \int_{\Omega_{s}}\alpha_{s}(|s-s^{\prime }\left|;l_{s}\right)$ = 1 and $\int \int \int_{\Omega_{b}}\alpha_{b}(|s-s^{\prime }\left|;l_{s}\right)$ = 1. Now by considering the following relations:

(44) $$\begin{array}{c} \alpha_{s}(|s-s^{\prime }|;l_{s})=\alpha_{s0}\;F(|s-s^{\prime }|;l_{s}),\;\;\;\alpha_{b}(|s-s^{\prime }|;l_{s})=\alpha_{b0}\;F(|s-s^{\prime }\left|;l_{s}\right), \end{array}$$

where $F(|s-s^{\prime }|;l_{s})$ is an appropriate attenuation function, and the above-mentioned constants pertinent to these kernel functions are defined by:

(45) $$\begin{array}{c} \alpha_{s0}=\frac{\alpha_{0}}{A_{s}},\;\alpha_{b0}=\frac{\alpha_{0}}{A_{b}};\;\alpha_{0}=\frac{1}{\;\int_{0}^{l_{b}}F\left(s;l_{s}\right)\;ds}, \end{array}$$

and commonly used one-dimensional attenuation functions are as [24]:

(46) $$\begin{array}{c} F\left(s;l_{s}\right)=\exp\left(-\frac{s}{l_{s}}\right),\;\exp\left(-{\left(k\frac{s}{l_{s}}\right)}^{2}\right);\;k=1.65,\;\left(1-\frac{s}{l_{s}}\right)\;H\left(1-\frac{s}{e_{0}a}\right), \end{array}$$

where H is the Heaviside step function. By introducing Equation (44) to Equation (43) by virtue of Equation (45), the nonlocal main normal stresses within the consisting parts of the moving nanochassis are expressed in terms of their pertinent local values as follows:

(47a) $$\begin{array}{c} \begin{array}{c} \sigma_{ss,s}^{nl}\left(s,y,t\right)=\int_{0}^{l_{b}}\alpha_{0}\;F(|s-s^{\prime }\left|;l_{s}\right)\;\sigma_{ss,s}^{l}\left(s^{\prime },y,t\right)\;ds^{\prime },\; \end{array} \end{array}$$

(47b) $$\begin{array}{c} \begin{array}{c} \sigma_{ss,b}^{nl}\left(s,y,t\right)=\int_{0}^{l_{b}}\alpha_{0}\;F(|s-s^{\prime }\left|;l_{s}\right)\;\sigma_{ss,b}^{l}\left(s^{\prime },y,t\right)\;ds^{\prime }.\; \end{array} \end{array}$$

By introducing the local stresses, as given in Equations (7) and (9), to Equations (47a) and (47b), the nonlocal integral-based stresses within the constituent parts of the moving nanochassis in terms of displacements are displayed by:

(48a) $$\begin{array}{c} \begin{array}{cc} \sigma_{ss,s}^{nl}\left(s,y,t\right)= & \tau_{s}+\left(\lambda_{s}+2\mu_{s}\right)\;\int_{0}^{l_{b}}\alpha_{0}\;F(|s-s^{\prime }\left|;l_{s}\right)\;\left\{\frac{\partial X}{\partial s}+\frac{1}{2}\left[{\left(\frac{\partial X}{\partial s}\right)}^{2}+{\left(\frac{\partial Y}{\partial s}\right)}^{2}\right]\right\}\;ds^{\prime }\;\\ & -y\;\left(\lambda_{s}+2\mu_{s}\right)\;\int_{0}^{l_{b}}\alpha_{0}\;F(|s-s^{\prime }\left|;l_{s}\right)\;\frac{\partial^{2}Y}{\partial s^{2}}\;ds^{\prime }, \end{array} \end{array}$$

(48b) $$\begin{array}{c} \begin{array}{cc} \sigma_{ss,b}^{nl}\left(s,y,t\right)= & E_{b}\;\int_{0}^{l_{b}}\alpha_{0}\;F(|s-s^{\prime }\left|;l_{s}\right)\;\left\{\frac{\partial X}{\partial s}+\frac{1}{2}\left[{\left(\frac{\partial X}{\partial s}\right)}^{2}+{\left(\frac{\partial Y}{\partial s}\right)}^{2}\right]\right\}\;ds^{\prime }\;\\ & +y\;\int_{0}^{l_{b}}\alpha_{0}\;F(|s-s^{\prime }\left|;l_{s}\right)\;\left[\begin{array}{c} \left(\frac{2\nu_{b}\tau_{s}}{h_{0}}-E_{b}\right)\frac{\partial^{2}Y}{\partial s^{2}}-\frac{2\nu_{b}\rho_{s}}{h_{0}}\times \;\\ \left(\frac{\partial^{2}Y}{\partial t^{2}}+2V_{s}\frac{\partial^{2}Y}{\partial s\partial t}+V_{s}^{2}\;\frac{\partial^{2}Y}{\partial s^{2}}\right) \end{array}\right]\;ds^{\prime }. \end{array} \end{array}$$

Therefore, the total nonlocal axial force field in the consisting parts of the beam-like nanochassis are expressed by:

(49a) $$\begin{array}{c} \begin{array}{c} N_{s,s}^{nl}\left(s,t\right)=\tau_{s}A_{s}+\left(\lambda_{s}+2\mu_{s}\right)A_{s}\;\int_{0}^{l_{b}}\alpha_{0}\;F(|s-s^{\prime }\left|;l_{s}\right)\;\left\{\frac{\partial X}{\partial s}+\frac{1}{2}\left[{\left(\frac{\partial X}{\partial s}\right)}^{2}+{\left(\frac{\partial Y}{\partial s}\right)}^{2}\right]\right\}\;ds^{\prime },\; \end{array} \end{array}$$

(49b) $$\begin{array}{c} \begin{array}{c} N_{s,b}^{nl}\left(s,t\right)=E_{b}A_{b}\;\int_{0}^{l_{b}}\alpha_{0}\;F(|s-s^{\prime }\left|;l_{s}\right)\;\left\{\frac{\partial X}{\partial s}+\frac{1}{2}\left[{\left(\frac{\partial X}{\partial s}\right)}^{2}+{\left(\frac{\partial Y}{\partial s}\right)}^{2}\right]\right\}\;ds^{\prime }, \end{array} \end{array}$$

and the total nonlocal flexural moments in the constituting parts of the movingly excited nanostructure are readily stated by:

(50a) $$\begin{array}{c} \begin{array}{c} M_{y,s}^{nl}\left(s,t\right)=-I_{s}\;\left(\lambda_{s}+2\mu_{s}\right)\;\int_{0}^{l_{b}}\alpha_{0}\;F(|s-s^{\prime }\left|;l_{s}\right)\;\frac{\partial^{2}Y}{\partial s^{2}}\;ds^{\prime }, \end{array} \end{array}$$

(50b) $$\begin{array}{c} \begin{array}{c} M_{y,b}^{nl}\left(s,t\right)=I_{b}\;\int_{0}^{l_{b}}\alpha_{0}\;F(|s-s^{\prime }\left|;l_{s}\right)\;\left[\begin{array}{c} \left(\frac{2\nu_{b}\tau_{s}}{h_{0}}-E_{b}\right)\frac{\partial^{2}Y}{\partial s^{2}}-\frac{2\nu_{b}\rho_{s}}{h_{0}}\times \;\\ \left(\frac{\partial^{2}Y}{\partial t^{2}}+2V_{s}\frac{\partial^{2}Y}{\partial s\partial t}+V_{s}^{2}\;\frac{\partial^{2}Y}{\partial s^{2}}\right) \end{array}\right]\;ds^{\prime }. \end{array} \end{array}$$

Using the principle of Hamilton, in view of Equations (15a) and (15b), the nonlocal integro-surface energy-based equations associated with the axial and lateral vibrations of the moving nanochassis take the following form:

(51a) $$\begin{array}{c} \begin{array}{c} \left(\rho_{b}A_{b}+\rho_{s}A_{s}\right)\;\left(\frac{\partial^{2}X}{\partial t^{2}}+2V_{s}\frac{\partial^{2}X}{\partial s\partial t}+V_{s}^{2}\frac{\partial^{2}X}{\partial s^{2}}\right)-\frac{\partial }{\partial s}\left[\left(\left(\lambda_{s}+2\mu_{s}\right)A_{s}+E_{b}A_{b}\right)\times \right.\;\\ \left.\int_{0}^{l_{b}}\alpha_{0}\;F(|s-s^{\prime }\left|;l_{s}\right)\;\left\{\frac{\partial X}{\partial s}+\frac{1}{2}\left[{\left(\frac{\partial X}{\partial s}\right)}^{2}+{\left(\frac{\partial Y}{\partial s}\right)}^{2}\right]\right\}\;ds^{\prime }\right]=0, \end{array} \end{array}$$

(51b) $$\begin{array}{c} \begin{array}{c} \left(\rho_{b}A_{b}+\rho_{s}A_{s}\right)\;\left(\frac{\partial^{2}Y}{\partial t^{2}}+2V_{s}\frac{\partial^{2}Y}{\partial s\partial t}+V_{s}^{2}\;\frac{\partial^{2}Y}{\partial s^{2}}\right)-\left(\rho_{b}I_{b}+\rho_{s}I_{s}\right)\left(\frac{\partial^{4}Y}{\partial t^{2}\partial s^{2}}+2V_{s}\frac{\partial^{4}Y}{\partial s^{3}\partial t}\right.\;\\ \left.+V_{s}^{2}\;\frac{\partial^{4}Y}{\partial s^{4}}\right)-\frac{\partial }{\partial s}\;\left\{\left[\left(\left(\lambda_{s}+2\mu_{s}\right)A_{s}+E_{b}A_{b}\right)\int_{0}^{l_{b}}\alpha_{0}\;F(|s-s^{\prime }\left|;l_{s}\right)\times \right.\right.\;\\ \left.\left.\left\{\frac{\partial X}{\partial s}+\frac{1}{2}\left[{\left(\frac{\partial X}{\partial s}\right)}^{2}+{\left(\frac{\partial Y}{\partial s}\right)}^{2}\right]\right\}\;ds^{\prime }\right]\frac{\partial Y}{\partial s}\right\}\;\\ -\frac{\partial^{2}}{\partial s^{2}}\left\{\int_{0}^{l_{b}}\alpha_{0}\;F(|s-s^{\prime }\left|;l_{s}\right)\;\left[\begin{array}{c} \left(E_{b}I_{b}+\left(\lambda_{s}+2\mu_{s}\right)I_{s}-\frac{2\nu_{b}I_{b}\tau_{s}}{h_{0}}\right)\frac{\partial^{2}Y}{\partial s^{2}}\;\\ +\frac{2\nu_{b}I_{b}\rho_{s}}{h_{0}}\;\left(\frac{\partial^{2}Y}{\partial t^{2}}+2V_{s}\frac{\partial^{2}Y}{\partial s\partial t}+V_{s}^{2}\;\frac{\partial^{2}Y}{\partial s^{2}}\right) \end{array}\right]\;ds^{\prime }\right\}=0, \end{array} \end{array}$$

with the given initial and boundary conditions in Equations (22) and (23).

Now, the provided dimensionless parameters in Equation (24) are introduced to Equations (51a) and (51b). As a result, the dimensionless nonlinear equations of axial and lateral vibrations of the beam-like excited moving nanochassis are obtained:

(52a) $$\begin{array}{c} \begin{array}{c} \left(1+\chi_{0}\right)\;\bar{\Upsilon }\left\{\bar{X}\right\}-\lambda^{2}\left(1+\chi_{1}\right)\;\frac{\partial }{\partial \eta }\;\left\{\int_{0}^{1}{\bar{\alpha }}_{0}\;F(|\eta -\eta^{\prime }\left|;{\bar{l}}_{s}\right)\;\right.\;\\ \left.\left\{\frac{\partial \bar{X}}{\partial \eta }+\frac{1}{2}\left[{\left(\frac{\partial \bar{X}}{\partial \eta }\right)}^{2}+{\left(\frac{\partial \bar{Y}}{\partial \eta }\right)}^{2}\right]\right\}d\eta^{\prime }\right\}=0,\; \end{array} \end{array}$$

(52b) $$\begin{array}{c} \begin{array}{c} \bar{\Upsilon }\left\{\left(1+\chi_{0}\right)\bar{Y}-\lambda^{-2}\left(1+\chi_{21}\right)\frac{\partial^{2}\bar{Y}}{\partial \eta^{2}}\right\}-\chi_{4}\;\frac{\partial^{2}\bar{Y}}{\partial \eta^{2}}-\lambda^{2}\left(1+\chi_{1}\right)\times \;\\ \frac{\partial }{\partial \eta }\;\left\{\frac{\partial \bar{Y}}{\partial \eta }\;\int_{0}^{1}{\bar{\alpha }}_{0}\;F(|\eta -\eta^{\prime }\left|;{\bar{l}}_{s}\right)\;\left\{\frac{\partial \bar{X}}{\partial \eta }+\frac{1}{2}\left[{\left(\frac{\partial \bar{X}}{\partial \eta }\right)}^{2}+{\left(\frac{\partial \bar{Y}}{\partial \eta }\right)}^{2}\right]\right\}d\eta^{\prime }\right\}\;\\ +\frac{\partial^{2}}{\partial \eta^{2}}\;\left[\int_{0}^{1}{\bar{\alpha }}_{0}\;F(|\eta -\eta^{\prime }\left|;{\bar{l}}_{s}\right)\;\left(\left(1+\chi_{3}\right)\frac{\partial^{2}\bar{Y}}{\partial \eta^{2}}+\chi_{22}\bar{\Upsilon }\left\{\bar{Y}\right\}\right)d\eta^{\prime }\right]=0,\; \end{array} \end{array}$$

with the dimensionless conditions provided in Equations (26a) and (26b), and

(53) $$\begin{array}{c} {\bar{\alpha }}_{0}=\alpha_{0}\;l_{b},\;\chi_{21}=\frac{\rho_{s}I_{s}}{\rho_{b}I_{b}},\;\chi_{22}=\frac{2\nu_{b}\rho_{s}}{h_{0}\rho_{b}}. \end{array}$$

### 4.2. Development of an Efficient Numerical Approach for Spatial Discretization

Assuming the decomposed form of the dimensionless deformation fields of the neutral axis of the continuum-based moving nanochassis as per Equations (29) through using Equation (30), Equations (52a) and (52b) can be rewritten in terms of ${\bar{X}}_{p}$ and ${\bar{Y}}_{p}$ as follows:

(54a) $$\begin{array}{c} \begin{array}{c} \left(1+\chi_{0}\right)\;\bar{\Upsilon }\left\{{\bar{X}}_{p}\right\}-\lambda^{2}\left(1+\chi_{1}\right)\;\frac{\partial }{\partial \eta }\;\left\{\int_{0}^{1}{\bar{\alpha }}_{0}\;F(|\eta -\eta^{\prime }\left|;{\bar{l}}_{s}\right)\;\left\{\left[\frac{\partial {\bar{X}}_{p}}{\partial \eta }+\frac{\partial {\bar{Y}}_{p}}{\partial \eta }\left({\bar{W}}_{g_{2}}-{\bar{W}}_{g_{1}}\right)\right]+\right.\right.\;\\ \left.\left.\frac{1}{2}\left[{\left(\frac{\partial {\bar{X}}_{p}}{\partial \eta }\right)}^{2}+{\left(\frac{\partial {\bar{Y}}_{p}}{\partial \eta }\right)}^{2}\right]\right\}d\eta^{\prime }\right\}=\frac{\lambda^{2}\left(1+\chi_{1}\right)}{2}\;\frac{\partial }{\partial \eta }\left[\int_{0}^{1}{\bar{\alpha }}_{0}\;F(|\eta -\eta^{\prime }\left|;{\bar{l}}_{s}\right)\;{\left({\bar{W}}_{g_{2}}-{\bar{W}}_{g_{1}}\right)}^{2}d\eta^{\prime }\right],\; \end{array} \end{array}$$

(54b) $$\begin{array}{c} \begin{array}{c} \bar{\Upsilon }\left\{\left(1+\chi_{0}\right){\bar{Y}}_{p}-\lambda^{-2}\left(1+\chi_{21}\right)\frac{\partial^{2}{\bar{Y}}_{p}}{\partial \eta^{2}}\right\}-\chi_{4}\frac{\partial^{2}{\bar{Y}}_{p}}{\partial \eta^{2}}-\lambda^{2}\left(1+\chi_{1}\right)\;\frac{\partial }{\partial \eta }\left\{\left\{\int_{0}^{1}{\bar{\alpha }}_{0}\;F(|\eta -\eta^{\prime }\left|;{\bar{l}}_{s}\right)\right.\right.\;\\ \left.\left.\left\{\left[\frac{\partial {\bar{X}}_{p}}{\partial \eta }+\frac{\partial {\bar{Y}}_{p}}{\partial \eta }\left({\bar{W}}_{g_{2}}-{\bar{W}}_{g_{1}}\right)\right]+\frac{1}{2}\left[{\left(\frac{\partial {\bar{X}}_{p}}{\partial \eta }\right)}^{2}+{\left(\frac{\partial {\bar{Y}}_{p}}{\partial \eta }\right)}^{2}\right]\right\}\right\}d\eta^{\prime }\;\left[\frac{\partial {\bar{Y}}_{p}}{\partial \eta }+\left({\bar{W}}_{g_{2}}\left(\tau \right)-{\bar{W}}_{g_{1}}\left(\tau \right)\right)\right]\right\}\;\\ +\frac{\partial^{2}}{\partial \eta^{2}}\;\left[\int_{0}^{1}{\bar{\alpha }}_{0}\;F(|\eta -\eta^{\prime }\left|;{\bar{l}}_{s}\right)\;\left(\left(1+\chi_{3}\right)\frac{\partial^{2}{\bar{Y}}_{p}}{\partial \eta^{2}}+\chi_{22}\bar{\Upsilon }\left\{{\bar{Y}}_{p}\right\}\right)d\eta^{\prime }\right]\;\\ =-\left(1+\chi_{0}\right)\left\{\left[\left(\frac{d^{2}{\bar{W}}_{g_{2}}}{d\tau^{2}}-\frac{d^{2}{\bar{W}}_{g_{1}}}{d\tau^{2}}\right)\eta +\frac{d^{2}{\bar{W}}_{g_{1}}}{d\tau^{2}}\right]+2\lambda \beta_{x}\;\left(\frac{d{\bar{W}}_{g_{2}}}{d\tau }-\frac{d{\bar{W}}_{g_{1}}}{d\tau }\right)\right\}\;\\ \;\;-\chi_{22}\;\frac{\partial^{2}}{\partial \eta^{2}}\;\left\{\int_{0}^{1}{\bar{\alpha }}_{0}\;F(|\eta -\eta^{\prime }\left|;{\bar{l}}_{s}\right)\;\left[\left(\frac{d^{2}{\bar{W}}_{g_{2}}}{d\tau^{2}}-\frac{d^{2}{\bar{W}}_{g_{1}}}{d\tau^{2}}\right)\eta +\frac{d^{2}{\bar{W}}_{g_{1}}}{d\tau^{2}}\right]d\eta^{\prime }\right\}, \end{array} \end{array}$$

with the given initial and boundary conditions in Equations (32a) and (32b). Since for most of the cases the kernel functions have anti-symmetric slope w.r.t. its symmetric plane, the last term on the right-hand side of Equation (54b) could be simplified to:

(55) $$\begin{array}{c} -\chi_{22}\;\int_{0}^{1}{\bar{\alpha }}_{0}\;\frac{\partial^{2}}{\partial \eta^{2}}\;F(|\eta -\eta^{\prime }\left|;{\bar{l}}_{s}\right)\;\left[\left(\frac{d^{2}{\bar{W}}_{g_{2}}}{d\tau^{2}}-\frac{d^{2}{\bar{W}}_{g_{1}}}{d\tau^{2}}\right)\eta +\frac{d^{2}{\bar{W}}_{g_{1}}}{d\tau^{2}}\right]d\eta^{\prime }. \end{array}$$

Further, the dimensionless axial force and flexural moment based on the nonlocal integral model take the following form:

(56a) $$\begin{array}{c} \begin{array}{c} {\bar{N}}_{s,t}^{nl}=\int_{0}^{1}{\bar{\alpha }}_{0}\;F(|\eta -\eta^{\prime }\left|;{\bar{l}}_{s}\right)\;\left(1+\chi_{1}\right)\;\left\{\begin{array}{c} \left[\frac{\partial {\bar{X}}_{p}}{\partial \eta }+\frac{\partial {\bar{Y}}_{p}}{\partial \eta }\left({\bar{W}}_{g_{2}}-{\bar{W}}_{g_{1}}\right)\right]\;\\ +\frac{1}{2}\left[{\left(\frac{\partial {\bar{X}}_{p}}{\partial \eta }\right)}^{2}+{\left(\frac{\partial {\bar{Y}}_{p}}{\partial \eta }\right)}^{2}\right]\;\\ +\frac{1}{2}{\left({\bar{W}}_{g_{2}}-{\bar{W}}_{g_{1}}\right)}^{2} \end{array}\right\}d\eta^{\prime }, \end{array} \end{array}$$

(56b) $$\begin{array}{c} \begin{array}{c} {\bar{M}}_{y,t}^{nl}=\int_{0}^{1}{\bar{\alpha }}_{0}\;F(|\eta -\eta^{\prime }\left|;{\bar{l}}_{s}\right)\;\left\{\begin{array}{c} \left(1+\chi_{3}\right)\frac{\partial^{2}{\bar{Y}}_{p}}{\partial \eta^{2}}\;\\ +\chi_{22}\left(\frac{\partial^{2}{\bar{Y}}_{p}}{\partial \tau^{2}}+2\lambda \beta_{x}\frac{\partial^{2}{\bar{Y}}_{p}}{\partial \tau \partial \eta }+{\left(\lambda \beta_{x}\right)}^{2}\frac{\partial^{2}{\bar{Y}}_{p}}{\partial \eta^{2}}\right)\;\\ -\chi_{22}\;\left[\left(\frac{d^{2}{\bar{W}}_{g_{2}}}{d\tau^{2}}-\frac{d^{2}{\bar{W}}_{g_{1}}}{d\tau^{2}}\right)\eta^{\prime }+\frac{d^{2}{\bar{W}}_{g_{1}}}{d\tau^{2}}\right] \end{array}\right\}d\eta^{\prime }.\; \end{array} \end{array}$$

By following the displayed procedure in Section 3.2 for discretizing the unknown fields and transferring from PDEs to ODEs, one can arrive at the ODEs given in Equations (35) and (36), where the elements of its consisting submatrices and force vectors are given in Appendix B.2.

### 4.3. Application of the Newton’s Approach to the Nonlinear Equations

By exploiting the given numerical procedure in Section 3.3 and using Newton’s approach, the unknown time-dependent vectors of the dimensionless deformation fields could be readily evaluated. The details of calculations of the elements of $\frac{\partial \tilde{p}}{\partial \bar{q}}$ for this model are also given in Section S.2 of the Supplementary Materials.

## 5. Results and Discussion

### 5.1. Several Comparison Studies

Since the explored problem herein has not been cultivated with such a generality described in the present work, the provided verification studies are restricted to several particular cases based on the established analytical-based approaches in the present work. As the first comparison study, the predicted nonlocal elastic fields by the proposed numerical-based models in the linear condition are verified with those of analytical-based solution for the case of a stationary nanochassis acted upon by end excitation. In the second verification scrutiny, the predicted linear frequencies of the axially moving nanochassis are compared with those of analytical solutions.

#### 5.1.1. Verification of the Elastic Fields Within the Excited Nanochassis

**Figure 2:**
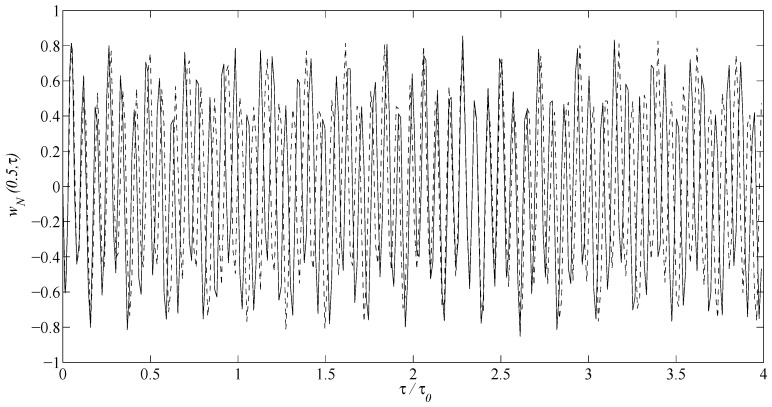
The obtained time history plots of the linear-normalized deflection at the midspan point of the excited nanochassis by the exact solution (solid line) and the suggested numerical NDS-based model (dashed line) (λ = 30, $e_{0}a$ = 2.24 nm, ${\bar{a}}_{i}$ = 0.01, $\bar{\varpi_{i}}$ = 0.3$\varpi_{1}$, $\phi_{i}$ = 0).

To verify some parts of the performed calculations, we also establish an analytically linear model for predicting nonlocal surface energy-based elastic fields within the nanochassis due to ends’ excitations. Consider a simply supported nanochassis in the stationary state (i.e., $V_{s}$ = 0) which is subjected to arbitrary harmonic excitations at its ends. In Section S.3 of the Supplementary Materials, based on the connection of admissible modes and Fourier sine series properties, an exact methodology has been established to display the deflection and flexural moment of the arbitrarily excited nanochassis. Subsequently, for the special case of the nanochassis subjected to harmonically excited ends (i.e., ${\bar{Y}}_{gi}\left(\tau \right)={\bar{a}}_{i}\;\sin\left({\bar{\varpi }}_{i}\tau +\phi_{i}\right)$), the explicit-exact expressions of the purely dynamic deflection and the flexural moment are derived carefully in Sections S.3.1 and S.3.2 of the Supplementary Materials. Further, the resonance state of the excited nanochassis is discussed in Section S.3.3 of the Supplementary Materials. In Figure 2, the predicted linear dynamic deflection by the proposed NDS-based model (dashed line) and those of the presented exact solution (solid line) are provided for various excited frequencies of the ends such that:

(57) $$\begin{array}{c} w_{N}=\frac{\bar{Y}}{{\bar{Y}}_{st,max}},\;\;\tau_{0}=\max\left\{\frac{2\pi }{\varpi_{1}},\frac{1}{\beta_{cr}}\right\},\;\\ \beta_{cr}=\frac{1}{\lambda }\;\sqrt{\frac{\left(1+\chi_{3}\right)\pi^{2}+\chi_{4}}{\left(1+{\left(\mu \pi \right)}^{2}\right)\left(\left(1+\chi_{0}\right)+\left(1+\chi_{2}\right){\left(\pi /\lambda \right)}^{2}\right)}}, \end{array}$$

where $w_{N}$ is the normalized deflection, ${\bar{Y}}_{st,max}$ is the maximum static deflection due to the mass-weight of the nanochassis with simple ends, $\varpi_{1}$ is the dimensionless fundamental frequency of the nanochassis, and $\beta_{cr}$ denotes the dimensionless divergence velocity of the first mode [85]. As it is observable in Figure 2, there exists a reasonably good agreement between the predicted results by the suggested numerical model and those of the exact solution most of the time.

In order to suppress the induced vibrations within the moving nanochassis, its vibrations can be suitably controlled by an appropriate active or passive approach. For this purpose, the utilized methodologies for large-scale structures acted upon by forced vibrations [111,112,113] can be employed. In the present investigation, no internal damping nor an active or a passive control solution is adopted to suppress the induced vibrations, and this crucial issue can be considered as a hot topic for future work.

#### 5.1.2. Verification of the Natural Frequencies of the Surface Energy-Based Moving Nanochassis

**Figure 3:**
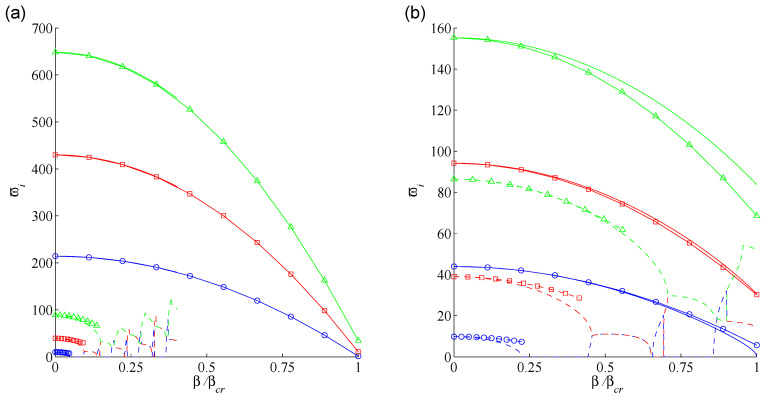
Comparison between the predicted first three dimensionless flexural frequencies by the proposed linear NDS-based model and those of the AS as a function of the axial velocity: (**a**) λ = 200, (**b**) λ = 40; ((∘) AS-$\varpi_{1}$, (□) AS-$\varpi_{2}$, (△) AS-$\varpi_{3}$; (−−) without surface effect, (—) with surface effect).

To show the accuracy of the NDS-based model for beam-like moving nanostructure of our concern, we perform another comparison study. In the Supplementary Materials, Section S.4, we develop novel analytical solutions (AS) for capturing both axial and lateral vibrations of a moving nanochassis with moderate and high slenderness ratios. Through developing an efficient variation of the variable technique, the explicit expressions of both natural frequencies and divergence velocities of various vibration modes for a very lengthy moving nanochassis are presented and discussed in Section S.4.1 of the Supplementary Materials. Through generalizing the developed technique for lengthy nanochassis to those with moderate slenderness ratio, the nonlinear coupled relations of the characteristic equations are obtained and solved for the unknown parameters, particularly the flexural frequencies, as displayed in Section S.4.2 of the Supplementary Materials. In Figure 3a,b, the plots of the first three flexural frequencies as a function of the axial velocity for the moving nanochassis with the high and moderate slenderness ratios (i.e., λ = 40 and 200) have been depicted. The plotted results are based on the developed analytical approach in Section S.4 and those of the NDS-based model for two cases with surface energy effect and without consideration of the surface effect. As it is seen, the NDS-based model can reproduce the results of the analytical approach with a fairly good accuracy for most velocity levels. For higher vibration modes, the discrepancies between the predicted results by the NDS and those of the analytical solution become highlighted, particularly at velocities close to the critical ones and stockier nanochassis. This fact is mainly related to the incorporation of the shear effect to the transverse vibrations of moving nanochassis as well as the existing high interactions of shear and flexural modes of the moving nanochassis with lower slenderness ratios.

The proposed models herein may further be framed as part of a wider effort to understand multiscale structural performance under coupled physical effects, where stiffness tailoring, flexible-body control, and anisotropy-sensitive response are central themes [114,115,116]. Although many investigations come from optoelectronic and nanomaterial systems, they still support the manuscript’s broader nanoscale motivation by highlighting how geometry, interfaces, fabrication-dependent morphology, and surface-mediated behavior strongly affect functional response [117,118,119,120]. Finally, the diversity of the literature—from obstacle-avoidance control and geomechanics to instability analysis—suggests that the paper can be presented as a foundational mechanics study whose nonlinear nonlocal formulation may later inform more complex traveling nanostructures and multifunctional engineered systems [14,89,121].

### 5.2. Parametric Studies

In this part, inclusive numerical studies are carried out to explain the roles of various factors on the deformation fields and the resulting nonlocal forces within the moving excited nanochassis. According to the existing literature [8,122,123,124,125], the materials of the nanochassis are best understood as precisely synthesized molecular components, primarily from organic and carbon-rich molecules, rather than conventional engineering materials like steel, rubber, or silicon. Here, the nanochassis is assumed to be made from Ag molecules with circular cylindrical configuration and therefore its material properties were taken from the former published works; however, we can also take into account the above materials (aromatic/conjugated organic scaffolds, carbon-rich molecules, and so on) as well in our modeling for future works and then proceed with comparing their mechanical/dynamical behaviors with those provided in this research work.

Now, let us consider a circular cylindrical silver nanochassis with the following properties for the bulk and the surface layer [126,127]: $\rho_{b}$ = 10,500 kg/m^3^, $\rho_{s}$ = $10^{-7}$ kg/m^2^, $\nu_{b}$ = 0.26, $E_{b}$ = 76 GPa, $\lambda_{s}$ = 1 N/m, $\mu_{s}$ = 0.11 N/m, $\tau_{s}$ = 0.89 N/m, and $h_{0}$ = 4 nm. To investigate the problem more conveniently, we define the following normalized deformations and nonlocal forces:

(58) $$\begin{array}{c} u_{N}=\frac{\bar{X}}{{\bar{X}}_{st,max}},\;\;w_{N}=\frac{\bar{Y}}{{\bar{Y}}_{st,max}},\;\;N_{bN}^{nl}=\frac{{\bar{N}}_{b}^{nl}}{{\bar{N}}_{st,max}},\;\;M_{bN}^{nl}=\frac{{\bar{M}}_{b}^{nl}}{{\bar{M}}_{st,max}}. \end{array}$$

In these relations, ${\bar{X}}_{st,max}$ and ${\bar{N}}_{st,max}$ denote the dimensionless maximum static longitudinal displacement and the dimensionless maximum static nonlocal axial force within the fully pinned nanochassis due to its own weight when it is placed in the vertical position. Additionally, ${\bar{Y}}_{st,max}$ and ${\bar{M}}_{st,max}$ represent the dimensionless maximum deflection and the dimensionless maximum nonlocal flexural moment of the excited moving nanochassis due to its own bulk weight. For a given nanochassis whose properties are specified by an extra zero sub-index, these are given by:

(59) $$\begin{array}{c} {\bar{X}}_{st,max}=\frac{\rho_{b}A_{b0}l_{b0}^{2}g}{8E_{b}A_{b0}},\;\;\;{\bar{N}}_{st,max}=\frac{\rho_{b}A_{b0}l_{b0}g}{2},\;\\ {\bar{Y}}_{st,max}=\frac{5\rho_{b}A_{b0}l_{b0}^{4}g}{384E_{b}I_{b0}},\;\;\;{\bar{M}}_{st,max}=\frac{\rho_{b}A_{b0}l_{b0}^{2}g}{8}, \end{array}$$

where $A_{b0}$, $I_{b0}$, and $l_{b0}$ represent the cross-sectional area, inertia, and the length of the specified nanochassis with the slenderness ratio $\lambda_{0}$ = $l_{b0}\;\sqrt{\frac{A_{b0}}{I_{b0}}}$, respectively, and *g* is the gravitational acceleration. Additionally, the normalized velocity is defined by: $\beta_{v}=\frac{\beta_{x}}{\beta_{cr}}$, where $\beta_{cr}$ denotes the critical divergence velocity (see Equation (57)) in which is a function of the nanochassis geometry (i.e., mainly slenderness ratio), bulk properties (i.e., mass per unit length and bending rigidity), and surface factors (i.e., residual surface stress, surface layer geometry, density and bending rigidity).

Herein, we have used the first 9 modes for spatial discretization of both axial and transverse vibrations of the moving nanochassis (i.e., $NM_{X}$ = $NM_{Y}$ = 9). In addition, we have used an exponential kernel function in evaluating the nonlocal stresses and forces. To this end, each influence domain of the kernel function is divided into 10 subdomains and 6 Gaussian points are inserted in each one for calculating the integrals associated with both linear and nonlinear stiffness matrices. All numerical calculations of the mass and stiffness matrices associated with the nonlinear behavior of moving nanochassis have been run in MATLAB R2017b.

Regarding further details of the approach used for time discretization, we define $\tau_{fp}$ and $\tau_{cr}$ as the dimensionless fundamental period of the moving nanochassis and the dimensionless critical velocity, respectively. These are expressed as follows: $\tau_{fp}$ = $2\pi /\varpi_{1}$ and $\tau_{cr}$ = 1/$\beta_{cr}$, where $\varpi_{1}$ represents the dimensionless frequency and $\beta_{cr}$ the dimensionless critical velocity, as described in Section 5. Based on this, we establish two time factors, $T_{1}$ and $T_{2}$, defined as: $T_{1}$ = $\tau_{fp}$ /$l_{b}^{2}\sqrt{\left(\rho_{b}A_{b}\right)/\left(E_{b}I_{b}\right)}$ and $T_{2}$ = 4 max($T_{1}$, $T_{cr}$), where $T_{cr}$ is the critical time factor associated with $\tau_{c}r$. For the time discretization of the problem, we utilized 200 time steps in the interval [0,$T_{1}$] and 300 time steps in the interval [$T_{1}$, $T_{cr}$]. In addition, concerning Newton’s method convergence check, we apply the following convergence criterion at each time step: $∥(Y_{p,max}^{i+1}-Y_{p,max}^{i}∥$/$∥Y_{p,max}^{i+1}∥<10^{-3}$, where $Y_{p,max_{i}}$ denotes the maximum deflection of the moving nanochassis at the *i*-th iteration.

#### 5.2.1. Effect of the Nonlocality

In the lack of theoretical works and virtual experiments on the small-scale parameter of silver-based nanostructures, we try to rationally estimate it in the following. Martinez [128] performed a density functional analysis to clusters from ammonia metal to determine their bond length, binding energy, Mulliken atomic charges, and M-N stretching of vibration modes. By using Gaussian 98 and the hybrid B3LYP functional, her study shows that the Ag-Ag bond in Ag_2_, Ag_3_, and Ag_4_-based molecules in order are 2.611, 2.69, 2.811 °A. In another investigation, Dubiel et al. [129] scrutinized Ag-Ag bonds length subjected to various temperatures. It was revealed that the nearest Ag-Ag distance would be dissimilar to that of polycrystalline Ag baffle lower than 400 °K. Such a fact can be interpreted by a thermo-elastic model that deals with the discrepancy between the thermal expansion coefficient of Ag particles and that of the glass ground substance. The obtained results by the XRD at the room temperature indicated that the Ag-Ag bond length would be approximately equal to 2.89 °A. Further calculations displayed that how the bond length of Ag-Ag would vary in terms of the temperature. On the other hand, recent investigations show that the aspect ratio, boundary conditions, and chirality are among the crucial factors affecting the small-scale parameter of carbon nanotubes [130,131]. In most of the suggested nonlocal continuum-based models for these tiny structures, the value of $e_{0}$ is considered in the range of 0–16. By taking into account of the mean value of 8 for $e_{0}$ and the bond length of Ag-Ag atoms as 0.28 nm, the value of the small-scale parameter for the silver-based nanochassis would be $e_{0}a$ = 2.24 nm. With regard to the uncertainties on the value of the nonlocal parameter, we perform a sensitivity study to disclose the dependency of the obtained results by the proposed models to the nonlocal parameter.

**Figure 4:**
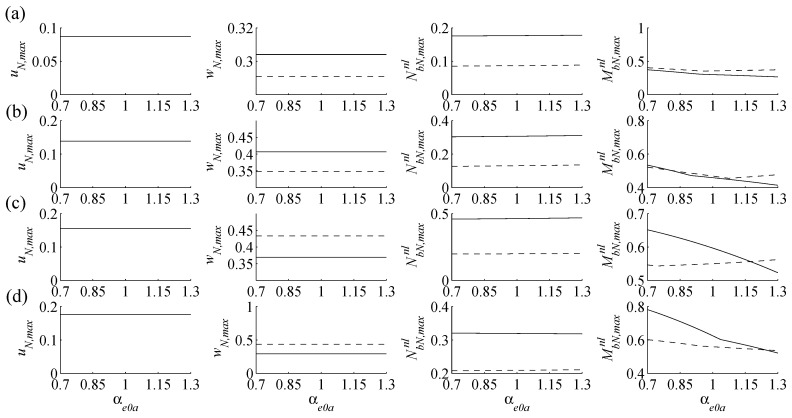
Effect of the nonlocality on the maximum nonlocal elastic fields for different velocities: (**a**) $\beta_{v}$ = 0.1, (**b**) $\beta_{v}$ = 0.2, (**c**) $\beta_{v}$ = 0.3, (**d**) $\beta_{v}$ = 0.4; ((−−) LM, (—) NM; $\alpha_{e_{0}a}=\frac{e_{0}a}{e_{0}a_{0}}$, $e_{0}a_{0}$ = 2.24 nm, λ=30, ${\bar{\varpi }}_{i}$ = 0.8$\varpi_{1}\left(\beta_{v}=0.4\right)$, ${\bar{a}}_{i}$ = 0.001).

Figure 4a–d display the role of the small-scale parameter on the predicted elastic fields of the moving excited nanochassis for four levels of the velocity (i.e., $\beta_{v}$ = 0.1, 0.2, 0.3, and 0.4). The results are provided for a moving nanochassis over a harmonic surface with the following properties: ${\bar{a}}_{i}$ = 0.001, ${\bar{\varpi }}_{i}$ = $0.8\varpi_{1}\left(\beta_{v}=0.4\right)$, where $\varpi_{1}\left(\beta_{v}=0.4\right)$ represents the fundamental frequency of the moving nanochassis of velocity $\beta_{v}$ = 0.4. The dashed and solid lines present the plotted results based on the LM (linear model) and NM (nonlinear model), respectively. As it is seen, the maximum nonlinear axial and lateral displacements slightly reduce by growing the small-scale parameter while the predicted axial force within the moving nanochassis would slightly increase by increasing the nonlocality. Additionally, the rate of reduction is more apparent for the plots of maximum flexural moment, particularly for higher levels of speed. For all considered levels of the speed as well as the small-scale factor, the predicted linear longitudinal displacement is equal to zero. For a given small-scale factor, the nonlinear longitudinal displacement as well as the nonlocal flexural moment would commonly increase by increasing the speed whereas the nonlinear nonlocal axial force would grow with speed up to $\beta_{v}$ = 0.3. A tightly mindful investigation of the demonstrated results show that the discrepancies between the LM-based axial force and the NM-based axial force generally reduce by increasing the small-scale factor. For very low speeds (i.e., $\beta_{v}$ = 0.1 and 0.2), the predicted NM-based bending moment is lower than the LM-based one and their differences increase by growing the nonlocality; however, for higher levels of speed (i.e., $\beta_{v}$ = 0.3 and 0.4) and $\alpha_{e0a}<1.2$, the predicted NM-based results are higher than those obtained by the LM and their differences would lessen by increasing the small-scale parameter.

#### 5.2.2. Effect of the Nanochassis’ Diameter

**Figure 5:**
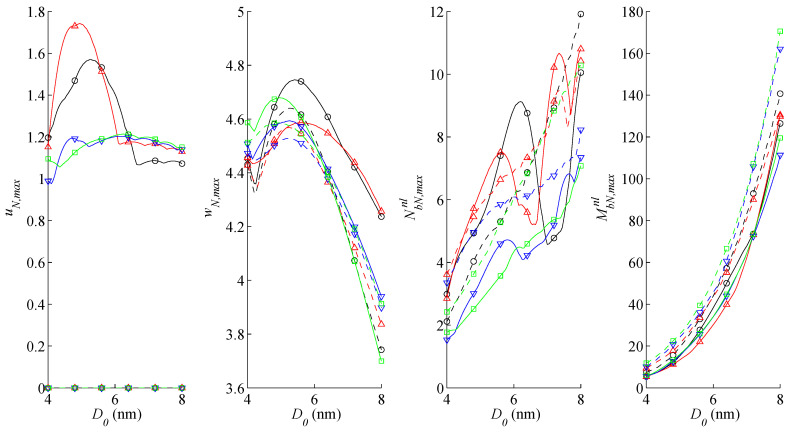
Effect of the nanochassis’s diameter on the maximum nonlocal elastic fields for different velocities: ((∘) $\beta_{v}$ = 0.1, (△) $\beta_{v}$ = 0.3, (▽) $\beta_{v}$ = 0.5, (□) $\beta_{v}$ = 0.7; ((−−) LM, (—) NM; $e_{0}a$ = 2.24 nm, λ=30, ${\bar{\varpi }}_{i}$ = 0.5$\varpi_{1}\left(\beta_{v}=0.5\right)$, ${\bar{a}}_{i}$ = 0.001).

For four levels of the speed (i.e., $\beta_{v}$ = 0.1, 0.3, 0.5, and 0.7), the plots of the nonlocal elastic fields based on the proposed nonlinear NIS-based model are depicted in Figure 5. The provided results are for a nanochassis with λ = 30 moves over a harmonic surface with the following properties: ${\bar{a}}_{i}$ = 0.001 and ${\bar{\varpi }}_{i}$ = 0.5$\varpi_{1}\left(0.5\beta_{cr}\right)$. The presented results based on the LM and NM are demonstrated by the dashed and solid lines, respectively. As it is observed from the depicted results, both maximum axial and lateral displacements plots take their peak points at a particular level of $h_{0}$; however, these particular levels are not the same for these components of displacements. After the peak point, the predicted nonlinear maximum longitudinal displacements would commonly fluctuate as a function of nanochassis’s diameter while the maximum deflections based on both LM and NM would reduce as the diameter enlarges. It should be noticed that the exerted harmonic force on the moving nanochassis over a harmonic surface (i.e., $\left(\rho_{b}A_{b}+\rho_{s}A_{s}\right)\;{\bar{a}}_{i}{\bar{\omega }}_{i}^{2}\;\sin\left(\omega_{i}\tau \right)$) magnifies fairly in a parabolic manner as a function of its diameter, however, the bending rigidity of the moving nanochassis is a fourth-order polynomial in terms of the diameter. This issue interprets the descending branch of the plots of the maximum deflection. For most of the cases, the predicted NM-based maximum longitudinal displacements and deflections are greater than those predicted by the LM. Regarding the maximum nonlocal axial force, its plots present some fluctuations and the general trend of the demonstrated plots are ascending in terms of the diameter. Generally, the maximum nonlocal flexural moment increases by growing the nanochassis’s diameter. In most of the cases, the predicted maximum longitudinal displacement and flexural moment by the NM are lower than those predicted by the LM. More scrutiny reveals that the relative discrepancies between the maximum nonlocal flexural moments of these two models would reduce by increasing the nanochassis’s diameter; however, the relative differences between the NM-based maximum deflections and the LM-based maximum deflections would increase by increasing the nanochassis’s diameter.

**Figure 6:**
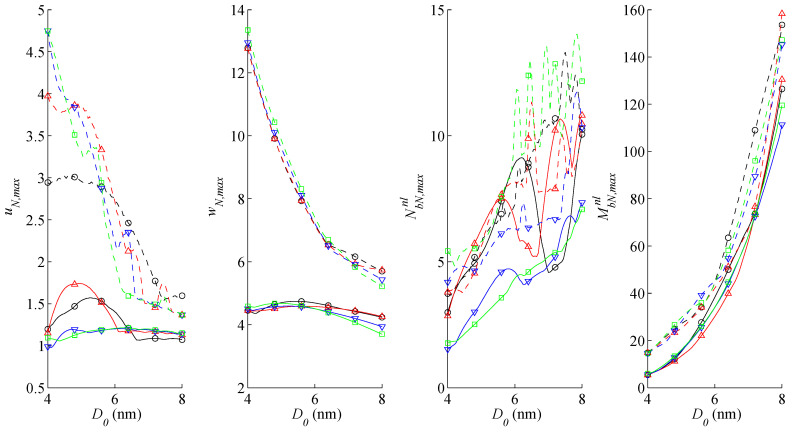
Effect of the nanochassis’s diameter on the maximum nonlocal elastic fields for different velocities: ((∘) $\beta_{v}$ = 0.1, (△) $\beta_{v}$ = 0.3, (▽) $\beta_{v}$ = 0.5, (□) $\beta_{v}$ = 0.7; (−−) without surface effect, (—) with surface effect; $e_{0}a$ = 2.24 nm, λ=30, ${\bar{\varpi }}_{i}$ = 0.5$\varpi_{1}\left(\beta_{v}=0.5\right)$, ${\bar{a}}_{i}$ = 0.001).

Another scrutiny is going to be conducted to disclose the surface energy effect on the nonlinear dynamic response of the moving excited nanochassis. In Figure 6, the plots of the maximum displacement components as well as the maximum axial force and flexural moment of the beam-like nanochassis as a function of its diameter have been presented for four levels of the velocity (i.e., $\beta_{v}$ = 0.1, 0.3, 0.5, and 0.7), and other surface irregularities and geometry parameters are the same as those given in the previous part. For all considered levels of the nanochassis’s diameter, the predicted surface energy-based displacements as well as nonlocal flexural moment are lower than their corresponding values without considering the surface energy; however, such a fact does not hold true for the plots of the maximum nonlocal axial force for all considered velocities and nanochassis diameters. Moreover, the relative discrepancies between the maximum values of axial and lateral displacements as well as nonlocal bending moment accounting for the surface energy and their counterparts without considering the surface energy effect would reduce as the nanochassis’s diameter grows. This fact is mainly related to the reduction in the ratio of the surface area to the bulk volume by increasing the diameter, leading to the lessening of the ratio of the total surface energy to the bulk’s total energy. As a result, the predicted results by consideration of the surface energy would approach to those obtained without its considering.

#### 5.2.3. Effect of the Nanochassis’ Length

**Figure 7:**
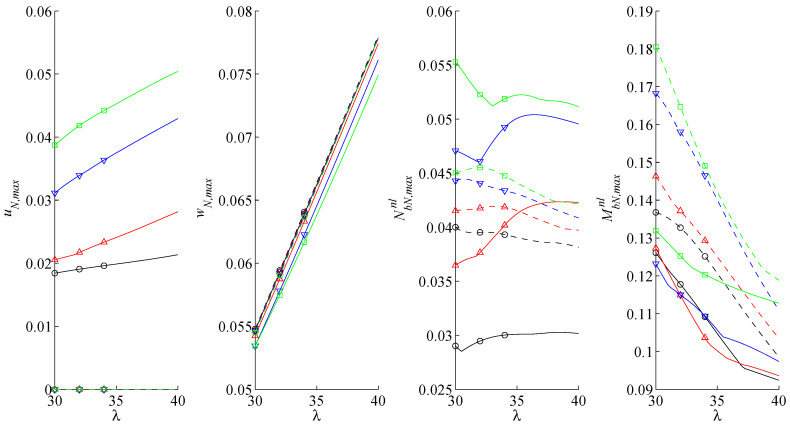
Effect of the nanochassis’s length on the maximum nonlocal elastic fields for different velocities: ((∘) $\beta_{v}$ = 0.1, (△) $\beta_{v}$ = 0.3, (▽) $\beta_{v}$ = 0.5, (□) $\beta_{v}$ = 0.7; (−−) LM, (—) NM; $e_{0}a$ = 2.24 nm, ${\bar{\varpi }}_{i}$ = 0.5$\varpi_{1}\left(\beta_{x}=0.5\beta_{cr0}\right)$, ${\bar{a}}_{i}$ = 0.001$\left(\frac{\lambda_{0}}{\lambda }\right)$, $\lambda_{0}$ = 40).

An important study is carried out to reveal the influence of the length of the excited moving nanochassis on the maximum axial and lateral displacements as well as maximum nonlocal axial force and flexural moment. In Figure 7, the plots of the maximum nonlocal surface energy-based elastic fields associated with axial and lateral vibrations of the moving excited nanochassis have been presented for four levels of its velocity (i.e., $\beta_{v}$ = 0.1, 0.3, 0.5, and 0.7). The plotted results on the basis of the LM and the NM are, respectively, demonstrated by the dashed and solid lines. Because of the dependency of the dimensionless amplitudes and the excitation frequency to the nanochassis length, we define the following dimensionless quantities: ${\bar{\varpi }}_{i}$ = ${\left(\frac{\lambda }{\lambda^{*}}\right)}^{2}\;{\bar{\varpi }}_{i}^{*}$ and ${\bar{a}}_{i}=\left(\frac{\lambda^{*}}{\lambda }\right)\;{\bar{a}}_{i}^{*}$, where herein ${\bar{\varpi }}_{i}^{*}$ = $0.5\varpi_{i}\left(\beta_{x}=0.5\beta_{cr}^{*}\right)$, ${\bar{a}}_{i}^{*}$ = 0.001, and $\lambda^{*}$ = 40. Concerning the maximum nonlinear longitudinal displacement, its value increases by growing of the nanochassis’s length such that the rate of increase is more apparent for higher levels of the velocity. For each value of the slenderness ratio, there exists a relative discrepancy of about 100 percent between the LM-based results and those of the NM since the LM cannot capture any longitudinal displacement within the moving excited nanochassis. For each level of the slenderness ratio, the nonlocal axial force increases with the increase in the nanochassis velocity. This fact is confirmed by both the LM and the NM. According to the LM, the values of the nonlocal axial force would generally lessen by increasing the slenderness ratio; however, the plots of the maximum nonlocal axial force based on the NM take their relative minimum and maximum at particular levels of slenderness ratios. For slenderness ratios higher than those pertinent to the relative peak points, the demonstrated plots indicate that the value of the maximum nonlocal force lessens as the slenderness ratio magnifies. The depicted results manifestly show that the predicted maximum deflections grow linearly as a function of the slenderness ratio for all considered levels of the velocity. However, the predicted nonlinear deflections are commonly lower than those predicted by the LM. Furthermore, the predicted maximum nonlocal bending moment within the moving excited nanochassis would reduce by increasing the slenderness ratio. Generally, the NM-based flexural moments are lower than those of the LM, and their discrepancies would considerably reduce by enlarging the slenderness ratio.

#### 5.2.4. Effect of the Nanochassis’ Velocity

**Figure 8:**
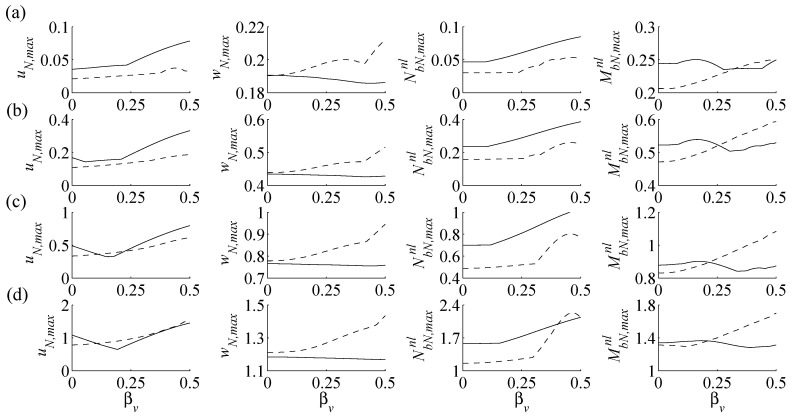
Effect of the velocity on the maximum nonlocal elastic fields for different frequencies of the harmonic route: (**a**) ${\bar{\varpi }}_{i}$ = 0.1$\varpi_{1}$, (**b**) ${\bar{\varpi }}_{i}$ = 0.2$\varpi_{1}$, (**c**) ${\bar{\varpi }}_{i}$ = 0.3$\varpi_{1}$, (**d**) ${\bar{\varpi }}_{i}$ = 0.4$\varpi_{1}$; ((−−) LM, (—) NM; $e_{0}a$ = 2.24 nm, $\varpi_{1}$ = $\varpi_{1}\left(\beta_{x}=0\right)$, λ=30, ${\bar{a}}_{i}$ = 0.001).

The influence of the velocity of the nanochassis on the nonlinear axial and lateral vibrations of the moving excited nanochassis is of particular interest. For this purpose, the plots of the maximum axial and lateral deformations as well as those of the maximum nonlocal axial force and flexural moment as a function of nanochassis’ velocity are demonstrated in Figure 8a–d for four levels of the excitation frequency (i.e., ${\bar{\varpi }}_{i}$/$\varpi_{1}\left(\beta_{x}=0\right)$ = 0.1, 0.2, 0.3, and 0.4) in the case of λ = 30 and ${\bar{a}}_{i}$ = 0.001. The nonlinear surface energy-based results and those without consideration of the surface energy have been presented by the solid and dashed lines, respectively. As it is observed, for low levels of the excitation frequency, the predicted nonlinear longitudinal displacement grows as the velocity of the excited nanochassis increases. Further, for all considered levels of the velocity, the nonlinear surface energy-based longitudinal displacement is greater than that obtained without considering the surface energy effect, and the discrepancies between these two values would commonly grow by increasing the velocity of the moving nanochassis. As the excitation frequency increases, the trend of the plots of the maximum surface energy-based longitudinal displacement as a function of the velocity changes. For $\beta_{v}$ = 0.3 and 0.4, the aforementioned plots consist of two distinct branches: a descending branch and an ascending one. Such a specific trend for these plots leads to the fact that the maximum surface energy-based longitudinal displacements for some levels of the velocity (i.e., velocities close to that associated with the minimum point) would be lower than those without considering the surface effect. Regarding the trend of the nonlinear deflection, the predicted surface energy-based deflection reduces as the velocity grows while that obtained without consideration of the surface effect grows with the velocity. For all considered levels of the velocity, the nonlinear deflection by consideration of the surface effect is lower than that obtained without consideration of the surface effect. This issue results from two facts; firstly, the positive value of the residual surface stress results in the axial residual tensile force within the moving nanochassis, and thereby, its flexural stiffness increases due to the appearance of the geometrical stiffness term in the transverse equations of motion (i.e., $-\chi_{4}\;\frac{\partial^{2}{\bar{Y}}_{p}}{\partial \eta^{2}}$ in both NDS-based and NIS-based governing equations, see Equations (31b) and (54)); secondly, positive incorporation of the surface energy effect into the whole bending rigidity of the moving nanochassis (for instance, the appearance of the term $\chi_{3}\;\frac{\partial^{4}{\bar{Y}}_{p}}{\partial \eta^{4}}$ in the NDS-based governing equations, see Equation (31b)).

For all considered values of the excitation frequency, the predicted nonlinear surface energy-based axial force within the moving excited nanochassis increases as its velocity increases. For most values of velocities, the nonlinear axial force by considering the surface effect is higher than that obtained through excluding the surface energy. Concerning the nonlinear nonlocal flexural moment, for velocities lower than a particular value, those obtained by considering the surface effect are higher than those predicted without consideration of the surface effect. For such a range of the velocity, the relative discrepancies between these two results lessen by increasing the velocity up to that particular level; however, for velocities higher than that particular level, those calculated based on the surface effect are lower than those without considering the surface effect. Further scrutiny shows that the relative discrepancies between the obtained results with and without consideration of the surface effect commonly grow by increasing the velocity. This conclusion is valid for all considered levels of the excitation frequency.

#### 5.2.5. Effect of the Amplitude of the Harmonic Route

**Figure 9:**
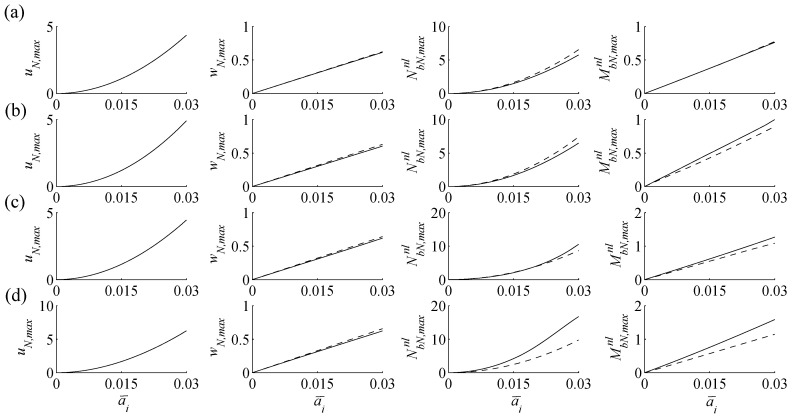
Effect of the amplitude of the harmonic route on the maximum nonlocal elastic fields for different route’s frequencies: (**a**) $\beta_{v}$ = 0.1, (**b**) $\beta_{v}$ = 0.2, (**c**) $\beta_{v}$ = 0.3, (**d**) $\beta_{v}$ = 0.4; ((−−) LM, (—) NM; $e_{0}a$ = 2.24 nm, λ=50, $\lambda_{0}$ = 50, ${\bar{\varpi }}_{i}$ = 0.4$\varpi_{1}\left(\beta_{v}=0.4\right)$).

In Figure 9a–d, the plots of the nonlocal surface energy-based elastic fields of the moving excited nanochassis in terms of the harmonic surface amplitude are presented. The results are given for four velocity levels (i.e., $\beta_{v}$ = 0.1, 0.2, 0.3, and 0.4), λ = 50, and ${\bar{\varpi }}_{i}/\varpi_{1}\left(\beta_{x}=0.4\right)$ = 0.4. Without loss of generality, it is assumed that the excitation amplitudes of both ends are the same (i.e., $a_{1}$ = $a_{2}$). The dashed lines and the solid lines in order are pertinent to the predicted results by the LM and NM. Generally, the maximum nonlinear axial and lateral displacements as well as the maximum nonlinear axial force and flexural moment magnify as the surface amplitude increases. As it is observed that the predicted maximum linear longitudinal frequency is zero for all levels of the surface amplitude, the nonlinear maximum longitudinal displacement varies in a hyperbolic manner as a function of the surface amplitude. Concerning the maximum dynamic deflection, both linear and nonlinear values vary linearly in terms of the surface amplitude, and the predicted nonlinear ones are somewhat lower than those of the linear ones, and their relative differences generally reduce by increasing the surface amplitude. The predicted values of the maximum axial force within the moving excited nanochassis, both linear and nonlinear ones, would vary hyperbolically as a function of the amplitude. For low levels of the velocity (i.e., $\beta_{v}$ = 0.1 and 0.2), the nonlinear maximum deflections are lower than the maximum linear deflections for all considered values of the surface amplitude; however, for fairly high levels of velocities (i.e., $\beta_{v}$ = 0.3 and 0.4), the predicted nonlinear maximum deflections are higher than those obtained by the LM. Additionally, their relative differences would also magnify by growing the amplitude of the harmonic route. Regarding the maximum value of the nonlocal surface energy-based bending moment, both the linear and nonlinear values increase linearly in terms of the surface amplitude, and the predicted results by the NM are commonly greater than those of the LM. Further scrutinizes reveal that the relative differences between these values would increase by growing the surface amplitude, and such a fact is more obvious for higher levels of the velocity.

#### 5.2.6. Effect of the Wavelength of the Harmonic Route

**Figure 10:**
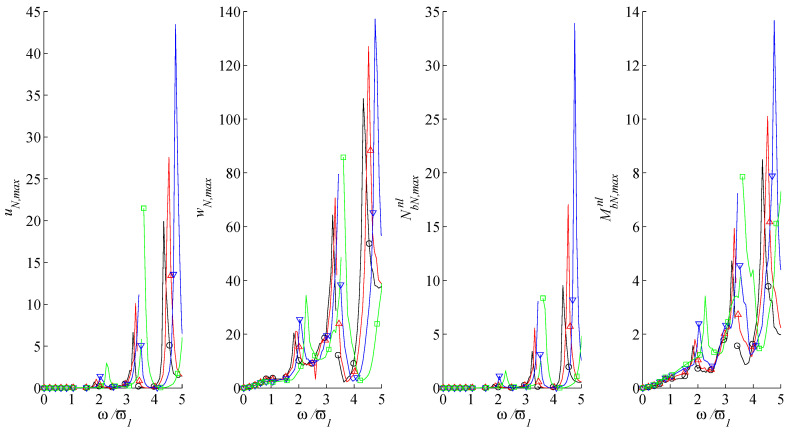
Effect of the harmonic route’s wavelength on the maximum nonlocal elastic fields for different velocities: ((∘) $\beta_{v}$ = 0.1, (△) $\beta_{v}$ = 0.2, (▽) $\beta_{v}$ = 0.3, (□) $\beta_{v}$ = 0.4; $e_{0}a$ = 2.24 nm, ${\bar{a}}_{i}$ = 0.0001, λ = 40).

Another interesting parametric study is performed to show the role of the wavelength of the harmonic surface on the nonlinear nonlocal surface energy-based elastic fields of the moving excited nanochassis. In Figure 10, the plots of the maximum axial and lateral displacements as well as the maximum axial force and flexural moment have been demonstrated for four levels of the nanochassis’s velocity (i.e., $\beta_{v}$ = 0.1, 0.2, 0.3, and 0.4) in the case of ${\bar{a}}_{i}$ = 0.0001 and λ = 50. The obtained results clearly display that the maximum values of elastic fields, particularly those of deflection and flexural moment, would grow by increasing the surface frequency up to the fundamental frequency of the moving nanochassis such that the rate of growth is more apparent at the surface frequencies close to the fundamental one. For route frequencies between the fundamental frequency and the second natural frequency, two distinct branches are detectable for all considered levels of the velocity: a descending branch and an ascending branch. In the descending branch, the maximum deflection as well as the maximum flexural moment would smoothly decrease up to a particular level of frequency, and after that (i.e., for the ascending branch), by increasing the route frequency, these values would sharply increase with the surface frequency. According to the demonstrated results, such a scenario for variation of the maximum elastic fields is repeated for all considered velocity levels when the surface frequency varies between the third and the fourth natural frequencies. These obtained results also display that at the surface frequencies close to the natural frequencies, variation of the velocity of the moving nanochassis has the most impact on the variation in the nonlocal surface energy-based elastic fields.

## 6. Several Avenues for Future Research Work

Although the present manuscript is focused on the theoretical formulation and numerical investigation of nonlinear vibrations of a moving beam-like nanochassis, several important avenues for future research can be identified based on the current framework:

1. Experimental validation of the model can be followed as a hot topic for future work. Since the manuscript validates its results against analytical solutions rather than experiments, a key next step is to test fabricated nanoscale beam/chassis systems and compare measured vibrations and deflections with the model predictions, especially for nonlocal and surface effects.
2. This work can be extended to multiphysics loading. The paper references nanosystems driven by electrical and thermal fields, but the present study mainly focuses on mechanical vibration; therefore, thermomechanical and electromechanical coupling would be a significant extension to explore the problem in a more general context.
3. Another crucial issue is the inclusion of damping and energy dissipation in the dynamical analysis of the problem. Because damping is not emphasized in the retrieved discussion, incorporating viscous, structural, or nanoscale dissipation mechanisms would improve realism for practical dynamic response analysis.
4. The existing wheels at the ends of the moving nanochassis are assumed to be rigid and massless. This assumption indicates that the disturbances experienced under the rotating wheels, caused by the roughness of the route, will be transmitted directly to the ends of the flexible nanochassis due to its movement. However, it is important to note that the wheels are actually flexible. Therefore, their linear and nonlinear stiffness, damping, and mass—collectively referred to as the dynamical factors of the wheels—should be properly considered, especially when more precise dynamic predictions of the wheel-nanochassis-nanosystems are required.
5. Broader instability and control studies can be followed by upcoming investigations. The present study examines potential instabilities, so future work could investigate instability suppression, active/passive control strategies, and safe operating regimes for moving nanochassis systems.
6. The current geometry and boundary conditions can be generalized to cover a more extensive range of problems. Since beam-like assumptions and boundary condition formulations are important in the current model, future research could address more complex geometries, support conditions, route irregularities, and heterogeneous material systems.
7. The study of more realistic nanoscale environments can be considered as a hot topic for future work. The effects of manufacturing imperfections, stronger surface irregularities, and additional environmental factors could be included to better approximate real operating conditions for nano-transport systems.
8. The shear effect has largely been overlooked in both linear and nonlinear models, primarily because the aspect ratio of the nanochassis ($l_{b}$/$h_{0}$) is quite high. This high aspect ratio supports the main hypothesis of Rayleigh beam theory, which states that planes perpendicular to the neutral axis remain perpendicular to the deformed neutral axis after deformation. However, this critical effect is expected to have a significant impact on the deformation of moving nanochassis that navigate rough surfaces, particularly when the aspect ratios are lower. Under such circumstances, we should use suitable shear-deformable beam theories to appropriately capture the linear and nonlinear vibrations of moving nanochassis.
9. One important aspect to consider for future studies is the examination of various numerical methods, such as the spectral element method, finite element method, and meshless methods, for solving the nonlinear coupled partial differential equations (PDEs) related to the longitudinal and transverse vibrations of moving nanochassis traveling over a rough path. It may also be beneficial to evaluate their convergence to determine the most effective approach for this type of problem, ensuring the highest possible accuracy in predicting dynamic behavior.

## 7. Conclusions

This paper was devoted to nonlinear axial and lateral vibrations of moving beam-like nanochassis over an arbitrarily shaped surface. Using an appropriate beam theory, the nonlinear surface energy-based governing equations for the moving nanochassis were presented based on the NDS and NIS models. By implementing the Galerkin methodology through exploiting assumed modes and variable of variations approach, the resulting nonlinear governing equations of each model were reduced to their corresponding ordinary differential equations. Then, these were solved for the unknown time-dependent factors using the finite-difference method and Newton’s approach, and the axial and lateral displacements as well as the axial force and flexural moment were calculated. In the lack of experimentally observed data or virtual experiments based on atomistic-based approaches, the obtained results by the suggested models were compared with those of newly developed analytical solutions for particular cases and reasonably remarkable results were reported. Subsequently, an inclusive parametric study was conducted and the roles of crucial factors, including surface energy, nonlinearity, length and height of the beam-like nanochassis, amplitude and surface frequency of the nanochassis ends, and nanochassis velocity, on the extreme nonlinear dynamic response of the excited moving nanochassis were revealed carefully. The potential dynamic instability of the excited moving nanostructure was also discussed briefly. The main obtained results can be summarized by the following points:

1. The NDS-based numerical model shows strong agreement with exact analytical solutions for nonlocal elastic fields and the natural frequencies of moving nanochassis. Small deviations mainly appear in higher vibration modes or near critical speeds, where coupled bending–shear interactions become more pronounced.
2. Increasing the small-scale (nonlocal) parameter generally reduces the maximum nonlinear axial and lateral displacements, while slightly increasing the axial force. The reduction is especially noticeable for the maximum bending moment, particularly when the nanochassis moves at higher speeds.
3. The maximum axial and lateral displacements reach peak values at certain diameters, but overall deflections tend to decrease as the diameter increases. This behavior is largely attributed to the strong dependence of bending stiffness on diameter, especially through its fourth-power relationship.
4. Including surface energy leads to smaller nonlocal displacements and bending moments compared with models that neglect it. However, this difference becomes less significant as the nanochassis diameter increases, which is consistent with the decreasing surface-area-to-volume ratio in larger structures.
5. The maximum nonlinear longitudinal displacement increases with nanochassis length, especially at higher speeds. The results also reveal a complex speed-dependent response: at low excitation frequencies displacement rises with speed, while at higher frequencies the trend becomes nonlinear and less predictable.
6. The results indicate that linear and nonlinear analyses can differ substantially, especially for axial response and at higher transport speeds. Nonlinear calculations capture stronger coupling and larger response variations, showing that linear models may underestimate or misrepresent the actual mechanical behavior of the moving nanochassis.
7. The results suggest that the moving nanochassis may approach unstable behavior as its speed nears critical values, where response amplitudes and modal interactions become more sensitive. This indicates a heightened risk of dynamic instability, particularly when higher vibration modes and coupled axial–bending effects are significant.

Although the manuscript itself is limited to theoretical modeling and numerical analysis, the reported study could in principle motivate future experimental work. Such validation would require fabrication of representative nanostructures, such as beam-like nanochassis systems, with controlled geometry and material properties. Their dynamic responses—especially natural frequencies, vibration amplitudes, and deflections—could then be measured under varying axial velocities and diameters, and compared with the model predictions. Because the paper emphasizes nonlocal and surface-energy effects, any practical validation would also need sufficiently precise nanoscale fabrication and metrology to detect those size-dependent behaviors. However, the manuscript does not itself present an experimental setup or experimental validation; its validation is analytical/numerical rather than physical.

To reveal the influence of the shear strain energy on vibrations of excited moving nanochassis, application of shear deformable continuum-based beams to their nonlinear dynamical analysis and potential instability is highly recommended to interested scholars and nanoengineers. Additionally, both linear and nonlinear mechanical analysis of transporting nanosystems with double or multiple nanochassis could be considered as hot topics for future works.

## Data Availability

Data are available upon request.

## Supplementary Materials

The following supporting information can be downloaded at: https://www.mdpi.com/article/10.3390/nano16120768/s1, Section S.1. Evaluation of $\frac{\partial \tilde{p}}{\partial \bar{q}}$ for the NDS-based model. Section S.2. Evaluation of $\frac{\partial \tilde{p}}{\partial \bar{q}}$ for the NIS-based model. Section S.3. Analytical dynamic analysis of stationary nanochassis under arbitrarily excited ends via NDS. Section S.4. Exact linear free vibration of moving nanochassis accounting for surface effect.

## Appendix A. Some Insights Behind the Time-Forced Problem’s Assumptions

Under the assumptions stated in Section 2 and also explained with more details in the following, the problem of a nanochassis moving at constant speed over a sinusoidal route can be rationally replaced by an equivalent problem of a fixed nanochassis with time-dependent base excitations applied at its two ends. This replacement is valid because the chassis only “feels” the surface through the prescribed vertical displacements at its contact/support points. If the chassis moves with constant horizontal speed over a known surface profile y(s), then the vertical motion seen at each end is just that same spatial profile sampled in time.

A moving-structure problem can be rationally reduced to a fixed-structure, time-forced problem. In a more detail, such an equivalence holds when: (1) The speed is constant; (2) The surface profile is prescribed and unaffected by the chassis; (3) The contact can be modeled as imposed support motion; (4) The slope is small, so horizontal speed component satisfies $V_{s}\approx V$, (5) The beam length is much smaller than the wavelength, $l_{b}\ll L$, yielding further approximation of $W_{g_{1}}\left(t\right)\approx W_{g_{2}}\left(t\right)$.

Based on the geometric description of the route, it can be mathematically provided by:

(A1) $$\begin{array}{c} y\left(s\right)=a_{0}\sin\left(\frac{2\pi s}{L}\right), \end{array}$$

where $a_{0}$ represents the amplitude of the surface waviness, *L* denotes the wavelength, and *s* stands for the horizontal coordinate along the route. In addition, the nanochassis’s length is $l_{b}$, and has a constant forward speed V approximately horizontal. Let the left and right ends of the chassis be located at horizontal coordinates $s_{1}\left(t\right)$ and $s_{2}\left(t\right)$, respectively. If the chassis moves rigidly along the route with constant horizontal speed $V_{s}$, then $s_{1}\left(t\right)=l_{1}+V_{s}t$ and $s_{2}\left(t\right)=l_{2}+V_{s}t$, with $l_{2}=l_{1}+l_{b}$. Then, the surface elevations beneath the two ends are stated by: $W_{g_{1}}\left(t\right)=y\left(s_{1}\left(t\right)\right)$ and $W_{g_{2}}\left(t\right)=y\left(s_{2}\left(t\right)\right)$. By substituting y(s), one can arrive at

(A2) $$\begin{array}{c} W_{g_{1}}\left(t\right)=a_{0}\sin\left(\frac{2\pi }{L}\left(l_{1}+V_{s}t\right)\right),\;W_{g_{2}}\left(t\right)=a_{0}\sin\left(\frac{2\pi }{L}\left(l_{2}+V_{s}t\right)\right), \end{array}$$

or equivalently,

(A3) $$\begin{array}{c} W_{g_{1}}\left(t\right)=a_{0}\sin\left[2\pi \left(\frac{V_{s}}{L}t+\frac{l_{1}}{L}\right)\right],\;W_{g_{2}}\left(t\right)=a_{0}\sin\left[2\pi \left(\frac{V_{s}}{L}t+\frac{l_{2}}{L}\right)\right]. \end{array}$$

Based on the above assumptions, it can be rationally concluded that these are exactly the given excitations. In the following, some further technical explanations on this are also provided.

A moving beam over a stationary surface and a stationary beam over a moving surface are kinematically equivalent if the only relevant input is the relative vertical displacement at the supports/contacts. In general, the beam equations mainly depend on the beam inertia, internal elasticity, and support motion. If the support motions imposed on the beam are the same functions of time in both descriptions, then the beam response is the same. So instead of saying “the beam moves through a spatially varying profile”, we can say: “the beam is fixed in space, and the profile passes underneath it, producing time-varying support displacements”. These two viewpoints generate the same end inputs $W_{g_{1}}\left(t\right)$ and $W_{g_{2}}\left(t\right)$.

Suppose the route is given in space by y(s). A point moving over the route with coordinate $s\left(t\right)=s_{0}+V_{s}t$ experiences vertical input $w_{g}\left(t\right)=y\left(s_{0}+V_{s}t\right)$; this is simply a spatial-to-temporal mapping. For the sinusoidal route, $w_{g}\left(t\right)=a_{0}\sin\left(\frac{2\pi }{L}\left(s_{0}+V_{s}t\right)\right)$, we define the excitation frequency $\omega_{g}=\frac{2\pi V_{s}}{L}$, then $w_{g}\left(t\right)=a_{0}\sin\left(\omega_{g}t+\phi \right);\;\phi =\frac{2\pi s_{0}}{L}$. So, the moving-over-wavy-route problem becomes a fixed-beam problem with harmonic base excitation of frequency as follows: $f_{g}=\frac{V_{s}}{L}$, representing the standard traveling-profile-to-time-excitation conversion. In order to find the relationship between the two end excitations, using $l_{2}=l_{1}+l_{b}$, $W_{g_{2}}\left(t\right)=a_{0}\sin\left[2\pi \left(\frac{V_{s}}{L}t+\frac{l_{1}+l_{b}}{L}\right)\right]$. Hence, $W_{g_{2}}\left(t\right)=a_{0}\sin\left[2\pi \left(\frac{V_{s}}{L}t+\frac{l_{1}}{L}\right)+\frac{2\pi l_{b}}{L}\right]$. Therefore, the right-end excitation is identical to the left-end excitation with phase lag: $\Delta \phi =\frac{2\pi l_{b}}{L}$. Therefore, the exact relation reads: $W_{g_{2}}\left(t\right)=a_{0}\sin\left(\omega_{g}t+\phi_{1}+\Delta \phi \right)$, where $\phi_{1}=\frac{2\pi l_{1}}{L}$. If $l_{b}\ll L$, then $\Delta \phi =\frac{2\pi l_{b}}{L}\ll 1$. Thus, $W_{g_{2}}\left(t\right)\approx W_{g_{1}}\left(t\right)$, indicating that under $l_{b}\ll L$, the two ends essentially undergo the same vertical base motion.

In continuing, we would like to prove $V_{s}\approx V$ under $a_{0}\ll L$ condition, as well as $W_{g_{1}}\approx W_{g_{2}}$ under $l_{b}\ll L$ condition. To this end, let’s start with the surface equation: $y\left(s\right)=a_{0}\sin\left(\frac{2\pi s}{L}\right)$, where its slope is evaluated by: $\frac{dy}{ds}=\frac{2\pi a_{0}}{L}\cos\left(\frac{2\pi s}{L}\right)$. Hence, the maximum slope is, ${\left|\frac{dy}{ds}\right|}_{\max}=\frac{2\pi a_{0}}{L}$. If $a_{0}\ll L$, then $\frac{2\pi a_{0}}{L}\ll 1$, so the route is gently sloped. Therefore, the motion is nearly horizontal, and the horizontal component of velocity satisfies $V_{s}\approx V$. More precisely, if θ is the local route angle, then $\tan\theta =\frac{dy}{ds}$. For a small slope, θ is small, and cosθ≈1. Thus, $V_{s}=V\cos\theta \approx V$.

On the other hand, we want to use the small-beam-length approximation $l_{b}\ll L$ and its obtained results. For this, let us quantify the difference between $W_{g_{1}}$ and $W_{g_{2}}$. Using Taylor expansion:

(A4) $$\begin{array}{c} y\left(s+l_{b}\right)\approx y\left(s\right)+l_{b}\;y^{\prime }\left(s\right)+\frac{l_{b}^{2}}{2}y^{\prime \prime }\left(s\right)+\cdots , \end{array}$$

therefore,

(A5) $$\begin{array}{c} W_{g_{2}}\left(t\right)-W_{g_{1}}\left(t\right)\approx l_{b}\;y^{\prime }\left(s_{1}\left(t\right)\right). \end{array}$$

Since

(A6) $$\begin{array}{c} y^{\prime }\left(s\right)=\frac{2\pi a_{0}}{L}\cos\left(\frac{2\pi s}{L}\right), \end{array}$$

the maximum difference can be approximately given by:

(A7) $$\begin{array}{c} |W_{g_{2}}-W_{g_{1}}{|}_{\max}\approx l_{b}\frac{2\pi a_{0}}{L}. \end{array}$$

Therefore, if $l_{b}\ll L$,

(A8) $$\begin{array}{c} |W_{g_{2}}-W_{g_{1}}{|}_{\max}\ll a_{0}, \end{array}$$

which confirms that the end excitations are nearly identical.

As the final conclusion, the mechanical problem of a moving nanochassis over a harmonic route can be replaced by that of a fixed nanochassis subjected to a moving route beneath it, represented by the end excitations:

(A9) $$\begin{array}{c} W_{g_{1}}\left(t\right)=a_{0}\sin\left[2\pi \left(\frac{V_{s}}{L}t+\frac{l_{1}}{L}\right)\right],\;\;\;W_{g_{2}}\left(t\right)=a_{0}\sin\left[2\pi \left(\frac{V_{s}}{L}t+\frac{l_{2}}{L}\right)\right]. \end{array}$$

This relation is valid because the moving-over-profile problem can be converted into time-dependent support displacements by sampling the spatial profile along the moving contact points. If in addition

(A10) $$\begin{array}{c} a_{0}\ll L;\;\;\;l_{b}\ll L, \end{array}$$

then $V_{s}\approx V$, and the phase difference $\Delta \phi =\frac{2\pi l_{b}}{L}$ would be very small; therefore,

(A11) $$\begin{array}{c} W_{g_{1}}\left(t\right)\approx W_{g_{2}}\left(t\right). \end{array}$$

As a result, under these conditions, the moving nanochassis problem can be very well approximated by a fixed beam with nearly identical harmonic base excitations at both ends.

## Appendix B. The Values of ${\bar{G}}_{b}^{\alpha \beta }$ and ${\bar{p}}_{b}^{\gamma }$

### Appendix B.1. According to the NDS-Based Model

(A12a)
$$\begin{array}{c} \begin{array}{c} \bar{x}\left(\tau \right)={\left\{{\bar{X}}_{p_{1}}\left(\tau \right),{\bar{X}}_{p_{2}}\left(\tau \right),\ldots ,{\bar{X}}_{p_{NM_{X}}}\left(\tau \right)\right\}}^{T}, \end{array} \end{array}$$

(A12b)
$$\begin{array}{c} \begin{array}{c} \bar{y}\left(\tau \right)={\left\{{\bar{Y}}_{p_{1}}\left(\tau \right),{\bar{Y}}_{p_{2}}\left(\tau \right),\ldots ,{\bar{Y}}_{p_{NM_{Y}}}\left(\tau \right)\right\}}^{T}, \end{array} \end{array}$$

(A12c)
$$\begin{array}{c} \begin{array}{c} {\left[{\bar{G}}_{b}^{uu}\right]}_{ij}=\int_{0}^{1}\left(1+\chi_{0}\right)\;\left(\phi_{i}^{X}\phi_{j}^{X}+\mu^{2}\frac{d\phi_{i}^{X}}{d\eta }\frac{d\phi_{j}^{X}}{d\eta }\right)\;d\eta , \end{array} \end{array}$$

(A12d)
$$\begin{array}{c} \begin{array}{c} {\left[{\bar{G}}_{b}^{ww}\right]}_{ij}=\int_{0}^{1}\left[\begin{array}{c} \left(1+\chi_{0}\right)\;\left(\phi_{i}^{Y}\phi_{j}^{Y}+\mu^{2}\frac{d\phi_{i}^{Y}}{d\eta }\frac{d\phi_{j}^{Y}}{d\eta }\right)\;\\ +\lambda^{-2}\left(1+\chi_{2}\right)\;\left(\frac{d\phi_{i}^{Y}}{d\eta }\frac{d\phi_{j}^{Y}}{d\eta }+\mu^{2}\frac{d^{2}\phi_{i}^{Y}}{d\eta^{2}}\frac{d^{2}\phi_{j}^{Y}}{d\eta^{2}}\right) \end{array}\right]d\eta , \end{array} \end{array}$$

(A12e)
$$\begin{array}{c} \begin{array}{c} {\left\{{\bar{p}}_{b}^{X}\right\}}_{i}\left(\tau \right)=-\int_{0}^{1}\left\{\begin{array}{c} \lambda^{2}\left(1+\chi_{1}\right)\left[\frac{\partial {\bar{X}}_{p}}{\partial \eta }+\frac{1}{2}\left({\left(\frac{\partial {\bar{X}}_{p}}{\partial \eta }\right)}^{2}+{\left(\frac{\partial {\bar{Y}}_{p}}{\partial \eta }\right)}^{2}\right)\right]\left(1+\frac{\partial {\bar{X}}_{p}}{\partial \eta }\right)\frac{d\phi_{i}^{X}}{d\eta }+\;\\ \left(1+\chi_{0}\right)\;\left(2\lambda \beta_{x}\frac{\partial^{2}{\bar{X}}_{p}}{\partial \tau \partial \eta }+{\left(\lambda \beta_{x}\right)}^{2}\frac{\partial^{2}{\bar{X}}_{p}}{\partial \eta^{2}}\right)\;\left(\phi_{i}^{X}-\mu^{2}\frac{d^{2}\phi_{i}^{X}}{d\eta^{2}}\right) \end{array}\right\}d\eta , \end{array} \end{array}$$

(A12f)
$$\begin{array}{c} \begin{array}{c} {\left\{{\bar{p}}_{b}^{Y}\right\}}_{i}\left(\tau \right)=-\int_{0}^{1}\left\{\begin{array}{c} \lambda^{-2}\frac{\partial {\bar{Y}}_{p}}{\partial \eta }\frac{d\phi_{i}^{Y}}{d\eta }\left[\frac{\partial {\bar{X}}_{p}}{\partial \eta }+\frac{1}{2}\left({\left(\frac{\partial {\bar{X}}_{p}}{\partial \eta }\right)}^{2}+{\left(\frac{\partial {\bar{Y}}_{p}}{\partial \eta }\right)}^{2}\right)\right]+\left(1+\chi_{3}\right)\;\frac{\partial^{2}{\bar{Y}}_{p}}{\partial \eta^{2}}\frac{d^{2}\phi_{i}^{Y}}{d\eta^{2}}\;\\ +\left(1+\chi_{0}\right)\;\left(\phi_{i}^{Y}-\mu^{2}\frac{d^{2}\phi_{i}^{Y}}{d\eta^{2}}\right)\left(2\lambda \beta_{x}\frac{\partial^{2}{\bar{Y}}_{p}}{\partial \tau \partial \eta }+{\left(\lambda \beta_{x}\right)}^{2}\frac{\partial^{2}{\bar{Y}}_{p}}{\partial \eta^{2}}\right)\;\\ +\left(1+\chi_{2}\right)\;\lambda^{-2}\;\left(\phi_{i}^{Y}-\mu^{2}\frac{d^{2}\phi_{i}^{Y}}{d\eta^{2}}\right)\;\frac{\partial^{2}}{\partial \eta^{2}}\left[2\lambda \beta_{x}\frac{\partial^{2}{\bar{Y}}_{p}}{\partial \tau \partial \eta }+{\left(\lambda \beta_{x}\right)}^{2}\frac{\partial^{2}{\bar{Y}}_{p}}{\partial \eta^{2}}\right]\;\\ +\left(\chi_{4}+\lambda^{2}\left(1+\chi_{1}\right)\left({\bar{W}}_{g_{2}}\left(\tau \right)-{\bar{W}}_{g_{1}}\left(\tau \right)\right)\right)\;\frac{d\phi_{i}^{Y}}{d\eta }\frac{\partial {\bar{Y}}_{p}}{\partial \eta }+\left(1+\chi_{0}\right)\times \;\\ \phi_{i}^{Y}\left[\left(\left(\frac{d^{2}{\bar{W}}_{g_{2}}}{d\tau^{2}}-\frac{d^{2}{\bar{W}}_{g_{1}}}{d\tau^{2}}\right)\eta +\frac{d^{2}{\bar{W}}_{g_{1}}}{d\tau^{2}}\right)+2\lambda \beta_{x}\;\left(\frac{d{\bar{W}}_{g_{2}}}{d\tau }-\frac{d{\bar{W}}_{g_{1}}}{d\tau }\right)\right] \end{array}\right\}\;d\eta . \end{array} \end{array}$$

### Appendix B.2. According to the NIS-Based Model

(A13a)
$$\begin{array}{c} \begin{array}{c} {\left[{\bar{G}}_{b}^{uu}\right]}_{ij}=\int_{0}^{1}\left(1+\chi_{0}\right)\;\phi_{i}^{X}\phi_{j}^{X}\;d\eta , \end{array} \end{array}$$

(A13b)
$$\begin{array}{c} \begin{array}{c} {\left[{\bar{G}}_{b}^{ww}\right]}_{ij}=\int_{0}^{1}\left\{\begin{array}{c} \left(1+\chi_{0}\right)\;\phi_{i}^{Y}\phi_{j}^{Y}+\lambda^{-2}\left(1+\chi_{21}\right)\;\frac{d\phi_{i}^{Y}}{d\eta }\frac{d\phi_{j}^{Y}}{d\eta }\;\\ +\chi_{22}\;\frac{d^{2}\phi_{i}^{Y}}{d\eta^{2}}\;\left[\int_{0}^{1}{\bar{\alpha }}_{0}\;F(|\eta -\eta^{\prime }\left|;{\bar{l}}_{s}\right)\;\phi_{j}^{Y}\;d\eta^{\prime }\right] \end{array}\right\}d\eta , \end{array} \end{array}$$

(A13c)
$$\begin{array}{c} \begin{array}{c} {\left\{{\bar{p}}_{b}^{X}\right\}}_{i}\left(\tau \right)=-\int_{0}^{1}\left\{\begin{array}{c} \lambda^{2}\left(1+\chi_{1}\right)\frac{d\phi_{i}^{X}}{d\eta }\int_{0}^{1}{\bar{\alpha }}_{0}\;F(|\eta -\eta^{\prime }\left|;{\bar{l}}_{s}\right)\;\left\{\left[\frac{\partial {\bar{X}}_{p}}{\partial \eta }+\frac{\partial {\bar{Y}}_{p}}{\partial \eta }\times \right.\right.\;\\ \left.\left.\left({\bar{W}}_{g_{2}}\left(\tau \right)-{\bar{W}}_{g_{1}}\left(\tau \right)\right)\right]+\frac{1}{2}\left[{\left(\frac{\partial {\bar{X}}_{p}}{\partial \eta }\right)}^{2}+{\left(\frac{\partial {\bar{Y}}_{p}}{\partial \eta }\right)}^{2}\right]\right\}d\eta^{\prime }\;\\ +\left(1+\chi_{0}\right)\;\phi_{i}^{X}\;\left(2\lambda \beta_{x}\frac{\partial^{2}{\bar{X}}_{p}}{\partial \tau \partial \eta }+{\left(\lambda \beta_{x}\right)}^{2}\frac{\partial^{2}{\bar{X}}_{p}}{\partial \eta^{2}}\right) \end{array}\right\}d\eta , \end{array} \end{array}$$

(A13d)
$$\begin{array}{c} \begin{array}{c} {\left\{{\bar{p}}_{b}^{Y}\right\}}_{i}\left(\tau \right)=-\int_{0}^{1}\left\{\begin{array}{c} +\frac{d^{2}\phi_{i}^{Y}}{d\eta^{2}}\;\int_{0}^{1}{\bar{\alpha }}_{0}\;F(|\eta -\eta^{\prime }\left|;{\bar{l}}_{s}\right)\;\left\{\left(1+\chi_{3}\right)\frac{\partial^{2}{\bar{Y}}_{p}}{\partial \eta^{2}}\right.\;\\ \left.+\chi_{22}\left[2\lambda \beta_{x}\frac{\partial^{2}{\bar{Y}}_{p}}{\partial \tau \partial \eta }+{\left(\lambda \beta_{x}\right)}^{2}\;\frac{\partial^{2}{\bar{Y}}_{p}}{\partial \eta^{2}}\right]\right\}d\eta^{\prime }\;\\ +\lambda^{2}\left(1+\chi_{1}\right)\frac{d\phi_{i}^{Y}}{d\eta }\left[\frac{\partial {\bar{Y}}_{p}}{\partial \eta }+\left({\bar{W}}_{g_{2}}\left(\tau \right)-{\bar{W}}_{g_{1}}\left(\tau \right)\right)\right]\;\int_{0}^{1}{\bar{\alpha }}_{0}\;F(|\eta -\eta^{\prime }\left|;{\bar{l}}_{s}\right)\times \;\\ \left\{\left[\frac{\partial {\bar{X}}_{p}}{\partial \eta }+\frac{\partial {\bar{Y}}_{p}}{\partial \eta }\left({\bar{W}}_{g_{2}}\left(\tau \right)-{\bar{W}}_{g_{1}}\left(\tau \right)\right)\right]+\frac{1}{2}\left[{\left(\frac{\partial {\bar{X}}_{p}}{\partial \eta }\right)}^{2}+{\left(\frac{\partial {\bar{Y}}_{p}}{\partial \eta }\right)}^{2}\right]\right\}d\eta^{\prime }\;\\ +\lambda^{-2}\;\left(1+\chi_{21}\right)\;\frac{d\phi_{i}^{Y}}{d\eta }\;\frac{\partial }{\partial \eta }\left[2\lambda \beta_{x}\frac{\partial^{2}{\bar{Y}}_{p}}{\partial \tau \partial \eta }+{\left(\lambda \beta_{x}\right)}^{2}\frac{\partial^{2}{\bar{Y}}_{p}}{\partial \eta^{2}}\right]+\chi_{4}\;\frac{d\phi_{i}^{Y}}{d\eta }\frac{\partial {\bar{Y}}_{p}}{\partial \eta }\;\\ +\left(1+\chi_{0}\right)\;\phi_{i}^{Y}\;\left[2\lambda \beta_{x}\frac{\partial^{2}{\bar{Y}}_{p}}{\partial \tau \partial \eta }+{\left(\lambda \beta_{x}\right)}^{2}\frac{\partial^{2}{\bar{Y}}_{p}}{\partial \eta^{2}}\right]+\left(1+\chi_{0}\right)\times \;\\ \phi_{i}^{Y}\left[\left(\left(\frac{d^{2}{\bar{W}}_{g_{2}}}{d\tau^{2}}-\frac{d^{2}{\bar{W}}_{g_{1}}}{d\tau^{2}}\right)\eta +\frac{d^{2}{\bar{W}}_{g_{1}}}{d\tau^{2}}\right)+2\lambda \beta_{x}\;\left(\frac{d{\bar{W}}_{g_{2}}}{d\tau }-\frac{d{\bar{W}}_{g_{1}}}{d\tau }\right)\right]\;\\ +\chi_{22}\;\int_{0}^{1}{\bar{\alpha }}_{0}\;\frac{\partial^{2}}{\partial \eta^{2}}\;F(|\eta -\eta^{\prime }\left|;{\bar{l}}_{s}\right)\;\left[\left(\frac{d^{2}{\bar{W}}_{g_{2}}}{d\tau^{2}}-\frac{d^{2}{\bar{W}}_{g_{1}}}{d\tau^{2}}\right)\eta +\frac{d^{2}{\bar{W}}_{g_{1}}}{d\tau^{2}}\right]d\eta^{\prime } \end{array}\right\}\;d\eta . \end{array} \end{array}$$

## References

[1] Schliwa M., Woehlke G. (2003). Molecular motors. Nature.

[2] Kay E.R., Leigh D.A., Zerbetto F. (2007). Synthetic molecular motors and mechanical machines. Angew. Chem. Int. Ed..

[3] Kottas G.S., Clarke L.I., Horinek D., Michl J. (2005). Artificial molecular rotors. Chem. Rev..

[4] Michl J., Sykes E.C.H. (2009). Molecular rotors and motors: Recent advances and future challenges. ACS Nano.

[5] Balzani V., Credi A., Venturi M. (2007). Molecular devices and machines. Nano Today.

[6] Badjic J.D., Balzani V., Credi A., Silvi S., Stoddart J.F. (2004). A molecular elevator. Science.

[7] Badjic J.D., Ronconi C.M., Stoddart J.F., Balzani V., Silvi S., Credi A. (2006). Operating molecular elevators. J. Am. Chem. Soc..

[8] Shirai Y., Osgood A.J., Zhao Y., Kelly K.F., Tour J.M. (2005). Directional control in thermally driven single-molecule nanocars. Nano Lett..

[9] Morin J.F., Shirai Y., Tour J.M. (2006). En route to a motorized nanocar. Org. Lett..

[10] Morin J.F., Sasaki T., Shirai Y., Guerrero J.M., Tour J.M. (2007). Synthetic routes toward carborane-wheeled nanocars. J. Org. Chem..

[11] Vives G., Tour J.M. (2009). Synthesis of a nanocar with organometallic wheels. Tetrahedron Lett..

[12] Zhao Z., Sun L., Zhao X., Liu Y. (2026). Modeling and dynamics analysis of a rigid-flexible coupled satellite. Appl. Math. Model..

[13] Rojas A., Sun L., Zhao X., Liu Y. (2026). Quasi-symplectic ADRC hybridization to stabilize a flexible truss for a space telescope. Aerosp. Sci. Technol..

[14] Yue T., Wang T. (2025). New Datko-type results for the uniform exponential instability in mean of cocycles. Dyn. Syst..

[15] Fu W., Wang X., Liu Y. (2015). Impact-induced soft-tissue vibrations associate with muscle activation in human landing movements: An accelerometry and EMG evaluation. Technol. Health Care.

[16] Qin X., Zhao P., Yang W., Cai Y., Tang H., Cheng N. (2026). Noise barriers with periodic arrays of non-uniform resonant cavities: Tailored design for low-frequency noise from heavy-duty highway traffic. Transp. Res. Part D Transp. Environ..

[17] Lv C., Ji Z., Yang T., Zhao H., Zhang H. (2025). Numerical simulation of deformation and breakage of compound droplet in air flow. Phys. Fluids.

[18] Fu W.J., Ruan M.F. The role of footwear on impact forces and soft tissue vibrations during active and passive landings. Proceedings of the 6th World Congress of Biomechanics (WCB 2010).

[19] Fu W., Liu Y., Zhang S. (2013). Effects of footwear on impact forces and soft tissue vibrations during drop jumps and unanticipated drop landings. Int. J. Sport. Med..

[20] Rofooei F.R., Enshaeian A., Nikkhoo A. (2017). Dynamic response of geometrically nonlinear, elastic rectangular plates under a moving mass loading by inclusion of all inertial components. J. Sound Vib..

[21] Jahangiri A., Attari N.K., Nikkhoo A., Waezi Z. (2020). Nonlinear dynamic response of an Euler–Bernoulli beam under a moving mass–spring with large oscillations. Arch. Appl. Mech..

[22] Alibakhshi A., Dastjerdi S., Malikan M., Eremeyev V.A. (2021). Nonlinear free and forced vibrations of a hyperelastic micro/nanobeam considering strain stiffening effect. Nanomaterials.

[23] Mirjavadi S.S., Forsat M., Nikookar M., Barati M.R., Hamouda A.M. (2019). Nonlinear forced vibrations of sandwich smart nanobeams with two-phase piezo-magnetic face sheets. Eur. Phys. J. Plus.

[24] Eringen A.C. (1972). Nonlocal polar elastic continua. Int. J. Eng. Sci..

[25] Eringen A.C., Edelen D.G.B. (1972). On nonlocal elasticity. Int. J. Eng. Sci..

[26] Eringen A.C. (1972). Linear theory of nonlocal elasticity and dispersion of plane waves. Int. J. Eng. Sci..

[27] Eringen A.C. (2002). Nonlocal Continuum Field Theories.

[28] Peddieson J., Buchanan G.R., McNitt R.P. (2003). Application of nonlocal continuum models to nanotechnology. Int. J. Eng. Sci..

[29] Sudak L.J. (2003). Column buckling of multiwalled carbon nanotubes using nonlocal continuum mechanics. J. Appl. Phys..

[30] Thai H.T. (2012). A nonlocal beam theory for bending, buckling, and vibration of nanobeams. Int. J. Eng. Sci..

[31] Liu C., Ke L.L., Wang Y.S., Yang J., Kitipornchai S. (2014). Buckling and post-buckling of size-dependent piezoelectric Timoshenko nanobeams subject to thermo-electro-mechanical loadings. Int. J. Struct. Stab. Dyn..

[32] Kiani K. (2015). Nonlocal and shear effects on column buckling of single-layered membranes from stocky single-walled carbon nanotubes. Compos. Part B Eng..

[33] Mercan K., Civalek O. (2016). DSC method for buckling analysis of boron nitride nanotube (BNNT) surrounded by an elastic matrix. Compos. Struct..

[34] Shen H.S. (2010). Nonlocal shear deformable shell model for postbuckling of axially compressed microtubules embedded in an elastic medium. Biomech. Model. Mechanobiol..

[35] Kiani K. (2017). Postbuckling scrutiny of highly deformable nanobeams: A novel exact nonlocal-surface energy-based model. J. Phys. Chem. Solids.

[36] Dai H.L., Ceballes S., Abdelkefi A., Hong Y.Z., Wang L. (2018). Exact modes for post-buckling characteristics of nonlocal nanobeams in a longitudinal magnetic field. Appl. Math. Model..

[37] Murmu T., Adhikari S. (2011). Nonlocal vibration of bonded double-nanoplate-systems. Compos. Part B Eng..

[38] Wang Q. (2005). Wave propagation in carbon nanotubes via nonlocal continuum mechanics. J. Appl. Phys..

[39] Wang C.M., Zhang Y.Y., He X.Q. (2007). Vibration of nonlocal Timoshenko beams. Nanotechnology.

[40] Nikkhoo A., Zolfaghari S., Kiani K. (2017). A simplified-nonlocal model for transverse vibration of nanotubes acted upon by a moving nanoparticle. J. Braz. Soc. Mech. Sci. Eng..

[41] Ma X., Roshan M., Kiani K., Nikkhoo A. (2023). Dynamic response of an elastic tube-like nanostructure embedded in a vibrating medium and under the action of moving nano-objects. Symmetry.

[42] Xie B., Kiani K. (2021). Elasto-dynamics of doubly mislocated stocky beam-like nanostructures immersed in inclined magnetic fields using nonlocal continuum mechanics. Eur. Phys. J. Plus.

[43] Shen L.E., Shen H.S., Zhang C.L. (2010). Nonlocal plate model for nonlinear vibration of single layer graphene sheets in thermal environments. Comput. Mater. Sci..

[44] Ke L.L., Wang Y.S., Wang Z.D. (2012). Nonlinear vibration of the piezoelectric nanobeams based on the nonlocal theory. Compos. Struct..

[45] Arani A.G., Vossough H., Kolahchi R., Barzoki A.M. (2012). Electro-thermo nonlocal nonlinear vibration in an embedded polymeric piezoelectric micro plate reinforced by DWBNNTs using DQM. J. Mech. Sci. Technol..

[46] Ebrahimi F., Hosseini S.H.S. (2016). Thermal effects on nonlinear vibration behavior of viscoelastic nanosize plates. J. Therm. Stress..

[47] Eringen A.C., Wegner J.L. (2003). Nonlocal continuum field theories. Appl. Mech. Rev..

[48] Emmrich E., Weckner O. (2007). Analysis and numerical approximation of an integro-differential equation modeling non-local effects in linear elasticity. Math. Mech. Solids.

[49] Yu Y.J., Tian X.G., Liu X.R. (2015). Size-dependent generalized thermoelasticity using Eringen’s nonlocal model. Eur. J. Mech.-A/Solids.

[50] Kiani K. (2016). Free vibration of in-plane-aligned membranes of single-walled carbon nanotubes in the presence of in-plane-unidirectional magnetic fields. J. Vib. Control.

[51] Bian P.L., Qing H. (2021). On bending consistency of Timoshenko beam using differential and integral nonlocal strain gradient models. Zamm-J. Appl. Math. Mech. Angew. Math. Mech..

[52] Wang X., Chen Y., Yu J. (2023). Wave propagation in viscoelastic functionally graded nanoplates: Comparison of the integral and differential nonlocal models. Acta Mech. Solida Sin..

[53] Kiani K. (2011). Nonlocal continuum-based modeling of a nanoplate subjected to a moving nanoparticle. Part II: Parametric studies. Phys. E Low.-Dimens. Syst. Nanostructures.

[54] Wu C.P., Hu H.X. (2021). A review of dynamic analyses of single-and multi-layered graphene sheets/nanoplates using various nonlocal continuum mechanics-based plate theories. Acta Mech..

[55] Eptaimeros K.G., Koutsoumaris C.C., Tsamasphyros G.J. (2016). Nonlocal integral approach to the dynamical response of nanobeams. Int. J. Mech. Sci..

[56] Kiani K. (2016). Nonlocal-integro-differential modeling of vibration of elastically supported nanorods. Phys. E Low-Dimens. Syst. Nanostructures.

[57] Fernandez-Saez J., Zaera R., Loya J.A., Reddy J.N. (2016). Bending of Euler–Bernoulli beams using Eringen’s integral formulation: A paradox resolved. Int. J. Eng. Sci..

[58] Romano G., Barretta R., Diaco M., de Sciarra F.M. (2017). Constitutive boundary conditions and paradoxes in nonlocal elastic nanobeams. Int. J. Mech. Sci..

[59] Apuzzo A., Barretta R., Luciano R., de Sciarra F.M., Penna R. (2017). Free vibrations of Bernoulli-Euler nano-beams by the stress-driven nonlocal integral model. Compos. Part B Eng..

[60] Lim C.Y., Li C., Yu J.L. (2010). Dynamic behaviour of axially moving nanobeams based on nonlocal elasticity approach. Acta Mech. Sin..

[61] Kiani K. (2014). Longitudinal and transverse instabilities of moving nanoscale beam-like structures made of functionally graded materials. Compos. Struct..

[62] Li C. (2017). Nonlocal thermo-electro-mechanical coupling vibrations of axially moving piezoelectric nanobeams. Mech. Based Des. Struct. Mach..

[63] Wang J., Shen H., Zhang B., Liu J., Zhang Y. (2018). Complex modal analysis of transverse free vibrations for axially moving nanobeams based on the nonlocal strain gradient theory. Phys. E Low-Dimens. Syst. Nanostructures.

[64] Mokhtari A., Mirdamadi H.R., Ghayour M., Sarvestan V. (2016). Time/wave domain analysis for axially moving pre-stressed nanobeam by wavelet-based spectral element method. Int. J. Mech. Sci..

[65] Guo S., He Y., Liu D., Lei J., Li Z. (2018). Dynamic transverse vibration characteristics and vibro-buckling analyses of axially moving and rotating nanobeams based on nonlocal strain gradient theory. Microsyst. Technol..

[66] Kiani K., Efazati M. (2020). Nonlocal vibrations and instability of three-dimensionally accelerated moving nanocables. Phys. Scr..

[67] Kiani K., Efazati M. (2021). Three-dimensional nonlocal-surface energy-based statics, dynamics, and divergence instability of movable cable-like nanostructures with arbitrary translational motion. Arch. Appl. Mech..

[68] Gurtin M.E., Murdoch A.I. (1976). Effect of surface stress on wave propagation in solids. J. Appl. Phys..

[69] Gurtin M.E., Murdoch A.I. (1978). Surface stress in solids. Int. J. Solids Struct..

[70] Miller R.E., Shenoy V.B. (2000). Size-dependent elastic properties of nanosized structural elements. Nanotechnology.

[71] Shenoy V.B. (2005). Atomistic calculations of elastic properties of metallic fcc crystal surfaces. Phys. Rev. B.

[72] Yan Z., Jiang L.Y. (2011). The vibrational and buckling behaviors of piezoelectric nanobeams with surface effects. Nanotechnology.

[73] Yan Z., Jiang L.Y. (2012). Surface effects on the vibration and buckling of piezoelectric nanoplates. Europhys. Lett..

[74] Hosseini-Hashemi S., Nahas I., Fakher M., Nazemnezhad R. (2014). Surface effects on free vibration of piezoelectric functionally graded nanobeams using nonlocal elasticity. Acta Mech..

[75] Kiani K. (2014). Surface effect on free transverse vibrations and dynamic instability of current-carrying nanowires in the presence of a longitudinal magnetic field. Phys. Lett. A.

[76] Ansari R., Gholami R. (2016). Surface effect on the large amplitude periodic forced vibration of first-order shear deformable rectangular nanoplates with various edge supports. Acta Astronaut..

[77] Dai H.L., Zhao D.M., Zou J.J., Wang L. (2016). Surface effect on the nonlinear forced vibration of cantilevered nanobeams. Phys. E Low.-Dimens. Syst. Nanostructures.

[78] Askari H., Esmailzadeh E. (2017). Forced vibration of fluid conveying carbon nanotubes considering thermal effect and nonlinear foundations. Compos. Part B Eng..

[79] Sharabiani P.A., Yazdi M.R.H. (2013). Nonlinear free vibrations of functionally graded nanobeams with surface effects. Compos. Part B Eng..

[80] Fu Y., Zhang J., Jiang Y. (2010). Influences of the surface energies on the nonlinear static and dynamic behaviors of nanobeams. Phys. E Low.-Dimens. Syst. Nanostructures.

[81] Ding P., Xu R., Wang L., Gao Z., Ge X., Wen M. (2026). Fractional derivative viscoelastic models based on L_2−1σ_ formula: Modelling and numerical application. Comput. Geotech..

[82] Zhao J., Zhu H., Zhang Z., Feng M., Yu H., Li Y. (2025). A fractional-order SSIM-based gaussian loss with long-range memory for dense VSLAM. Fractal Fract..

[83] Zhang H., Zhao S., Zhang Q., Deng C., Zhao C., Wang X., Malashicheva A., Wang Y., Qiu J., Wang G. (2026). Surface charge-determined protein coronas of nanoparticles control endothelial cells uptake under low magnitude shear stress. Exploration.

[84] Li X., Lv J., Ni W., Chen C., Zhao M., Wang X., Li M., Lei G. (2025). Hydrogen ion-induced surface damage of copper grids in RF ion sources for fusion NBI. Nucl. Mater. Energy.

[85] Kiani K. (2019). Divergence and flutter instabilities of nanobeams in moving state accounting for surface and shear effects. Comput. Math. Appl..

[86] Aichun L., Kiani K. (2020). Bilaterally flexural vibrations and instabilities of moving piezoelectric nanowires with surface effect. Eur. Phys. J. Plus.

[87] Chen Z., Wang F., Zhang H., Chen Z., He Q. (2025). Performance and safety evaluation of rack vehicle in mountainous regions under pulsating wind load. Mech. Based Des. Struct. Mach..

[88] Wu Y., Fan L., Li B., Wei Y., Gu Y., Wang J. (2025). Establishment of a Modelica model for flow metering valve controlled pump and analysis on delivery and leakage characteristics. Energy.

[89] Zhao Z., Wang J., Shiau J., Luo H., Yu D. (2025). Probabilistic analysis of pile-reinforced slopes considering anisotropic spatial soil properties. Int. J. Numer. Anal. Methods Geomech..

[90] Wu M., Liu Z., Wang H., Zhou H., Wang X. (2025). Cyclic fatigue effect on mechanical property change of hot dry rock rocks in wellbores of enhanced geothermal systems. Int. J. Rock Mech. Min. Sci..

[91] Ghadiri M., Shafiei N., Akbarshahi A. (2016). Influence of thermal and surface effects on vibration behavior of nonlocal rotating Timoshenko nanobeam. Appl. Phys. A.

[92] Pourkiaee S.M., Khadem S.E., Shahgholi M. (2017). Nonlinear vibration and stability analysis of an electrically actuated piezoelectric nanobeam considering surface effects and intermolecular interactions. J. Vib. Control.

[93] Wang D.H., Wang G.F. (2011). Surface effects on the vibration and buckling of double-nanobeam-systems. J. Nanomater..

[94] On B.B., Altus E. (2011). Effects of local surface residual stresses on the near resonance vibrations of nano-beams. J. Sound Vib..

[95] Tunvir K., Ru C.Q., Mioduchowski A. (2012). Effect of cross-sectional shape on thermoelastic dissipation of micro/nano elastic beams. Int. J. Mech. Sci..

[96] Dai J., Liu H., Fang W., Wang L., Pu Y., Jiang F. (2005). Comparisons of structural and optical properties of ZnO films grown on (0 0 0 1) sapphire and GaN/(0 0 0 1) sapphire template by atmospheric-pressure MOCVD. Mater. Sci. Eng. B.

[97] Dai J.N., Han X.Y., Wu Z.H., Fang Y.Y., Xiong H., Tian Y., Yu C.H., He Q.H., Chen C.Q. (2011). Effects of growth temperature on properties of nonpolar a-plane ZnO films on GaN templates by pulsed laser deposition. J. Electron. Mater..

[98] Zhao Y., Miranda Cortez P.A., Zou Z., Mei Y., Jian P., Liu W., Wei Y., Zhang B., Wu F., Chen C. (2021). AlGaN-based deep ultraviolet vertical-cavity surface-emitting lasers on a flexible substrate. Opt. Lett..

[99] Tan S., Jian P., Shan M., Zhao Y., Zheng Z., Yang Y., Zeng Y., Xu D., Chen Z., Chen C. (2024). Tunable structured AlGaN-based nanoporous distributed Bragg reflectors for light-coupling enhancement in monolayer MoS_2_. Opt. Laser Technol..

[100] Gao Z., Wei Y., Liu W., Zhao Y., Li Z., Liang Z., Zeng Z.L., Wu F., Peng Y., Dai J. (2026). High-temperature annealing-assisted high-quality sp2-BN film by MOCVD for vacuum ultraviolet detectors. Cryst. Growth Des..

[101] Dai J.N., Han X.Y., Wu Z.H., Yu C.H., Xiang R.F., He Q.H., Gao Y.H., Chen C.Q., Xiao X.H., Peng T.C. (2010). Growth of non-polar ZnO films on a-GaN/r-Al2O3 templates by radio-frequency magnetron sputtering. J. Alloys Compd..

[102] Xu J., Ding T., Wang J., Zhang J., Wang S., Chen C., Fang Y., Wu Z., Huo K., Dai J. (2015). Tungsten oxide nanofibers self-assembled mesoscopic microspheres as high-performance electrodes for supercapacitor. Electrochim. Acta.

[103] Zeng Y., Peng M., Xing Y., Zeng X., Liu Z., Liu W., Zhao Y., Li Z., Luo Y., Wei Y. (2026). Flexible self-powered photodetector enabled by TeSeO amorphous-crystalline hybrid. Laser Photonics Rev..

[104] Tian K., Chen Q., Chu C., Fang M., Li L., Zhang Y., Bi W., Chen C., Zhang Z.H., Dai J. (2018). Investigations on AlGaN-based deep-ultraviolet light-emitting diodes with Si-doped quantum barriers of different doping concentrations. Phys. Status Solidi.

[105] Zhao Y., Deng L., Wu F., Zheng Z., Jian P., Liu W., Chen Z., Tan S., Peng M., Guo W. (2024). Low-threshold AlGaN-based deep ultraviolet laser enabled by a nanoporous cladding layer. Opt. Lett..

[106] Wei Y., Shan M., Gao Z., Zhao Y., Li Z., Chen Z., Zeng Y., Liang Z., Tian X., Peng Y. (2025). Light extraction efficiency enhancement of deep ultraviolet light-emitting diodes using wafer-scale SiO_2_-based patterned dielectric nanostructures. Opt. Lett..

[107] Zhang Y., Wang Q., Sun H. (2016). Effect of sapphire substrate on the localized surface plasmon resonance of aluminum triangular nanoparticles. Opt. Commun..

[108] Chen Z., Zhang S., Zhao Y., Wu Z., Chen M., Zeng Y., Gao Z., Wei Y., Li Z., Liang Z. (2025). Enhancement of light extraction efficiency in algan-based deep ultraviolet light-emitting diodes using cooperative scattering structures on the n-AlGaN layer. Laser Photonics Rev..

[109] Zhang J., Dai J., Zhu L., Chen C., Wan Q. (2014). Laterally coupled IZO-based transistors on free-standing proton conducting chitosan membranes. IEEE Electron Device Lett..

[110] Tian Y., Li J., Xiong H., Dai J. (2012). Controlled synthesis of ZnO hollow microspheres via precursor-template method and its gas sensing property. Appl. Surf. Sci..

[111] Nikkhoo A., Rofooei F.R., Shadnam M.R. (2007). Dynamic behavior and modal control of beams under moving mass. J. Sound Vib..

[112] Homaeinezhad M.R., Abbasi Gavari M. (2023). Feedback control of actuation-constrained moving structure carrying Timoshenko beam. Int. J. Robust Nonlinear Control.

[113] Nikkhoo A. (2014). Investigating the behavior of smart thin beams with piezoelectric actuators under dynamic loads. Mech. Syst. Signal Process..

[114] Sun L., Zhao Z., Zhao X., Liu Y. (2026). Adaptive attitude maneuver control of a rigid-flexible satellite based on deep reinforcement learning. IEEE Trans. Aerosp. Electron. Syst..

[115] Yu D., Wen M., Ge X., Zhang Y., Wan J., Lou S. (2026). Semianalytical solution for one-dimensional consolidation of unsaturated–saturated soil foundations based on the interfacial flow contact resistance model. Int. J. Numer. Anal. Methods Geomech..

[116] Zhou H., Liu Z., Shao J., Shen W., Hamdi E. (2026). Effects of stress direction and magnitude on strength and failure of weakly anisotropic sandstone under true triaxial compression. Rock Mech. Rock Eng..

[117] Li Z., Zhao J., Ding Y., Zhang H., Chen Z., Wu Z., Wei Y., Wu F., Chen C., Peng Y. (2025). Unique multilayer gradient design of *Al*_2_O_3_ doping phosphor-in-glass film enabling high-luminance laser lighting. Laser Photonics Rev..

[118] Ding T., Tian Y., Dai J., Chen C. (2015). Building one-dimensional Bi_2_S_3_ nanorods as enhanced photoresponding materials for photodetectors. Front. Optoelectron..

[119] Tian Y., Chen H., Zhu X., Zheng G., Dai J. (2013). Selective growth and characterization of ZnO nanorods assembled a hexagonal pattern on H_2_-decomposed GaN epilayer. Front. Optoelectron..

[120] Dai J., Su H., Wang L., Pu Y., Fang W., Jiang F. (2006). Properties of ZnO films grown on (0 0 0 1) sapphire substrate using H_2_O and N_2_O as O precursors by atmospheric pressure MOCVD. J. Cryst. Growth.

[121] Wang Z., Zhang X., Li W., Feng Z. (2025). Real-time UUV obstacle avoidance through flexible steering technology based on improved soft actor-critic framework. IEEE Trans. Instrum. Meas..

[122] Vives G., Tour J.M. (2009). Synthesis of single-molecule nanocars. Acc. Chem. Res..

[123] Kudernac T., Ruangsupapichat N., Parschau M., Maciá B., Katsonis N., Harutyunyan S.R., Ernst K.H., Feringa B.L. (2011). Electrically driven directional motion of a four-wheeled molecule on a metal surface. Nature.

[124] Rapenne G., Joachim C. (2017). The first nanocar race. Nat. Rev. Mater..

[125] Kassem S., van Leeuwen T., Lubbe A.S., Wilson M.R., Feringa B.L., Leigh D.A. (2017). Artificial molecular motors. Chem. Soc. Rev..

[126] Wang G.F., Feng X.Q. (2009). Timoshenko beam model for buckling and vibration of nanowires with surface effects. J. Phys. D Appl. Phys..

[127] He J., Lilley C.M. (2008). Surface effect on the elastic behavior of static bending nanowires. Nano Lett..

[128] Martinez A. (2005). Bonding interactions of metal clusters [Mn (M = Cu, Ag, Au; n = 1–4)] with ammonia. Are the metal clusters adequate as a model of surfaces?. J. Braz. Chem. Soc..

[129] Dubiel M., Haug J., Kruth H., Hofmeister H., Seifert Y. (2009). Temperature dependence of EXAFS cumulants of Ag nanoparticles in glass. J. Phys. Conf. Ser..

[130] Duan Y.H., Wang C.M., Zhang Y.Y. (2007). Calibration of nonlocal scaling effect parameter for free vibration of carbon nanotubes by molecular dynamics. J. Appl. Phys..

[131] Ghavanloo E., Fazelzadeh S.A. (2016). Evaluation of nonlocal parameter for single-walled carbon nanotubes with arbitrary chirality. Meccanica.

