# Supplementary material for: Nonlinear Vibrations and Potential Instabilities of a Nanochassis Traveling a Route with Arbitrarily Tiny Irregularities
Source: Nanomaterials (Basel). 2026 Jun 18;16(12):768. doi: 10.3390/nano16120768 (PMC13304772; doi:10.3390/nano16120768)
Supplement: Supplementary file 1 [file nanomaterials-16-00768-s001.zip › nanomaterials-4199778-supplementary.pdf]

Supplementary materials:

S.1. Evaluation of $\dfrac{\boldsymbol{\partial}\widetilde{\mathbf{p}}}{\boldsymbol{\partial}\bar{\mathbf{q}}}$ for the NDS-based model

The consisting submatrices of $\dfrac{\partial\widetilde{\mathbf{p}}}{\partial\bar{\mathbf{q}}}$ are as,

$$\widetilde{\mathbf{p}}_{,\bar{\mathbf{q}}} = \begin{bmatrix} \dfrac{\partial\bar{\mathbf{p}}}{\partial\bar{\mathbf{y}}} & \dfrac{\partial\bar{\mathbf{p}}}{\partial\bar{\mathbf{x}}} \\ \mathbf{I} & \mathbf{0} \end{bmatrix}; \quad \frac{\partial\bar{\mathbf{p}}}{\partial\bar{\mathbf{x}}} = \begin{bmatrix} \dfrac{\partial\bar{\mathbf{p}}_b^{\mathcal{X}}}{\partial\bar{\mathbf{x}}} & \dfrac{\partial\bar{\mathbf{p}}_b^{\mathcal{X}}}{\partial\bar{\mathbf{y}}} \\ \dfrac{\partial\bar{\mathbf{p}}_b^{\mathcal{Y}}}{\partial\bar{\mathbf{x}}} & \dfrac{\partial\bar{\mathbf{p}}_b^{\mathcal{Y}}}{\partial\bar{\mathbf{y}}} \end{bmatrix}, \quad \frac{\partial\bar{\mathbf{p}}}{\partial\bar{\mathbf{y}}} = \begin{bmatrix} \dfrac{\partial\bar{\mathbf{p}}_b^{\mathcal{X}}}{\partial\bar{\mathbf{x}}_{,\tau}} & \dfrac{\partial\bar{\mathbf{p}}_b^{\mathcal{X}}}{\partial\bar{\mathbf{y}}_{,\tau}} \\ \dfrac{\partial\bar{\mathbf{p}}_b^{\mathcal{Y}}}{\partial\bar{\mathbf{x}}_{,\tau}} & \dfrac{\partial\bar{\mathbf{p}}_b^{\mathcal{Y}}}{\partial\bar{\mathbf{y}}_{,\tau}} \end{bmatrix}, \tag{1}$$

where the elements of the submatrices of $\dfrac{\partial\bar{\mathbf{p}}}{\partial\bar{\mathbf{x}}}$ and $\dfrac{\partial\bar{\mathbf{p}}}{\partial\bar{\mathbf{y}}}$ are given by:

$$\left[\frac{\partial\bar{\mathbf{p}}_b^{\mathcal{X}}}{\partial\bar{\mathbf{x}}}\right]_{ij} = -\int_0^1 \begin{bmatrix} \lambda^2(1+\chi_1)\left(1+3\dfrac{\partial\bar{\mathcal{X}}_p}{\partial\eta}+0.5\left(\dfrac{\partial\bar{\mathcal{Y}}_p}{\partial\eta}\right)^2\right)\dfrac{\mathrm{d}\phi_i^{\mathcal{X}}}{\mathrm{d}\eta}\dfrac{\mathrm{d}\phi_j^{\mathcal{X}}}{\mathrm{d}\eta} \\ +(1+\chi_o)\left(\lambda\beta_x\right)^2\left(\phi_i^{\mathcal{X}}-\mu^2\dfrac{\mathrm{d}^2\phi_i^{\mathcal{X}}}{\mathrm{d}\eta^2}\right)\dfrac{\mathrm{d}^2\phi_j^{\mathcal{X}}}{\mathrm{d}\eta^2} \end{bmatrix} \mathrm{d}\eta, \tag{2a}$$

$$\left[\frac{\partial\bar{\mathbf{p}}_b^{\mathcal{X}}}{\partial\bar{\mathbf{y}}}\right]_{ij} = -\int_0^1 \lambda^2(1+\chi_1)\frac{\mathrm{d}\phi_i^{\mathcal{X}}}{\mathrm{d}\eta}\left(\frac{\partial\bar{\mathcal{Y}}_p}{\partial\eta}\frac{\mathrm{d}\phi_j^{\mathcal{Y}}}{\mathrm{d}\eta}+3\lambda^{-2}\frac{\partial^2\bar{\mathcal{Y}}_p}{\partial\eta^2}\frac{\mathrm{d}^2\phi_j^{\mathcal{Y}}}{\mathrm{d}\eta^2}\right)\left(1+\frac{\partial\bar{\mathcal{X}}_p}{\partial\eta}\right)\mathrm{d}\eta, \tag{2b}$$

$$\left[\frac{\partial\bar{\mathbf{p}}_b^{\mathcal{Y}}}{\partial\bar{\mathbf{x}}}\right]_{ij} = -\int_0^1 \frac{\mathrm{d}\phi_j^{\mathcal{X}}}{\mathrm{d}\eta}\left(\frac{\partial^2\bar{\mathcal{Y}}_p}{\partial\eta^2}\frac{\mathrm{d}^2\phi_j^{\mathcal{Y}}}{\mathrm{d}\eta^2}+\lambda^{-2}\frac{\partial\bar{\mathcal{Y}}_p}{\partial\eta}\frac{\mathrm{d}\phi_j^{\mathcal{Y}}}{\mathrm{d}\eta}\right)\left(1+\frac{\partial\bar{\mathcal{X}}_p}{\partial\eta}\right)\mathrm{d}\eta, \tag{2c}$$

$$\left[\frac{\partial\bar{\mathbf{p}}_b^{\mathcal{Y}}}{\partial\bar{\mathbf{y}}}\right]_{ij} = -\int_0^1 \left\{ \begin{array}{l} \begin{bmatrix} \lambda^{-2}\left(\dfrac{\partial\bar{\mathcal{X}}_p}{\partial\eta}+0.5\left(\dfrac{\partial\bar{\mathcal{X}}_p}{\partial\eta}\right)^2+1.5\left(\dfrac{\partial\bar{\mathcal{Y}}_p}{\partial\eta}\right)^2+0.5\dfrac{\partial^2\bar{\mathcal{Y}}_p}{\partial\eta^2}\right) \\ +\left(\chi_4+\lambda^2(1+\chi_1)\left(\bar{\mathcal{W}}_{g_2}(\tau)-\bar{\mathcal{W}}_{g_1}(\tau)\right)\right) \end{bmatrix}\dfrac{\mathrm{d}\phi_i^{\mathcal{Y}}}{\mathrm{d}\eta}\dfrac{\mathrm{d}\phi_j^{\mathcal{Y}}}{\mathrm{d}\eta} \\ +\left[1+\chi_3+\dfrac{\partial\bar{\mathcal{X}}_p}{\partial\eta}+0.5\left(\left(\dfrac{\partial\bar{\mathcal{X}}_p}{\partial\eta}\right)^2+\left(\dfrac{\partial\bar{\mathcal{Y}}_p}{\partial\eta}\right)^2\right)\right]\dfrac{\mathrm{d}^2\phi_i^{\mathcal{Y}}}{\mathrm{d}\eta^2}\dfrac{\mathrm{d}^2\phi_j^{\mathcal{Y}}}{\mathrm{d}\eta^2} \\ +\left(\lambda\beta_x\right)^2\left(\phi_i^{\mathcal{Y}}-\mu^2\dfrac{\mathrm{d}^2\phi_i^{\mathcal{Y}}}{\mathrm{d}\eta^2}\right)\left[(1+\chi_0)\dfrac{\mathrm{d}^2\phi_j^{\mathcal{Y}}}{\mathrm{d}\eta^2}+(1+\chi_2)\lambda^{-2}\dfrac{\mathrm{d}^4\phi_j^{\mathcal{Y}}}{\mathrm{d}\eta^4}\right] \end{array} \right\} \mathrm{d}\eta, \tag{2d}$$

$$\left[\frac{\partial\bar{\mathbf{p}}_b^{\mathcal{X}}}{\partial\bar{\mathbf{x}}_{,\tau}}\right]_{ij} = -2\lambda\beta_x\left(1+\chi_0\right)\int_0^1\left(\phi_i^{\mathcal{X}}-\mu^2\frac{\mathrm{d}^2\phi_i^{\mathcal{X}}}{\mathrm{d}\eta^2}\right)\frac{\mathrm{d}\phi_j^{\mathcal{X}}}{\mathrm{d}\eta}\mathrm{d}\eta, \tag{2e}$$

$$\left[\frac{\partial\bar{\mathbf{p}}_b^{\mathcal{Y}}}{\partial\bar{\mathbf{y}}_{,\tau}}\right]_{ij} = -2\lambda\beta_x\int_0^1\left(\phi_i^{\mathcal{Y}}-\mu^2\frac{\mathrm{d}^2\phi_i^{\mathcal{Y}}}{\mathrm{d}\eta^2}\right)\left[(1+\chi_0)\frac{\mathrm{d}\phi_j^{\mathcal{Y}}}{\mathrm{d}\eta}+\lambda^{-2}\left(1+\chi_2\right)\frac{\mathrm{d}^3\phi_j^{\mathcal{Y}}}{\mathrm{d}\eta^3}\right]\mathrm{d}\eta. \tag{2f}$$

S.2. Evaluation of $\dfrac{\partial \widetilde{\mathbf{p}}}{\partial \bar{\mathbf{q}}}$ for the NIS-based model

Using Eqs. (A.2c) and (A.2d), the nonvanishing elements of the submatrices $\dfrac{\partial \bar{\mathbf{p}}}{\partial \bar{\mathbf{x}}}$ and $\dfrac{\partial \bar{\mathbf{p}}}{\partial \bar{\mathbf{y}}}$ are calculated as follows:

$$\left[\frac{\partial \bar{\mathbf{p}}_b^{\mathcal{X}}}{\partial \bar{\mathbf{x}}}\right]_{ij} = -\int_0^1 \left[\begin{array}{l} (1+\chi_0)(\lambda\beta_x)^2\,\phi_i^{\mathcal{X}}\dfrac{\mathrm{d}^2\phi_j^{\mathcal{X}}}{\mathrm{d}\eta^2} + \lambda^2(1+\chi_1)\dfrac{\mathrm{d}\phi_i^{\mathcal{X}}}{\mathrm{d}\eta}\times \\ \displaystyle\int_0^1 \bar{\alpha}_0\,\mathcal{F}(|\eta-\eta'|;\bar{l}_s)\left(1+\frac{\partial \bar{\mathcal{X}}_p}{\partial \eta}\right)\frac{\mathrm{d}\phi_j^{\mathcal{X}}}{\mathrm{d}\eta}\,\mathrm{d}\eta' \end{array}\right]\mathrm{d}\eta, \tag{3a}$$

$$\left[\frac{\partial \bar{\mathbf{p}}_b^{\mathcal{X}}}{\partial \bar{\mathbf{y}}}\right]_{ij} = -\int_0^1 \left\{\begin{array}{l} \lambda^2(1+\chi_1)\,\dfrac{\mathrm{d}\phi_i^{\mathcal{X}}}{\mathrm{d}\eta}\,\left\{\displaystyle\int_0^1 \bar{\alpha}_0\,\mathcal{F}(|\eta-\eta'|;\bar{l}_s)\times\right. \\ \left.\left[\dfrac{\partial \bar{\mathcal{Y}}_p}{\partial \eta} + \left(\bar{\mathcal{W}}_{g_2}(\tau) - \bar{\mathcal{W}}_{g_1}(\tau)\right)\right]\dfrac{\mathrm{d}\phi_j^{\mathcal{Y}}}{\mathrm{d}\eta}\,\mathrm{d}\eta'\right\} \end{array}\right\}\mathrm{d}\eta, \tag{3b}$$

$$\left[\frac{\partial \bar{\mathbf{p}}_b^{\mathcal{Y}}}{\partial \bar{\mathbf{x}}}\right]_{ij} = -\int_0^1 \left\{\begin{array}{l} \lambda^2(1+\chi_1)\,\dfrac{\mathrm{d}\phi_i^{\mathcal{Y}}}{\mathrm{d}\eta}\,\left[\dfrac{\partial \bar{\mathcal{Y}}_p}{\partial \eta} + \left(\bar{\mathcal{W}}_{g_2}(\tau) - \bar{\mathcal{W}}_{g_1}(\tau)\right)\right]\times \\ \left[\displaystyle\int_0^1 \bar{\alpha}_0\,\mathcal{F}(|\eta-\eta'|;\bar{l}_s)\left(1+\frac{\partial \bar{\mathcal{X}}_p}{\partial \eta}\right)\frac{\mathrm{d}\phi_j^{\mathcal{Y}}}{\mathrm{d}\eta}\,\mathrm{d}\eta'\right] \end{array}\right\}\mathrm{d}\eta, \tag{3c}$$

$$\left[\frac{\partial \bar{\mathbf{p}}_b^{\mathcal{Y}}}{\partial \bar{\mathbf{y}}}\right]_{ij} = -\int_0^1 \left\{\begin{array}{l} \lambda^2(1+\chi_1)\,\dfrac{\mathrm{d}\phi_i^{\mathcal{Y}}}{\mathrm{d}\eta}\dfrac{\mathrm{d}\phi_j^{\mathcal{Y}}}{\mathrm{d}\eta}\,\left\{\displaystyle\int_0^1 \bar{\alpha}_0\,\mathcal{F}(|\eta-\eta'|;\bar{l}_s)\times\right. \\ \left.\left\{\left[\dfrac{\partial \bar{\mathcal{X}}_p}{\partial \eta} + \dfrac{\partial \bar{\mathcal{Y}}_p}{\partial \eta}\left(\bar{\mathcal{W}}_{g_2}(\tau) - \bar{\mathcal{W}}_{g_1}(\tau)\right)\right] + \dfrac{1}{2}\left[\left(\dfrac{\partial \bar{\mathcal{X}}_p}{\partial \eta}\right)^2 + \left(\dfrac{\partial \bar{\mathcal{Y}}_p}{\partial \eta}\right)^2\right]\right\}\right\}\mathrm{d}\eta' \\ +\lambda^2(1+\chi_1)\,\dfrac{\mathrm{d}\phi_i^{\mathcal{Y}}}{\mathrm{d}\eta}\,\left[\dfrac{\partial \bar{\mathcal{Y}}_p}{\partial \eta} + \left(\bar{\mathcal{W}}_{g_2}(\tau) - \bar{\mathcal{W}}_{g_1}(\tau)\right)\right]\times \\ \left\{\displaystyle\int_0^1 \bar{\alpha}_0\,\mathcal{F}(|\eta-\eta'|;\bar{l}_s)\left[\frac{\partial \bar{\mathcal{Y}}_p}{\partial \eta} + \left(\bar{\mathcal{W}}_{g_2}(\tau) - \bar{\mathcal{W}}_{g_1}(\tau)\right)\right]\frac{\mathrm{d}\phi_j^{\mathcal{Y}}}{\mathrm{d}\eta}\right\}\mathrm{d}\eta' \\ +\left[(1+\chi_3) + \chi_{22}(\lambda\beta_x)^2\right]\dfrac{\mathrm{d}^2\phi_i^{\mathcal{Y}}}{\mathrm{d}\eta^2}\,\left[\displaystyle\int_0^1 \bar{\alpha}_0\,\mathcal{F}(|\eta-\eta'|;\bar{l}_s)\frac{\mathrm{d}^2\phi_j^{\mathcal{Y}}}{\mathrm{d}\eta^2}\,\mathrm{d}\eta'\right] \\ +(\lambda\beta_x)^2\left[(1+\chi_0)\,\phi_i^{\mathcal{Y}}\,\dfrac{\mathrm{d}^2\phi_j^{\mathcal{Y}}}{\mathrm{d}\eta^2} + (1+\chi_{21})\lambda^{-2}\,\dfrac{\mathrm{d}\phi_i^{\mathcal{Y}}}{\mathrm{d}\eta}\,\dfrac{\mathrm{d}^3\phi_j^{\mathcal{Y}}}{\mathrm{d}\eta^3}\right] + \chi_4\,\dfrac{\mathrm{d}\phi_i^{\mathcal{Y}}}{\mathrm{d}\eta}\dfrac{\mathrm{d}\phi_j^{\mathcal{Y}}}{\mathrm{d}\eta} \end{array}\right\}\mathrm{d}\eta, \tag{3d}$$

$$\left[\frac{\partial \bar{\mathbf{p}}_b^{\mathcal{X}}}{\partial \bar{\mathbf{x}}_{,\tau}}\right]_{ij} = -2\lambda\beta_x\,(1+\chi_0)\int_0^1 \phi_i^{\mathcal{X}}\frac{\mathrm{d}\phi_j^{\mathcal{X}}}{\mathrm{d}\eta}\,\mathrm{d}\eta, \tag{3e}$$

$$\left[\frac{\partial \bar{\mathbf{p}}_b^{\mathcal{Y}}}{\partial \bar{\mathbf{y}}_{,\tau}}\right]_{ij} = -2\lambda\beta_x \int_0^1 \left\{ \begin{array}{l} (1+\chi_0)\phi_i^{\mathcal{Y}} \dfrac{\mathrm{d}\phi_j^{\mathcal{Y}}}{\mathrm{d}\eta} + \lambda^{-2}\,(1+\chi_2)\,\dfrac{\mathrm{d}\phi_i^{\mathcal{Y}}}{\mathrm{d}\eta}\dfrac{\mathrm{d}^2\phi_j^{\mathcal{Y}}}{\mathrm{d}\eta^2} \\ +\chi_{22}\,\dfrac{\mathrm{d}^2\phi_i^{\mathcal{Y}}}{\mathrm{d}\eta^2}\,\left[\displaystyle\int_0^1 \bar{\alpha}_0\,\mathcal{F}(|\eta-\eta'|;\bar{l}_s)\dfrac{\mathrm{d}\phi_j^{\mathcal{Y}}}{\mathrm{d}\eta}\,\mathrm{d}\eta'\right] \end{array} \right\} \mathrm{d}\eta. \tag{3f}$$

S.3. Analytical dynamic analysis of stationary nanochassis under arbitrarily excited ends via NDS

S.3.1. Evaluation of the nonlocal-surface energy-based elastic fields

By adopting the linear version of Eq. (31) (i.e., small deflection as well as ignoring the longitudinal and transverse interactions) for the case of stationary nanochassis (i.e., $\mathcal{V}_s$=0), the governing equation associated with the dynamic deflection of the excited nanochassis due to ends' excitations reads:

$$\begin{aligned}&(1+\chi_0)\left(\frac{\partial^2\bar{\mathcal{Y}}_p}{\partial\tau^2}-\mu^2\frac{\partial^4\bar{\mathcal{Y}}_p}{\partial\eta^4}\right)-\lambda^{-2}(1+\chi_2)\left(\frac{\partial^4\bar{\mathcal{Y}}_p}{\partial\tau^2\partial\eta^2}-\mu^2\frac{\partial^6\bar{\mathcal{Y}}_p}{\partial\tau^2\partial\eta^4}\right)-\chi_4\frac{\partial^2\bar{\mathcal{Y}}_p}{\partial\eta^2}\\&+(1+\chi_3)\frac{\partial^4\bar{\mathcal{Y}}_p}{\partial\eta^4}=-(1+\chi_0)\left[\left(\frac{\mathrm{d}^2\bar{\mathcal{W}}_{g_2}}{\mathrm{d}\tau^2}-\frac{\mathrm{d}^2\bar{\mathcal{W}}_{g_1}}{\mathrm{d}\tau^2}\right)\eta+\frac{\mathrm{d}^2\bar{\mathcal{W}}_{g_1}}{\mathrm{d}\tau^2}\right],\end{aligned}\tag{4}$$

with the following initial and boundary conditions:

$$\begin{aligned}&\bar{\mathcal{Y}}_p(\eta,0)=-\left[\left(\bar{\mathcal{W}}_{g_2}(0)-\bar{\mathcal{W}}_{g_1}(0)\right)\eta+\bar{\mathcal{W}}_{g_1}(0)\right],\\&\frac{\partial\bar{\mathcal{Y}}_p}{\partial\tau}(\eta,0)=-\left[\left(\frac{\mathrm{d}\bar{\mathcal{W}}_{g_2}}{\mathrm{d}\tau}(0)-\frac{\mathrm{d}\bar{\mathcal{W}}_{g_1}}{\mathrm{d}\tau}(0)\right)\eta+\frac{\mathrm{d}\bar{\mathcal{W}}_{g_1}}{\mathrm{d}\tau}(0)\right],\end{aligned}\tag{5a}$$

$$\begin{aligned}&\bar{\mathcal{Y}}_p(0,\tau)=0,\ \ \bar{\mathcal{Y}}_p(1,\tau)=0,\\&\bar{\mathcal{M}}^{nl}_{y,t}(0,\tau)=0,\ \ \bar{\mathcal{M}}^{nl}_{y,t}(1,\tau)=0.\end{aligned}\tag{5b}$$

To solve Eq. (4) for the given conditions in Eqs. (5a) and (4b), let us consider the purely dynamic deflection as follows:

$$\bar{\mathcal{Y}}_p(\eta,\tau)=\sum_{n=1}^{\infty}\bar{a}_n(\tau)\sin(n\pi\eta),\tag{6}$$

where $\bar{a}_n(\tau)$ are the time-dependent factors or the coefficients of the Fourier sine series of $\bar{\mathcal{Y}}_p$. By this choice of discretization, all the boundary conditions provided in Eq. (5b) are appropriately satisfied. On the other hand, one can readily write the Fourier sine series of the functions 1 and η as:

$$1=\sum_{n=1}^{\infty}\left[\frac{2}{n\pi}\left(1-\cos(n\pi)\right)\right]\sin(n\pi\eta),\quad \eta=\sum_{n=1}^{\infty}\left[-\frac{2}{n\pi}\cos(n\pi)\right]\sin(n\pi\eta),\tag{7}$$

through introducing Eqs. (6) and (7) to Eqs. (4) and (5), the following second-order ordinary differential equation is derived:

$$\frac{\mathrm{d}^2\bar{a}_n}{\mathrm{d}\tau^2} + \varpi_n^2 \bar{a}_n = \alpha_n \frac{\mathrm{d}^2\bar{\mathcal{W}}_{g_1}}{\mathrm{d}\tau^2} + \beta_n \frac{\mathrm{d}^2\bar{\mathcal{W}}_{g_2}}{\mathrm{d}\tau^2}; \quad \bar{a}_n = \bar{a}_n(\tau), \tag{8a}$$

$$\bar{a}_n(0) = \gamma_n \bar{\mathcal{W}}_{g_1}(0) + \zeta_n \bar{\mathcal{W}}_{g_2}(0), \quad \frac{\mathrm{d}\bar{a}_n}{\mathrm{d}\tau}(0) = \gamma_n \frac{\mathrm{d}\bar{\mathcal{W}}_{g_1}}{\mathrm{d}\tau}(0) + \zeta_n \frac{\mathrm{d}\bar{\mathcal{W}}_{g_2}}{\mathrm{d}\tau}(0), \tag{8b}$$

where the nth dimensionless natural frequency as well as other n-dependent parameters in Eq. (8) are defined by:

$$\begin{aligned}
\varpi_n^2 &= \frac{\chi_4(n\pi)^2 + (1+\chi_3)(n\pi)^4}{(1+(\mu n\pi)^2)\left[(1+\chi_0) + \lambda^{-2}(1+\chi_2)(n\pi)^2\right]}, \quad \gamma_n = -\frac{1}{n\pi}, \\
\alpha_n &= -\frac{2(1+\chi_0)}{n\pi\,(1+(\mu n\pi)^2)\left[(1+\chi_0) + \lambda^{-2}(1+\chi_2)(n\pi)^2\right]}, \quad \zeta_n = \frac{2(-1)^n}{n\pi}, \\
\beta_n &= \frac{2(-1)^n(1+\chi_0)}{n\pi\,(1+(\mu n\pi)^2)\left[(1+\chi_0) + \lambda^{-2}(1+\chi_2)(n\pi)^2\right]}.
\end{aligned} \tag{9}$$

An exact solution to Eq. (8a) could be sought as follows:

$$\bar{a}_n(\tau) = \mathcal{A}_n \cos(\varpi_n \tau) + \mathcal{B}_n \sin(\varpi_n \tau) + \int_0^\tau \left(\frac{\alpha_n \frac{\mathrm{d}^2\bar{\mathcal{W}}_{g_1}}{\mathrm{d}\tau^2} + \beta_n \frac{\mathrm{d}^2\bar{\mathcal{W}}_{g_2}}{\mathrm{d}\tau^2}}{M_n \varpi_n} \right) \sin\left(\varpi_n(\tau - \tau^*)\right) \mathrm{d}\tau^*, \tag{10}$$

by enforcing the conditions given in Eq. (8b), the values of the constants $\mathcal{A}_n$ and $\mathcal{B}_n$ are evaluated by:

$$\mathcal{A}_n = \gamma_n \bar{\mathcal{W}}_{g_1}(0) + \zeta_n \bar{\mathcal{W}}_{g_2}(0), \quad \mathcal{B}_n = \frac{\gamma_n}{\varpi_n} \frac{\mathrm{d}\bar{\mathcal{W}}_{g_1}}{\mathrm{d}\tau}(0) + \frac{\zeta_n}{\varpi_n} \frac{\mathrm{d}\bar{\mathcal{W}}_{g_2}}{\mathrm{d}\tau}(0), \tag{11}$$

and finally, by substituting the obtained parameters in Eq. (11) into Eq. (10) in view of Eq. (6), the dimensionless pure dynamic deflection of an stationary nanochassis due to its arbitrary supports' excitations takes the following form:

$$\bar{\mathcal{Y}}_p(\eta, \tau) = \sum_{n=1}^{\infty} \left(\begin{array}{l} \left[\gamma_n \bar{\mathcal{W}}_{g_1}(0) + \zeta_n \bar{\mathcal{W}}_{g_2}(0)\right] \cos(\varpi_n \tau) \\ + \left[\frac{\gamma_n}{\varpi_n} \frac{\mathrm{d}\bar{\mathcal{W}}_{g_1}}{\mathrm{d}\tau}(0) + \frac{\zeta_n}{\varpi_n} \frac{\mathrm{d}\bar{\mathcal{W}}_{g_2}}{\mathrm{d}\tau}(0)\right] \sin(\varpi_n \tau) \\ + \int_0^\tau \left(\frac{\alpha_n \frac{\mathrm{d}^2\bar{\mathcal{W}}_{g_1}}{\mathrm{d}\tau^2} + \beta_n \frac{\mathrm{d}^2\bar{\mathcal{W}}_{g_2}}{\mathrm{d}\tau^2}}{M_n \varpi_n} \right) \sin\left(\varpi_n(\tau - \tau^*)\right) \mathrm{d}\tau^* \end{array} \right) \sin(n\pi\eta), \tag{12}$$

where the parameter M_n, the so-called dimensionless effective mass of the nth vibration mode, is defined by:

$$M_n = \left(1 + (\mu n\pi)^2\right) \left[(1+\chi_0) + (1+\chi_2)\left(\frac{n\pi}{\lambda}\right)^2\right]. \tag{13}$$

Now by substituting Eq. (12) into Eq. (33b), the total surface energy-based bending moment within the excited nanochassis in the dimensionless form is obtained as:

$$\begin{aligned}\bar{\mathcal{M}}_{y,t}^{nl}(\eta,\tau) = & \sum_{n=1}^{\infty} \begin{pmatrix} (n\pi)^2 (1+\chi_3)\, \bar{a}_n(\tau) \\ \left[\mu^2 \left((1+\chi_0) + \left(\frac{n\pi}{\lambda}\right)^2 (1+\chi_2+\chi_5)\right) - \frac{\chi_5}{\lambda^2}\right] \frac{\mathrm{d}^2\bar{a}_n}{\mathrm{d}\tau^2} \end{pmatrix} \sin(n\pi\eta) + \\ & \left(\mu^2(1+\chi_0) - \frac{\chi_5}{\lambda^2}\right) \left[\left(\frac{\mathrm{d}^2\bar{\mathcal{W}}_{g_2}}{\mathrm{d}\tau^2} - \frac{\mathrm{d}^2\bar{\mathcal{W}}_{g_1}}{\mathrm{d}\tau^2}\right)\eta + \frac{\mathrm{d}^2\bar{\mathcal{W}}_{g_1}}{\mathrm{d}\tau^2}\right],\end{aligned} \tag{14}$$

where the first and the second derivatives of $\bar{a}_n = \bar{a}_n(\tau)$, which are required in computing the lateral velocity and dynamic flexural moment of the excited nanochassis, are calculated by utilizing the Leibniz integral rule as follows:

$$\begin{aligned}\frac{\mathrm{d}\bar{a}_n}{\mathrm{d}\tau} = & -\varpi_n \left(\gamma_n \bar{\mathcal{W}}_{g_1}(0) + \zeta_n \bar{\mathcal{W}}_{g_2}(0)\right) \sin(\varpi_n\tau) \\ & + \left(\gamma_n \frac{\mathrm{d}\bar{\mathcal{W}}_{g_1}}{\mathrm{d}\tau}(0) + \zeta_n \frac{\mathrm{d}\bar{\mathcal{W}}_{g_2}}{\mathrm{d}\tau}(0)\right) \cos(\varpi_n\tau) \\ & + \int_0^{\tau} \left[\begin{array}{l} \left(\dfrac{\alpha_n \frac{\mathrm{d}^3\bar{\mathcal{W}}_{g_1}}{\mathrm{d}\tau^3} + \beta_n \frac{\mathrm{d}^3\bar{\mathcal{W}}_{g_2}}{\mathrm{d}\tau^3}}{M_n\varpi_n}\right) \sin\left(\varpi_n(\tau-\tau^*)\right) + \\ \left(\dfrac{\alpha_n}{M_n}\dfrac{\mathrm{d}^2\bar{\mathcal{W}}_{g_1}}{\mathrm{d}\tau^2} + \dfrac{\beta_n}{M_n}\dfrac{\mathrm{d}^2\bar{\mathcal{W}}_{g_2}}{\mathrm{d}\tau^2}\right) \cos\left(\varpi_n(\tau-\tau^*)\right) \end{array}\right] \mathrm{d}\tau^*,\end{aligned} \tag{15a}$$

$$\begin{aligned}\frac{\mathrm{d}^2\bar{a}_n}{\mathrm{d}\tau^2} = & -\varpi_n^2 \left(\gamma_n \bar{\mathcal{W}}_{g_1}(0) + \zeta_n \bar{\mathcal{W}}_{g_2}(0)\right) \cos(\varpi_n\tau) + \frac{\alpha_n}{M_n}\frac{\mathrm{d}^2\bar{\mathcal{W}}_{g_1}}{\mathrm{d}\tau^2} \\ & + \varpi_n \left(\gamma_n \frac{\mathrm{d}\bar{\mathcal{W}}_{g_1}}{\mathrm{d}\tau}(0) + \zeta_n \frac{\mathrm{d}\bar{\mathcal{W}}_{g_2}}{\mathrm{d}\tau}(0)\right) \cos(\varpi_n\tau) + \frac{\beta_n}{M_n}\frac{\mathrm{d}^2\bar{\mathcal{W}}_{g_2}}{\mathrm{d}\tau^2} \\ & + \int_0^{\tau} \left[\begin{array}{l} \left(\dfrac{\alpha_n}{M_n\varpi_n}\dfrac{\mathrm{d}^4\bar{\mathcal{W}}_{g_1}}{\mathrm{d}\tau^4} + \dfrac{\beta_n}{M_n\varpi_n}\dfrac{\mathrm{d}^4\bar{\mathcal{W}}_{g_2}}{\mathrm{d}\tau^4}\right) \sin\left(\varpi_n(\tau-\tau^*)\right) + \\ 2\left(\dfrac{\alpha_n}{M_n}\dfrac{\mathrm{d}^3\bar{\mathcal{W}}_{g_1}}{\mathrm{d}\tau^3} + \dfrac{\beta_n}{M_n}\dfrac{\mathrm{d}^3\bar{\mathcal{W}}_{g_2}}{\mathrm{d}\tau^3}\right) \cos\left(\varpi_n(\tau-\tau^*)\right) \\ -\varpi_n \left(\dfrac{\alpha_n}{M_n}\dfrac{\mathrm{d}^2\bar{\mathcal{W}}_{g_1}}{\mathrm{d}\tau^2} + \dfrac{\beta_n}{M_n}\dfrac{\mathrm{d}^2\bar{\mathcal{W}}_{g_2}}{\mathrm{d}\tau^2}\right) \cos\left(\varpi_n(\tau-\tau^*)\right) \end{array}\right] \mathrm{d}\tau^*.\end{aligned} \tag{15b}$$

The developed analytical solution in this part would be very beneficial in dynamic analysis of some interesting and basic problems arise in applied nanomechanics, including a stationary nanochassis acted upon by harmonic excitations at its ends, a movable nanowire on a harmonic surface with a fairly low velocity, and their possible instabilities. In the following parts, we briefly analyze such problems.

S.3.2. A stationary nanochassis subjected to the ends harmonic excitation

For the case of the dissimilar harmonic excitations at the ends of the stationary nanochassis per following:

$$\bar{\mathcal{W}}_{g1} = \bar{a}_1 \sin(\bar{\varpi}_1\tau + \phi_1), \quad \bar{\mathcal{W}}_{g2} = \bar{a}_2 \sin(\bar{\varpi}_2\tau + \phi_2), \tag{16}$$

where $\bar{a}_i$, $\bar{\varpi}_i$, and ϕ_i in order are the dimensionless amplitudes, dimensionless frequencies, and phase lags of the excited ends of the nanochassis such that these dimensionless factors are related to their corresponding dimensional ones by $a_i = \bar{a}_i\, l_b$ and $\varpi_i = \dfrac{\bar{\varpi}_i}{l_b^2}\sqrt{\dfrac{E_bI_b}{\rho_bA_b}}$, where a_i and ϖ_i represent the amplitude and excited frequency of the ith end, respectively. By substituting Eq. (16) into Eqs. (12) and (14) in view of Eqs. (15a) and (15b), the dimensionless dynamical deflection as well as the dimensionless total flexural moment within the excited beam-like nanochassis are derived as follows:

$$\bar{\mathcal{Y}}_p(\eta,\tau) = \sum_{n=1}^{\infty} \left(\begin{array}{l} (\gamma_n\, \bar{a}_1 \sin(\phi_1) + \zeta_n\, \bar{a}_2 \sin(\phi_2)) \cos(\varpi_n\tau) \\ + \left(\dfrac{\bar{a}_1\gamma_n\bar{\varpi}_1}{\varpi_n} \cos(\phi_1) + \dfrac{\bar{a}_2\zeta_n\bar{\varpi}_2}{\varpi_n} \cos(\phi_2) \right) \sin(\varpi_n\tau) \\ -\dfrac{\alpha_n\bar{\varpi}_1^2\bar{a}_1}{2M_n\varpi_n} \left\{ \begin{array}{l} \dfrac{1}{\bar{\varpi}_1+\varpi_n}\,[\sin(\bar{\varpi}_1\tau+\phi_1)+\sin(\varpi_n\tau-\varphi_1)] \\ -\dfrac{1}{\bar{\varpi}_1-\varpi_n}\,[\sin(\bar{\varpi}_1\tau+\phi_1)-\sin(\varpi_n\tau+\varphi_1)] \end{array} \right\} \\ -\dfrac{\beta_n\bar{\varpi}_2^2\bar{a}_2}{2M_n\varpi_n} \left\{ \begin{array}{l} \dfrac{1}{\bar{\varpi}_2+\varpi_n}\,[\sin(\bar{\varpi}_2\tau+\phi_2)+\sin(\varpi_n\tau-\varphi_2)] \\ -\dfrac{1}{\bar{\varpi}_2-\varpi_n}\,[\sin(\bar{\varpi}_2\tau+\phi_2)-\sin(\varpi_n\tau+\varphi_2)] \end{array} \right\} \end{array} \right) \sin(n\pi\eta), \tag{17}$$

and

$$
\begin{aligned}
&\bar{\mathcal{M}}^{nl}_{y,t}(\eta,\tau) = \\
&\sum_{n=1}^{\infty}\left\{
\begin{array}{l}
(n\pi)^2\,(1+\chi_3)\times \\
\left(
\begin{array}{l}
(\gamma_n\,\bar{a}_1\sin(\phi_1)+\zeta_n\,\bar{a}_2\sin(\phi_2))\cos(\varpi_n\tau) \\
+\left(\dfrac{\bar{a}_1\gamma_n\bar{\varpi}_1}{\varpi_n}\cos(\phi_1)+\dfrac{\bar{a}_2\zeta_n\bar{\varpi}_2}{\varpi_n}\cos(\phi_2)\right)\sin(\varpi_n\tau) \\
-\dfrac{\alpha_n\bar{\varpi}_1^2\bar{a}_1}{2M_n\varpi_n}\left\{
\begin{array}{l}
\dfrac{1}{\bar{\varpi}_1+\varpi_n}\,[\sin(\bar{\varpi}_1\tau+\phi_1)+\sin(\varpi_n\tau-\varphi_1)] \\
-\dfrac{1}{\bar{\varpi}_1-\varpi_n}\,[\sin(\bar{\varpi}_1\tau+\phi_1)-\sin(\varpi_n\tau+\varphi_1)]
\end{array}\right\} \\
-\dfrac{\beta_n\bar{\varpi}_2^2\bar{a}_2}{2M_n\varpi_n}\left\{
\begin{array}{l}
\dfrac{1}{\bar{\varpi}_2+\varpi_n}\,[\sin(\bar{\varpi}_2\tau+\phi_2)+\sin(\varpi_n\tau-\varphi_2)] \\
-\dfrac{1}{\bar{\varpi}_2-\varpi_n}\,[\sin(\bar{\varpi}_2\tau+\phi_2)-\sin(\varpi_n\tau+\varphi_2)]
\end{array}\right\}
\end{array}
\right) \\
\left[\mu^2\left((1+\chi_0)+\left(\dfrac{n\pi}{\lambda}\right)^2(1+\chi_2+\chi_5)\right)-\lambda^{-2}\chi_5\right]\times \\
\left(
\begin{array}{l}
-\varpi_n^2\,(\gamma_n\,\bar{a}_1\sin(\phi_1)+\zeta_n\,\bar{a}_2\sin(\phi_2))\cos(\varpi_n\tau) \\
-\varpi_n\,(\bar{a}_1\gamma_n\bar{\varpi}_1\cos(\phi_1)+\bar{a}_2\zeta_n\bar{\varpi}_2\cos(\phi_2))\sin(\varpi_n\tau) \\
+\dfrac{\alpha_n\bar{\varpi}_1^2\bar{a}_1}{2M_n\varpi_n}\left\{
\begin{array}{l}
\dfrac{1}{\bar{\varpi}_1+\varpi_n}\,\left[\bar{\varpi}_1^2\sin(\bar{\varpi}_1\tau+\phi_1)+\varpi_n^2\sin(\varpi_n\tau-\varphi_1)\right] \\
-\dfrac{1}{\bar{\varpi}_1-\varpi_n}\,\left[\bar{\varpi}_1^2\sin(\bar{\varpi}_1\tau+\phi_1)-\varpi_n^2\sin(\varpi_n\tau+\varphi_1)\right]
\end{array}\right\} \\
+\dfrac{\beta_n\bar{\varpi}_2^2\bar{a}_2}{2M_n\varpi_n}\left\{
\begin{array}{l}
\dfrac{1}{\bar{\varpi}_2+\varpi_n}\,\left[\bar{\varpi}_2^2\sin(\bar{\varpi}_2\tau+\phi_2)+\varpi_n^2\sin(\varpi_n\tau-\varphi_2)\right] \\
-\dfrac{1}{\bar{\varpi}_2-\varpi_n}\,\left[\bar{\varpi}_2^2\sin(\bar{\varpi}_2\tau+\phi_2)-\varpi_n^2\sin(\varpi_n\tau+\varphi_2)\right]
\end{array}\right\}
\end{array}
\right)
\end{array}
\right\}\sin(n\pi\eta) \\
&+\left(\mu^2\,(1+\chi_0)-\frac{\chi_5}{\lambda^2}\right)\left[\left(\frac{\mathrm{d}^2\bar{\mathcal{W}}_{g_2}}{\mathrm{d}\tau^2}-\frac{\mathrm{d}^2\bar{\mathcal{W}}_{g_1}}{\mathrm{d}\tau^2}\right)\eta+\frac{\mathrm{d}^2\bar{\mathcal{W}}_{g_1}}{\mathrm{d}\tau^2}\right].
\end{aligned}
\tag{18}
$$

As it is seen from Eqs. (17) and (18), the application of these fields is limited to the cases of: $\bar{\varpi}_i \neq \varpi_n$, where i=1,2, and j=1,2,…,∞. In the following part, the possibility of dynamic instability of the excited nanochassis is displayed.

S.3.3. Potential instability of the excited stationary nanochassis (resonance state)

It can be easily researched that if the excited frequency of the L.H.S. end of the stationary nanochassis would be equal to its jth natural frequency (for example, $\bar{\varpi}_1 = \varpi_j$), the given expression for the jth time-dependent parameter based on Eq. (17) using the L'Hopital's

rule would be modified to:

$$\begin{aligned}
a_j(\tau) = \;& (\gamma_j\,\bar{a}_1\sin(\phi_1) + \zeta_j\,\bar{a}_2\sin(\phi_2))\cos(\varpi_j\tau) + \\
& \left(\frac{\bar{a}_1\gamma_j\bar{\varpi}_1}{\varpi_j}\cos(\phi_1) + \frac{\bar{a}_2\zeta_j\bar{\varpi}_2}{\varpi_j}\cos(\phi_2)\right)\sin(\varpi_j\tau) \\
& -\frac{\alpha_j\bar{\varpi}_1^2\bar{a}_1}{2M_j\varpi_j}\left[\frac{\sin(\varpi_j\tau)}{\varpi_j}\cos(\phi_1) - 2\tau\cos(\varpi_j\tau+\phi_1)\right] \\
& -\frac{\beta_j\bar{\varpi}_2^2\bar{a}_2}{2M_j\varpi_j}\left\{\begin{array}{l} \dfrac{1}{\bar{\varpi}_2+\varpi_j}\left[\sin(\bar{\varpi}_2\tau+\phi_2)+\sin(\varpi_j\tau-\varphi_2)\right] \\ -\dfrac{1}{\bar{\varpi}_2-\varpi_j}\left[\sin(\bar{\varpi}_2\tau+\phi_2)-\sin(\varpi_j\tau+\varphi_2)\right] \end{array}\right\},
\end{aligned} \tag{19}$$

as a result, the dimensionless deflection of the excited nanochassis is obtained as:

$$\begin{aligned}
\bar{\mathcal{Y}}_p(\eta,\tau) = \;& \sum_{n=1}^{\infty}\left(\begin{array}{l} (\gamma_n\,\bar{a}_1\sin(\phi_1)+\zeta_n\,\bar{a}_2\sin(\phi_2))\cos(\varpi_n\tau) \\ +\left(\dfrac{\bar{a}_1\gamma_n\bar{\varpi}_1}{\varpi_n}\cos(\phi_1)+\dfrac{\bar{a}_2\zeta_n\bar{\varpi}_2}{\varpi_n}\cos(\phi_2)\right)\sin(\varpi_n\tau) \\ -\dfrac{\beta_n\bar{\varpi}_2^2\bar{a}_2}{2M_n\varpi_n}\left\{\begin{array}{l} \dfrac{1}{\bar{\varpi}_2+\varpi_n}\left[\sin(\bar{\varpi}_2\tau+\phi_2)+\sin(\varpi_n\tau-\varphi_2)\right] \\ -\dfrac{1}{\bar{\varpi}_2-\varpi_n}\left[\sin(\bar{\varpi}_2\tau+\phi_2)-\sin(\varpi_n\tau+\varphi_2)\right] \end{array}\right\} \end{array}\right)\sin(n\pi\eta) \\
& -\frac{\alpha_j\bar{\varpi}_1^2\bar{a}_1}{2M_j\varpi_j}\left[\frac{\sin(\varpi_j\tau)}{\varpi_j}\cos(\phi_1) - \underline{\underline{2\tau\cos(\varpi_j\tau+\phi_1)}}\right]\sin(j\pi\eta),
\end{aligned} \tag{20}$$

further, the dimensionless flexural moment field of the excited beam-like nanochassis for this case would be stated by:

$$
\bar{\mathcal{M}}^{nl}_{y,t}(\eta,\tau) = \\
\sum_{n=1}^{\infty}\left\{\begin{array}{l}
(n\pi)^2\,(1+\chi_3)\times \\
\left(\begin{array}{l}
(\gamma_n\,\bar{a}_1\sin(\phi_1)+\zeta_n\,\bar{a}_2\sin(\phi_2))\cos(\varpi_n\tau) \\
+\left(\dfrac{\bar{a}_1\gamma_n\bar{\varpi}_1}{\varpi_n}\cos(\phi_1)+\dfrac{\bar{a}_2\zeta_n\bar{\varpi}_2}{\varpi_n}\cos(\phi_2)\right)\sin(\varpi_n\tau) \\
-\dfrac{\beta_n\bar{\varpi}_2^2\bar{a}_2}{2M_n\varpi_n}\left\{\begin{array}{l}
\dfrac{1}{\bar{\varpi}_2+\varpi_n}\left[\sin(\bar{\varpi}_2\tau+\phi_2)+\sin(\varpi_n\tau-\varphi_2)\right] \\
-\dfrac{1}{\bar{\varpi}_2-\varpi_n}\left[\sin(\bar{\varpi}_2\tau+\phi_2)-\sin(\varpi_n\tau+\varphi_2)\right]
\end{array}\right\}
\end{array}\right) \\
\left[\mu^2\left((1+\chi_0)+\left(\dfrac{n\pi}{\lambda}\right)^2(1+\chi_2+\chi_5)\right)-\lambda^{-2}\chi_5\right]\times \\
\left(\begin{array}{l}
-\varpi_n^2\,(\gamma_n\,\bar{a}_1\sin(\phi_1)+\zeta_n\,\bar{a}_2\sin(\phi_2))\cos(\varpi_n\tau) \\
-\varpi_n\,(\bar{a}_1\gamma_n\bar{\varpi}_1\cos(\phi_1)+\bar{a}_2\zeta_n\bar{\varpi}_2\cos(\phi_2))\sin(\varpi_n\tau) \\
+\dfrac{\beta_n\bar{\varpi}_2^2\bar{a}_2}{2M_n\varpi_n}\left\{\begin{array}{l}
\dfrac{1}{\bar{\varpi}_2+\varpi_n}\left[\bar{\varpi}_2^2\sin(\bar{\varpi}_2\tau+\phi_2)+\varpi_n^2\sin(\varpi_n\tau-\varphi_2)\right] \\
-\dfrac{1}{\bar{\varpi}_2-\varpi_n}\left[\bar{\varpi}_2^2\sin(\bar{\varpi}_2\tau+\phi_2)-\varpi_n^2\sin(\varpi_n\tau+\varphi_2)\right]
\end{array}\right\}
\end{array}\right)
\end{array}\right\}\sin(n\pi\eta) \\
+\left(\mu^2\,(1+\chi_0)-\frac{\chi_5}{\lambda^2}\right)\left[\left(\frac{\mathrm{d}^2\bar{\mathcal{W}}_{g_2}}{\mathrm{d}\tau^2}-\frac{\mathrm{d}^2\bar{\mathcal{W}}_{g_1}}{\mathrm{d}\tau^2}\right)\eta+\frac{\mathrm{d}^2\bar{\mathcal{W}}_{g_1}}{\mathrm{d}\tau^2}\right] \\
+\frac{\alpha_j\bar{\varpi}_1\bar{a}_1}{2M_j}\left\{\begin{array}{l}
\left[\mu^2\left((1+\chi_0)+\left(\dfrac{n\pi}{\lambda}\right)^2(1+\chi_2+\chi_5)\right)-\lambda^{-2}\chi_5\right]\times \\
\left[\begin{array}{l}
\varpi_j\sin(\varpi_j\tau)\cos(\phi_1)+2\varpi_j\times \\
\left(\sin(\varpi_j\tau+\phi_1)+\underline{\underline{\tau\cos(\varpi_j\tau+\phi_1)}}\right)
\end{array}\right] \\
+(j\pi)^2\,(1+\chi_3)\left[\dfrac{\sin(\varpi_j\tau)}{\varpi_j}\cos(\phi_1)-\underline{\underline{2\tau\cos(\varpi_j\tau+\phi_1)}}\right]
\end{array}\right\}\sin(j\pi\eta). \tag{21}
$$

The doubly underlined statements in Eqs. (20) and (21) clearly show that the deflection and the flexural moment each point of the excited nanochassis would increase as time goes by, until leading to the collapse of the excited nanostructure. This dynamic stability represents the *resonance state*. For the case of identical excitation at the ends of the nanochassis, these relations obviously display that the failure of the nanostructure will begin from its midspan point at the resonance state.

S.4. Exact linear free vibration of moving nanochassis accounting for surface effect

In this part, we are interested in exact free vibration analysis of axially moving nanochassis with simple ends for the first time. To this end, two cases-lengthy moving nanochassis and moving nanochassis with surface energy effect but without considering the nonlocality- are considered and their explicit characteristic equations are extracted wit some efforts. The main application of these crucial relations is in predicting the trends of the natural frequencies as a function of the velocity of the axially moving nanochassis. The complementary scrutiny leads to unveiling the divergence velocity of the moving nanostructure which is of grave importance in its mechanical analysis.

S.4.1. Free vibration of moving lengthy nanochassis

For fairly lengthy nanochassis, the term of the internal bending stiffness can be fairly neglected in compare to that of the geometrical stiffness (i.e., $\left|(1+\chi_3)\dfrac{\partial^4\bar{\mathcal{Y}}_p}{\partial\eta^4}\right| << \left|\chi_4\,\dfrac{\partial^2\bar{\mathcal{Y}}_p}{\partial\eta^2}\right|$), the rotary inertia term can be neglected in compare to the transverse inertia term (i.e., $\left|(1+\chi_0)\dfrac{\partial^2\bar{\mathcal{Y}}_p}{\partial\tau^2}\right| << \left|\lambda^{-2}(1+\chi_2)\dfrac{\partial^4\bar{\mathcal{Y}}_p}{\partial\tau^2\partial\eta^2}\right|$), and the nonlocality can be rationally ignored (i.e., $\mu \approx 0$). By adopting these assumptions for linear version of Eq. (31) for the case of a horizontal surface, the corresponding governing equation that displays transverse vibration of the moving lengthy nanochassis would take the following form:

$$(1+\chi_0)\left(\frac{\partial^2\bar{\mathcal{Y}}_p}{\partial\tau^2} + \underline{\underline{2\lambda\beta_x\,\frac{\partial^2\bar{\mathcal{Y}}_p}{\partial\tau\partial\eta}}} + (\lambda\beta_x)^2\,\frac{\partial^2\bar{\mathcal{Y}}_p}{\partial\eta^2}\right) - \chi_4\,\frac{\partial^2\bar{\mathcal{Y}}_p}{\partial\eta^2} = 0. \tag{22}$$

Due to the appearance of the Coriolis acceleration term in Eq. (22) (i.e., the underlined term), development of a straightforward solution to this equation for computing the natural frequencies would not be as easy as it can be imagined. To overcome this difficulty, let us to consider the dimensionless purely dynamic deflection as: $\bar{\mathcal{Y}}_p(\eta,\tau) = e^{\mathcal{A}\eta}\,\bar{\mathcal{Y}}_p^{*}(\eta,\tau)$, where $\mathcal{A}$ is a unknown that should be appropriately determined. By substituting this form of

displacement into Eq. (22), it is obtainable:

$$\frac{\partial^2 \bar{\mathcal{Y}}_p^*}{\partial \tau^2} + 2\lambda\beta_x \left(\mathcal{A}\frac{\partial \bar{\mathcal{Y}}_p^*}{\partial \tau} + \frac{\partial^2 \bar{\mathcal{Y}}_p^*}{\partial \tau \partial \eta} \right) + \left[(\lambda\beta_x)^2 - \frac{\chi_4}{1+\chi_0} \right] \left(\frac{\partial^2 \bar{\mathcal{Y}}_p^*}{\partial \eta^2} + 2\mathcal{A}\frac{\partial \bar{\mathcal{Y}}_p^*}{\partial \eta} + \mathcal{A}^2\, \bar{\mathcal{Y}}_p^* \right) = 0. \tag{23}$$

With regard to the simply supported ends of the moving nanochassis, we consider: $\bar{\mathcal{Y}}_p^*(\eta,\tau) = \bar{\mathcal{Y}}_{p0}^*\, e^{\mathrm{i}\varpi_n \tau}\, \sin(n\pi\eta)$ to examine its free vibration, where i=$\sqrt{-1}$, $\bar{\mathcal{Y}}_{p0}^*$ is a constant such that $e^{\mathcal{A}\eta}\, \bar{\mathcal{Y}}_{p0}^*$ represents the amplitude of the dimensionless deflection, and n denotes the mode number. By introducing this form of $\bar{\mathcal{Y}}_p^*$ to Eq. (23), one can arrive at the following relation:

$$\begin{aligned} &\sin(n\pi\eta) \left\{ -\varpi_n^2 + 2\lambda\beta_x\, \mathcal{A}\varpi_n \mathrm{i} + \left[(\lambda\beta_x)^2 - \frac{\chi_4}{1+\chi_0} \right] \left(-(n\pi)^2 + \mathcal{A}^2 \right) \right\} \\ &+ \cos(n\pi\eta) \left\{ 2\lambda\beta_x (n\pi)\varpi_n \mathrm{i} + 2\mathcal{A}(n\pi) \left[(\lambda\beta_x)^2 - \frac{\chi_4}{1+\chi_0} \right] \right\} = 0. \end{aligned} \tag{24}$$

By solving Eq. (24) for $\mathcal{A}$ and ϖ_n, the values of these factors are easily evaluated by:

$$\varpi_n = n\pi \sqrt{\frac{1+\chi_0}{\chi_4}} \left(\frac{\chi_4}{1+\chi_0} - (\lambda\beta_x)^2 \right), \quad \mathcal{A} = n\pi\lambda\beta_x \sqrt{\frac{1+\chi_0}{\chi_4}}\,\mathrm{i}. \tag{25}$$

The axial velocity leads to vanishing the natural frequencies, the so-called *divergence velocity*, is readily obtained by:

$$(\beta_x)_{cr} = \frac{1}{\lambda} \sqrt{\frac{\chi_4}{1+\chi_0}}, \tag{26}$$

or in the dimensional form as follows:

$$(\mathcal{V}_s)_{cr} = \sqrt{\frac{\tau_s A_s}{\rho_s A_s + \rho_b A_b}}. \tag{27}$$

S.4.2. Free vibration of moving nanochassis without considering the nonlocality

Through ignoring the nonlocality, the equation of motion associated with the free transverse vibration of the axially moving nanochassis based on Eq. (31b) is expressed by:

$$\begin{aligned} &(1+\chi_0) \left(\frac{\partial^2 \bar{\mathcal{Y}}_p}{\partial \tau^2} + 2\lambda\beta_x \frac{\partial^2 \bar{\mathcal{Y}}_p}{\partial \tau \eta} + (\lambda\beta_x)^2\, \frac{\partial^2 \bar{\mathcal{Y}}_p}{\partial \eta^2} \right) - \chi_4 \frac{\partial^2 \bar{\mathcal{Y}}_p}{\partial \eta^2} \\ &-\lambda^{-2} (1+\chi_2) \left(\frac{\partial^4 \bar{\mathcal{Y}}_p}{\partial \tau^2 \partial \eta^2} + 2\lambda\beta_x \frac{\partial^4 \bar{\mathcal{Y}}_p}{\partial \tau \eta^3} + (\lambda\beta_x)^2\, \frac{\partial^4 \bar{\mathcal{Y}}_p}{\partial \eta^4} \right) + (1+\chi_3) \frac{\partial^4 \bar{\mathcal{Y}}_p}{\partial \eta^4} = 0, \end{aligned} \tag{28}$$

by following up the developed technique in the previous part through discretizing the purely dynamic displacement by: $\bar{\mathcal{Y}}_p(\eta,\tau) = e^{\mathcal{A}\eta}\,\bar{\mathcal{Y}}_p^*(\eta,\tau)$, Eq. (28) is modified to the following:

$$\begin{aligned}
&(1+\chi_0)\left[\frac{\partial^2\bar{\mathcal{Y}}_p^*}{\partial\tau^2} + 2\lambda\beta_x\left(\frac{\partial^2\bar{\mathcal{Y}}_p^*}{\partial\tau\eta} + \mathcal{A}\,\frac{\partial\bar{\mathcal{Y}}_p^*}{\partial\tau}\right) + (\lambda\beta_x)^2\left(\frac{\partial^2\bar{\mathcal{Y}}_p^*}{\partial\eta^2} + 2\mathcal{A}\frac{\partial\bar{\mathcal{Y}}_p^*}{\partial\eta} + \mathcal{A}^2\bar{\mathcal{Y}}_p^*\right)\right]\\
&-\lambda^{-2}(1+\chi_2)\left[\begin{array}{l}\left(\dfrac{\partial^4\bar{\mathcal{Y}}_p^*}{\partial\tau^2\partial\eta^2} + 2\mathcal{A}\dfrac{\partial^3\bar{\mathcal{Y}}_p^*}{\partial\tau^2\partial\eta} + \mathcal{A}^2\dfrac{\partial^2\bar{\mathcal{Y}}_p^*}{\partial\tau^2}\right) + \\ 2\lambda\beta_x\left(\dfrac{\partial^4\bar{\mathcal{Y}}_p^*}{\partial\tau\partial\eta^3} + 3\mathcal{A}\dfrac{\partial^3\bar{\mathcal{Y}}_p^*}{\partial\tau\partial\eta^2} + 3\mathcal{A}^2\dfrac{\partial^2\bar{\mathcal{Y}}_p}{\partial\tau\partial\eta} + \mathcal{A}^3\dfrac{\partial\bar{\mathcal{Y}}_p^*}{\partial\tau}\right)\end{array}\right]\\
&-\chi_4\left(\frac{\partial^2\bar{\mathcal{Y}}_p^*}{\partial\eta^2} + 2\mathcal{A}\,\frac{\partial\bar{\mathcal{Y}}_p^*}{\partial\eta} + \mathcal{A}^2\bar{\mathcal{Y}}_p^*\right) + \left[(1+\chi_3) - (1+\chi_2)\beta_x^2\right]\times\\
&\left(\frac{\partial^4\bar{\mathcal{Y}}_p^*}{\partial\eta^4} + 4\mathcal{A}\frac{\partial^3\bar{\mathcal{Y}}_p^*}{\partial\eta^3} + 6\mathcal{A}^2\frac{\partial^2\bar{\mathcal{Y}}_p^*}{\partial\eta^2} + 4\mathcal{A}^3\frac{\partial\bar{\mathcal{Y}}_p}{\partial\eta} + \mathcal{A}^4\bar{\mathcal{Y}}_p^*\right) = 0.
\end{aligned} \tag{29}$$

Now by introducing $\bar{\mathcal{Y}}_p^*(\eta,\tau) = \mathcal{Y}_0^* e^{\mathrm{i}\varpi_n\tau}\sin(n\pi\eta)$ to Eq. (29), it is expressible:

$$\begin{aligned}
&\sin(n\pi\eta)\left\{\begin{array}{l}-\varpi_n^2(1+\chi_0) + 2\lambda\beta_x\mathcal{A}\varpi_n(1+\chi_0)\mathrm{i} + (\lambda\beta_x)^2\left(\mathcal{A}^2 - (n\pi)^2\right)\\ -\lambda^{-2}(1+\chi_2)\left[\varpi_n^2\left((n\pi)^2 - \mathcal{A}^2\right) + 2\lambda\beta_x\varpi_n\mathrm{i}\left(\mathcal{A}^3 - 3\mathcal{A}(n\pi)^2\right)\right]\\ +\chi_4\left((n\pi)^2 - \mathcal{A}^2\right) + \left[(1+\chi_3) - (1+\chi_2)\beta_x^2\right]\left((n\pi)^4 - 6(n\pi)^2\mathcal{A} + \mathcal{A}^4\right)\end{array}\right\}\\
&+\cos(n\pi\eta)\left\{\begin{array}{l}(1+\chi_0)\left[2\lambda\beta_x(n\pi)\varpi_n\mathrm{i} + 2\mathcal{A}(n\pi)(\lambda\beta_x)^2\right]\\ -\lambda^{-2}(1+\chi_2)\left[-2\mathcal{A}\varpi_n^2(n\pi) - 2\lambda\beta_x\left((n\pi)^3 - 3\mathcal{A}^2\right)\varpi_n\mathrm{i}\right]\\ -2\mathcal{A}(n\pi)\chi_4 + \left[(1+\chi_3) - (1+\chi_2)\beta_x^2\right]\left(4\mathcal{A}^3(n\pi) - 4\mathcal{A}(n\pi)^3\right)\end{array}\right\} = 0.
\end{aligned} \tag{30}$$

By solving Eq. (30) for ϖ_n and $\mathcal{A}$ simultaneously, the following set of nonlinear equations are derived:

$$\mathcal{P}_{3n}\mathcal{A}^3 + \mathcal{P}_{2n}\mathcal{A}^2 + \mathcal{P}_{1n}\mathcal{A} + \mathcal{P}_{0n} = 0, \tag{31a}$$

$$\mathcal{Q}_{2n}\varpi_n^2 + \mathcal{Q}_{1n}\varpi_n + \mathcal{Q}_{0n} = 0, \tag{31b}$$

where the expressions of $\mathcal{P}_{in} = \mathcal{P}_{in}(\varpi_n)$; $i = 0, 1, 2, 3$ and $\mathcal{Q}_{jn} = \mathcal{Q}_{jn}(\mathcal{A})$; $j = 0, 1, 2$ are provided in the following:

$$\mathcal{P}_{3n}(\varpi_n) = 4(n\pi)\left[(1+\chi_3) - (1+\chi_2)\beta_x^2\right], \tag{32a}$$

$$\mathcal{P}_{2n}(\varpi_n) = -6\lambda^{-1}\beta_x(1+\chi_2)(n\pi)\varpi_n \mathrm{i}, \tag{32b}$$

$$\begin{aligned}\mathcal{P}_{1n}(\varpi_n) = \;& 2(n\pi)\left[(1+\chi_0)(\lambda\beta_x)^2 + \lambda^{-2}(1+\chi_2)\varpi_n^2\right](n\pi) \\ & -2(n\pi)\chi_4 - 4(n\pi)^3\left[(1+\chi_3) - (1+\chi_2)\beta_x^2\right],\end{aligned} \tag{32c}$$

$$\mathcal{P}_{0n}(\varpi_n) = 2\lambda\beta_x\varpi_n(n\pi)\left[(1+\chi_0) + (1+\chi_2)\left(\frac{n\pi}{\lambda}\right)^2\right]\mathrm{i}, \tag{32d}$$

$$\mathcal{Q}_{2n}(\mathcal{A}) = -(1+\chi_0) - \lambda^{-2}(1+\chi_2)\left((n\pi)^2 - \mathcal{A}^2\right), \tag{32e}$$

$$\mathcal{Q}_{1n}(\mathcal{A}) = 2\lambda\beta_x\mathcal{A}\left[(1+\chi_0) - (\mathcal{A}^2 - 3(n\pi)^2)(1+\chi_2)\lambda^{-2}\right]\mathrm{i}, \tag{32f}$$

$$\begin{aligned}\mathcal{Q}_{0n}(\mathcal{A}) = \;& \left(\chi_4 - (1+\chi_2)(\lambda\beta_x)^2\right)\left((n\pi)^2 - \mathcal{A}^2\right) \\ & + \left[(1+\chi_3) - (1+\chi_2)\beta_x^2\right]\left(\mathcal{A}^4 - 6\mathcal{A}^2(n\pi)^2 + (n\pi)^4\right).\end{aligned} \tag{32g}$$

By solving the set of coupled nonlinear relations in Eqs. (31a) and (31b) for given pair values of n and β_x, the values of $\mathcal{A}$ and ϖ_n can be easily evaluated, and the crucial plots of ϖ_n-β_x are readily extracted for the nth mode of vibration of the moving beam-like nanostructure.
